# Cooperativity enables widespread role of low-affinity motifs in chromatin accessibility and increases regulatory potential

**DOI:** 10.1016/j.xgen.2026.101339

**Published:** 2026-08-25

**Authors:** Melanie Weilert, Kaelan J. Brennan, Khyati Dalal, Sabrina Krueger, Haining Jiang, Rosa Martinez-Corral, Julia Zeitlinger

**Affiliations:** 1 Stowers Institute for Medical Research, Kansas City, MO, USA; 2 Department of Pathology & Laboratory Medicine, The University of Kansas Medical Center, Kansas City, KS, USA; 3 CRG (Barcelona Collaboratorium for Modelling and Predictive Biology), Barcelona, Spain

**Keywords:** interpretable deep learning, chromatin accessibility, low-affinity motifs, mouse embryonic stem cells, *cis*-regulatory encoding, pioneer transcription factors, gene regulation, mathematical modeling

## Abstract

Low-affinity transcription-factor (TF) motifs are an important element of the *cis*-regulatory code, yet they are notoriously difficult to map and mechanistically incompletely understood, limiting our ability to interpret non-coding variation in development, evolution, and disease. Here, we investigate their role in pioneering and leverage sequence-to-profile models of chromatin accessibility in mouse embryonic stem cells to reliably map and interpret low-affinity motifs across the genome. We find that low-affinity motifs have outsized effects by cooperating with nearby motifs through intra-nucleosomal soft syntax. By modeling nucleosome-mediated cooperativity with a kinetic model, we discover and validate that pioneer cooperativity makes a motif operate at higher pioneering ranges across changing TF concentrations, thereby raising the regulatory potential. These results show that low-affinity motifs can be accurately mapped, shape the properties of developmental enhancers, and likely play a widespread role in fine-tuning enhancers during evolution.

## Highlights

• Deep learning maps functional low-affinity motifs in mouse embryonic stem cells
• Low-affinity motifs leverage cooperativity with other motifs to pioneer chromatin
• Pioneer cooperativity occurs at flexible, intra-nucleosomal distances
• Pioneer cooperativity sensitizes enhancers to changing TF concentrations

## Graphical abstract

**Figure:**
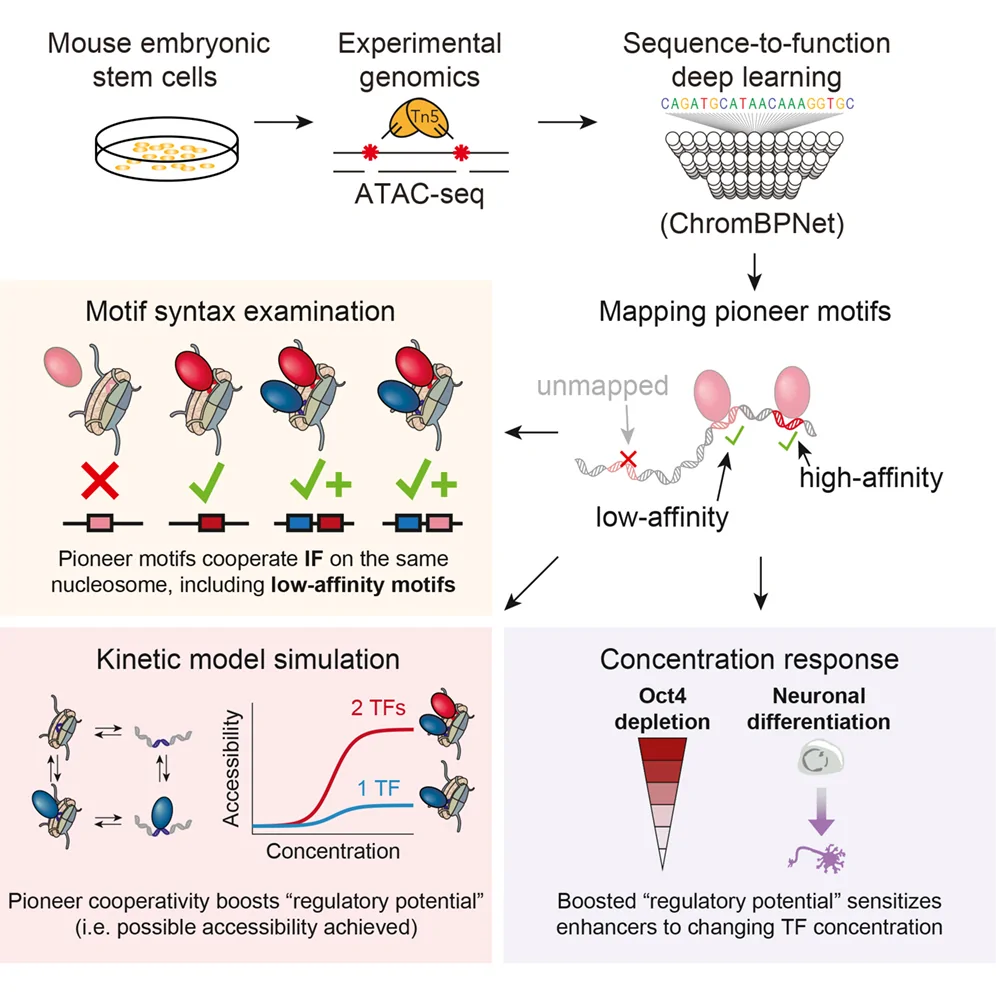

## Teaser

This study combines sequence-to-function models, experiments, and kinetic modeling to investigate the role of low-affinity transcription factor binding motifs in pioneering chromatin accessibility. It discovers that low-affinity motifs become functional by cooperating with nearby motifs through distance-dependent soft syntax and that this cooperativity influences enhancer activation and gene expression in a concentration-dependent manner.

## Introduction

One of the unresolved fundamental problems in biology is the *cis*-regulatory code, which, if mapped and understood, could unlock the gene-regulatory information encoded in the human genome.^1^^,^^2^ Mutations in transcription-factor (TF) binding motifs can cause phenotypic changes, making it necessary to obtain comprehensive maps of all functional motifs across cell types to predict the effect of non-coding genetic variants.^3^ A key challenge is to map low-affinity motifs, which are often critical for the specificity of enhancers during development.^4^^,^^5^^,^^6^^,^^7^^,^^8^^,^^9^^,^^10^ These motifs allow an enhancer to selectively become active when a certain combination of TFs are present, and their mutation can cause phenotypic effects.^6^^,^^7^

Mapping functional low-affinity motifs across the genome is challenging. Since they are defined by weak TF binding strength *in vitro* and low match scores to a position weight matrix (PWM), local sequence scanning produces too many false positives.^11^^,^^12^^,^^13^^,^^14^ Mapping low-affinity motifs therefore requires an understanding of the sequence context in which they become functional. Traditional mechanistic studies have mostly focused on DNA-mediated TF binding cooperativity, which occurs at close distances of less than ∼30 bp and can be examined with *in vitro* TF binding assays.^6^^,^^9^^,^^15^^,^^16^^,^^17^ How low-affinity motifs contribute to enhancer function beyond TF binding cooperativity has remained a gap in knowledge.

Here, we studied the role of low-affinity motifs in pioneering—the first step in enhancer activation where pioneer TFs make nucleosomal DNA accessible in chromatin,^18^ a process that may involve cooperativity^19^^,^^20^ or low-affinity motifs.^19^^,^^21^^,^^22^^,^^23^ However, pioneering effects vary widely across genomic regions and cell types, making it difficult to derive the underlying sequence rules.^24^^,^^25^ Furthermore, the definition of a pioneer TF and its mechanisms of cooperativity are debatable. We focus here on a single widely studied cell type, mouse embryonic stem cells (mESCs), and define a pioneer TF as any TF whose motif has a measurable effect on chromatin accessibility *in vivo*. This lens allows us to specifically assess the role of low-affinity motifs in pioneering.

An open question is whether low-affinity motifs confer specific properties to enhancers. Since low-affinity motifs are bound at higher TF concentrations,^10^ they allow developmental enhancers to respond at higher TF concentration ranges during embryonic development,^9^^,^^26^^,^^27^^,^^28^^,^^29^^,^^30^ making them susceptible to TF dosage-dependent phenotypic changes.^27^ However, if low-affinity motifs rely on cooperativity with other motifs, this could also affect an enhancer’s properties. However, how to rigorously distinguish the effects of motif affinity from cooperative interactions across the genome *in vivo* is not clear.

Sequence-to-function deep-learning models have majorly advanced functional motif mapping and syntax rule identification.^1^^,^^31^^,^^32^ The models are trained to accurately predict genomics data from DNA sequences, learning motifs combinatorially without prior biological assumptions. *Post hoc* interpretation tools then can extract motif instances and syntax rules.^33^ This approach has generated motif maps explaining TF binding,^34^^,^^35^^,^^36^ chromatin accessibility,^19^^,^^21^^,^^27^^,^^37^^,^^38^^,^^39^ reporter expression,^40^^,^^41^ promoter activity,^42^^,^^43^^,^^44^ and cell-type-specific enhancer activity *in vivo*.^45^^,^^46^^,^^47^

Despite high predictive accuracy of these models, the underlying molecular mechanisms that govern the functional outcomes of these syntax relationships are only beginning to emerge.^21^^,^^27^^,^^35^^,^^36^^,^^48^^,^^49^^,^^50^ Mechanistic interpretations of the *cis*-regulatory code learned by sequence-to-function models, including the role of low-affinity motifs, remain limited.

To investigate the role of low-affinity motifs in mediating pioneering in mESCs, we analyzed ChromBPNet models that predict bias-free chromatin accessibility at base resolution from genomic sequences.^38^ By interpreting these models, we quantify the contribution of low-affinity motifs to pioneering, define the cooperativity syntax, and use the cooperative measurements to model the response to TF concentration changes. We show that cooperativity shifts a motif to higher pioneering ranges, a property that we validate with *in vivo* time-course experiments.

## Results

### Accessibility deep-learning models enable the accurate mapping of low-affinity pioneer motifs in mESCs

To learn functional low-affinity motifs, we trained a ChromBPNet model that accurately predicts chromatin accessibility profiles in mESCs from DNA sequences^38^^,^^51^ (Figures 1A, 1B, and S1A; STAR Methods). To independently discover, compare, and validate the TF motifs, we trained a separate BPNet model on high-resolution TF binding data (chromatin immunoprecitation experiments with nucleotide resolution through exonuclease, unique barcode and single ligation [ChIP-nexus]) for Oct4, Sox2, Nanog, Klf4, and Zic3 in mESCs^36^^,^^51^ (Figures 1A and 1B). We ensured that both models could learn the same motifs by training on the same 154,827 1-kb-long output genomic coordinates (Figure 1B; STAR Methods), optimizing and cross-validating across different regions sets and training combinations (Table S1. Performances of deep-learning model optimization, related to Figure 1, Table S2. Performances of final deep-learning models, related to Figure 1, Table S3. TF-MoDISco motifs returned by deep-learning models, related to Figure 1; STAR Methods). Contribution scores were obtained using DeepSHAP, clustered by TF-MoDISco into motifs, and represented as either contribution weight matrices (CWMs) or PWMs.^36^^,^^51^^,^^52^^,^^53^^,^^54^

**Figure 1:**
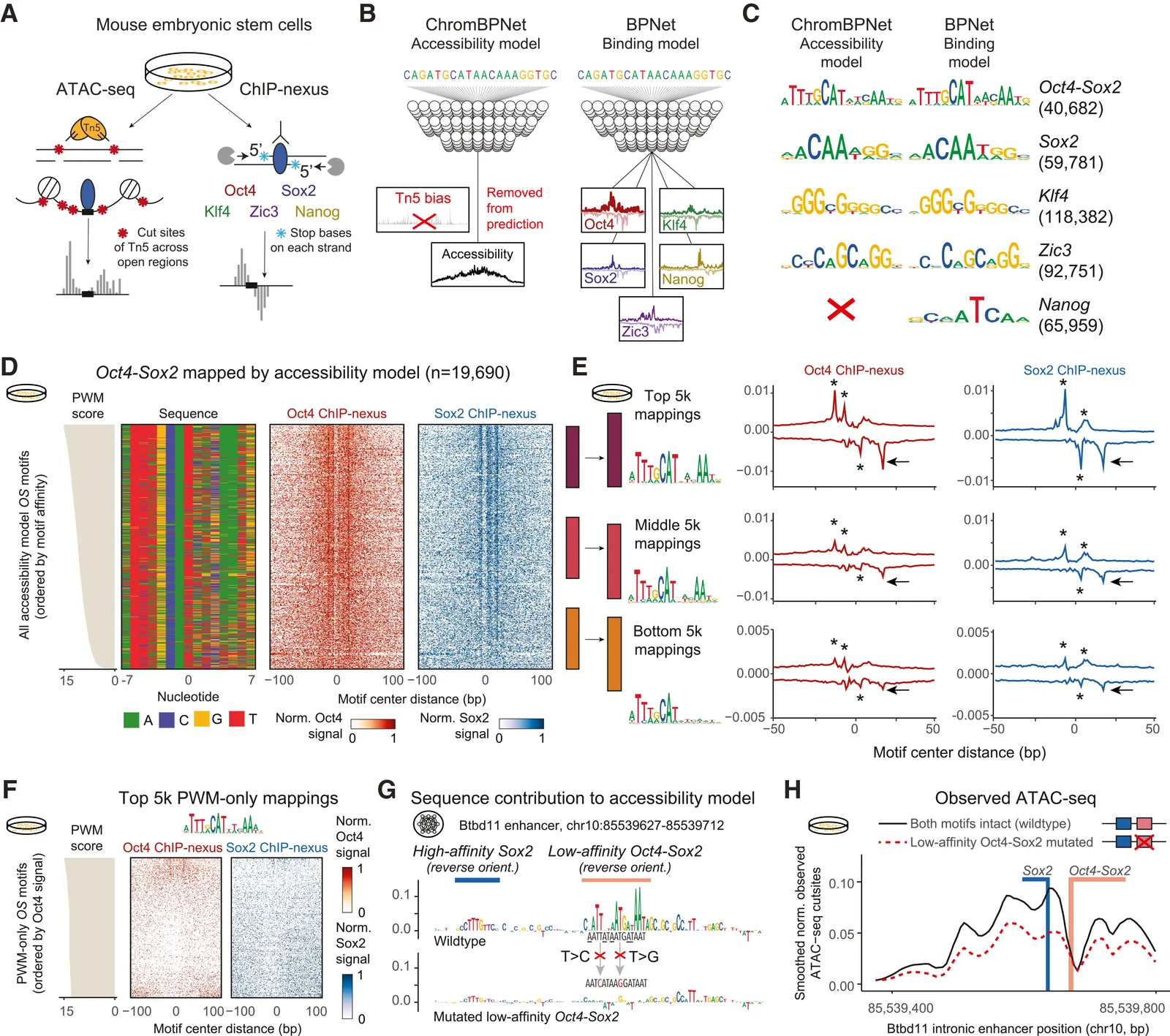
BPNet and ChromBPNet models predict and map functional low-affinity pioneer motifs (A) Graphic depicting ATAC-seq and ChIP-nexus experiments performed on R1 mouse embryonic stem cells. (B) Graphic depicting sequence-to-profile modeling with ChromBPNet and BPNet. (C) The accessibility (ChromBPNet) and binding (BPNet) models returned expected CWMs of motifs, apart from *Nanog*, which was not found by the accessibility model. (D) *Oct4-Sox2* motifs mapped by the accessibility model, ordered by PWM scores (left, beige), shown as sequence colormap (second panel), with Oct4 (third panel, red), and Sox2 (fourth panel, blue) binding footprints shown in normalized heatmaps. (E) The top, middle, and bottom 5,000 *Oct4-Sox2* motifs based on the PWM scores are shown with their PWM logos (left) alongside the average read-per-million (RPM)-normalized ChIP-nexus profiles of Oct4 (red) and Sox2 (blue). Stars mark the ChIP-nexus footprints demarcating the protein-DNA contacts. The black arrow marks the outer right ChIP-nexus footprint of Sox2 and Oct4, which persists even at the bottom 5,000 *Oct4-Sox2* motifs when the *Sox2* component is no longer clearly recognizable. (F) Normalized Oct4 (red) and Sox2 (blue) binding at the top 5,000 *Oct4-Sox2* motifs mapped only by PWM scanning in the same regions, ordered by PWM scores (left, beige). (G) The intronic *Btbd11* enhancer shown with contribution scores from the ChromBPNet accessibility model across the wild-type sequence (top) and for the sequence where, near a high-affinity *Sox2* motif (AACAAAGGC, oriented on the reverse strand), a low-affinity *Oct4-Sox2* motif was mutated by two base substitutions to abolish its accessibility contribution (bottom). Note that the low-affinity *Oct4-Sox2* motif (AATTATAATGATAAT) is the reverse complement of ATTATCATTATAATT, which is the orientation of the motif introduced above and which has five mismatches (underlined) to the inferred *Oct4-Sox2* motif consensus ATTTGCATAACAATG, two in the motif’s core ATTTGCATAACAATG. (H) Experimentally observed ATAC-seq profiles across the *Btbd11* enhancer for the wild type (solid line) and the CRISPR mutated sequence (dotted line).

Both models learned similar *Oct4-Sox2*, *Sox2*, *Klf4*, and *Zic3* motifs (Figures 1C, S1B, and S1C), consistent with experimental findings that Oct4, Sox2, and Klf4 are pioneer TFs in mESCs.^55^^,^^56^^,^^57^ The *Nanog* motif was not learned by the accessibility model (Figure 1C), suggesting that Nanog is not a pioneer TF, consistent with Nanog’s dependency on other TFs for binding^36^ and the relative lack of observed chromatin accessibility effects upon its degradation.^58^

We next mapped all motif instances within the 1-kb genomic regions using CWM scanning,^36^ seeking to map as many contributing low-affinity motifs as possible while maintaining confidence that they are specific for the assigned TF (STAR Methods). The returned motif mappings go below typical PWM consensus matches (<85%)^59^^,^^60^ and thus possess a broader distribution of sequence affinities than those from traditional PWM scanning (Figures 1D and S1D; STAR Methods) but are bound in *in vitro* protein-binding experiments^61^^,^^62^^,^^63^^,^^64^ (Figure S1E). For simplicity, we refer to an imperfect motif match as “low-affinity,”^2^ regardless of its *in vitro* binding affinity.

To validate that this approach correctly mapped functional motifs, we chose the composite *Oct4-Sox2* motif as an example because longer motifs show a larger number of mismatches to the consensus sequence while still being functional.^35^^,^^65^ Key pioneer motifs mapped by the accessibility model consistently showed sharp ChIP-nexus footprints by the corresponding TF, although these binding data were never seen by the accessibility model (Figures 1D, 1E, and S1G). Interestingly, the weaker *Oct4-Sox2* motifs showed ChIP-nexus footprints for both Oct4 and Sox2, but the *Sox2* motif component was often non-existent, consistent with previous observations of strong pioneering effects at regions with an *Oct4* motif in mESCs.^22^^,^^66^ Thus, the vast majority of mapped motifs, including those with lower PWM scores, showed independent experimental evidence for being functional.

As a comparison, we mapped motifs in the same genomic regions using traditional PWM scanning^59^^,^^60^ (STAR Methods). A comparable match score requirement yielded many more mapped motifs, most of which were not mapped with our method (Figure S1H). Almost no specific binding was observed even among the top 5,000 motifs with the highest PWM scores if they were not also mapped with our approach (Figure 1F), showcasing that PWM scanning produces false positives even at high match scores and reiterating that deep-learning models more accurately map functional motifs.^35^^,^^36^ We note that, for functional motif instances, PWM scores still serve as a robust independent metric to measure motif strength (Figure S1E; STAR Methods).

To test whether low-affinity motifs contribute to chromatin accessibility, CRISPR-Cas9-mediated genome-editing experiments were performed at an intronic *Btbd11* enhancer.^36^ Next to a high-affinity *Sox2* motif, a low-affinity *Oct4-Sox2* pioneer motif with key mismatches across both the *Oct4* and *Sox2* components (Figure 1G) was edited by two point mutations predicted to abolish the motif’s pioneering effect (Figures 1G and S1I, STAR Methods). Assay for transposase-accessible chromatin using sequencing (ATAC-seq) experiments on wild-type and mutated cells (Figures S1J and S1K) differed slightly from the predicted effect (Figures S1L) but revealed significantly decreased accessibility around the mutated low-affinity *Oct4-Sox2* motif (Figures 1H and S1M). Taken together, the results suggest that we accurately mapped low-affinity motifs that contribute to chromatin accessibility.

### Low-affinity pioneer motifs are contextually more enhanced

We next analyzed how much each motif contributes to chromatin accessibility through its intrinsic binding affinity to the TF versus effects from the surrounding genomic context. From our accessibility (ChromBPNet) and binding (BPNet) models, we extracted two measurements, isolation scores and context scores, which we use throughout this work.

Isolation scores (also called marginalization scores^21^^,^^67^ or global importance scores^68^) measure the predicted effect of each motif sequence *in isolation* by injecting a motif sequence into randomized sequences and measuring the average log-fold-change over the uninjected control (Figure 2A; STAR Methods). For a TF binding model, predicted isolation scores for mapped motifs can proxy for relative motif affinities.^67^ In contrast, context scores, a motif-centric expansion of *in silico* mutagenesis^69^^,^^70^ or the necessity test,^71^ measure the predicted log-fold-change loss when a motif is mutated in its genomic sequence context^36^ (Figure 2B; STAR Methods).

**Figure 2:**
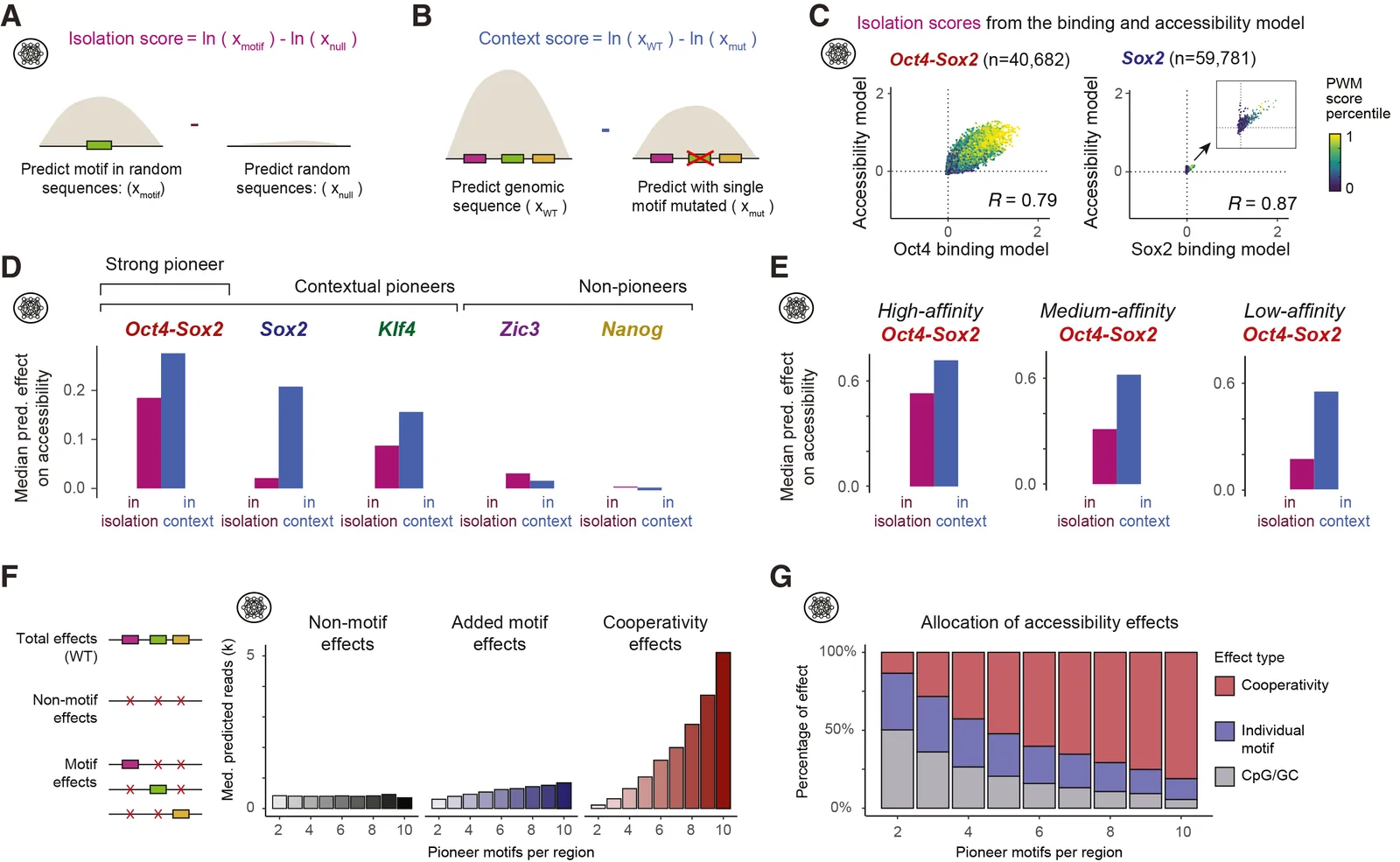
Low-affinity motifs can act as contextual pioneers (A and B) Graphic depicting the calculation of isolation score (A) and context score (B), described further in STAR Methods. (C) Comparison of the isolation scores obtained from the binding model (*x* axis) and the accessibility model (*y* axis) for the *Oct4-Sox2* (left) or the *Sox2* (right) motifs, colored by their PWM score percentiles. (D) Median isolation score for each motif (left) shows its relative pioneering strength, while the median context scores (right) show the motif’s impact in the genomic context. (E) For *Oct4-Sox2* motifs mapped by only accessibility models, median isolation and context scores predicted by the accessibility model of high-, medium-, and low-affinity motifs (5,000 each, based on PWM score) show that low-affinity motifs receive a bigger boost in context than high-affinity motifs. (F and G) Median predicted accessibility levels (F) and their percentage allocation stratified by number of pioneer motifs per region (at least two as specified by Figure S1C) (G) were calculated for non-motif effects (black), total motif effects (blue), and cooperativity effects (red), described further in STAR Methods.

Intuitively, a motif’s isolation and context score should be similar if genomic context does not influence a motif’s effect. If, instead, context scores are far greater, this suggests that motifs are highly contextual and may function cooperatively with other motifs. Thus, this approach directly represents a motif’s entire effect within a genomic context, without having to perform a more focused cooperativity analysis for each motif pair, which we will conduct later.

We confirmed that the binding isolation scores correlate with motif affinities, as previously observed,^21^ and found that the accessibility isolation scores also correlated with PWM scores (Figures 2C and S2A) and *in vitro* protein-binding microarray experiments^61^^,^^62^^,^^63^^,^^64^ (Figure S1F; Table S4; STAR Methods). Indeed, the isolation scores from both models were highly correlated (Figures 2C and S2A; Table S4; Spearman correlation 0.67–0.87) despite these models being trained independently on different types of experimental data and having only moderately correlated context scores (Figure S2B; Table S4). This suggests that relative motif affinities are indeed represented by the isolation scores and they were learned independently by both models.

We next asked how a motif’s intrinsic pioneering strength (isolation score) differed from its genomic effect (context score). To include non-pioneer TFs, we used motifs learned by either model. *Oct4-Sox2* motifs possessed the strongest pioneering effects in isolation, with a relatively small boost in context (Figure 2D). This is consistent with *Oct4-*Sox2’s inherent cooperativity from Oct4 and Sox2 binding, the motif’s strong influence on the binding of other TFs,^36^^,^^72^ and the strong loss of accessibility upon Oct4 depletion.^58^^,^^67^^,^^73^ Conversely, *Zic3* and *Nanog* motifs produced poor correlations between binding and accessibility effects (Spearman correlation, 0.21–0.30; Table S4), suggesting that they do not pioneer chromatin in these cells. Interestingly, for both the accessibility model and binding model, *Sox2* and *Klf4* motifs had weak isolation scores but strong context scores, suggesting that these motifs benefit more from the surrounding genomic context.

The strong contextual effects of *Sox2* and *Klf4* raise the question of whether these are particularly cooperative pioneer TFs or whether weak pioneer motifs in general tend to get bigger contextual boosts. If the latter is true, low-affinity motifs should get boosted proportionally more in their genomic context than high-affinity counterparts. We therefore analyzed the set of *Oct4-Sox2* motifs of high, medium, and low affinity from the accessibility model as described earlier (Figure 2E). We found that high-affinity motifs had, on average, a ∼35% greater effect in context than in isolation, while low-affinity motifs had a ∼300% (3-fold) greater effect. Likewise, *Sox2* and *Klf4* motifs showed lower isolation scores with lower affinities, but context scores decreased less, showing their increased reliance on context for their effects (Figures S2C and 2D). These results suggest a general tendency of motifs with weaker effects to be more enhanced in a genomic context, consistent with low-affinity motif mutations being able to cause strong phenotypic effects.^5^^,^^6^^,^^7^

To understand how the genomic context boosts motif effects, we generated sequence designs that isolate non-motif effects, motif effects, and cooperativity effects (Figure 2F; STAR Methods). By deleting all motifs within each region, we found that some portion of accessibility is explained by non-motif enhancer sequences such as CpG and GC contents, consistent with prior observations.^74^^,^^75^ Similar total effect sizes were observed when the effects of each individual pioneer motif (equivalent to isolation scores) were added up per region, with linearly increasing effects for each additional motif. The remaining effects come from the cooperative interactions between motifs. These cooperative interactions accounted for the majority of effects, with steeply increasing contributions with each additional motif per region (Figures S1L, 2F, and 2G). Thus, cooperative effects largely explain the difference between a motif’s isolation score and its context score.

### Identical motif sequences have different effects depending on adjacent pioneer motifs

To better understand the cooperative motif effects, we tested whether motifs are enhanced by being near other motifs or by being more centrally located within the accessible region, as previously suggested.^19^^,^^21^^,^^27^^,^^37^^,^^38^^,^^76^ We used accessibility context scores for a motif’s predicted pioneering strength and asked how these scores are determined by motif proximity, centrality within the ATAC-seq region, or motif affinity itself (Figure 3A). High pioneering effects of weaker pioneering motifs were best predicted by small distances to the strongest pioneer motif, the motif with the highest accessibility context score in the region (Spearman correlation, 0.37–0.38). The pioneering effects explained by distance were even higher than motif affinity itself (Spearman correlation, 0.06–0.11) (Figure 3B; Table S5).

**Figure 3:**
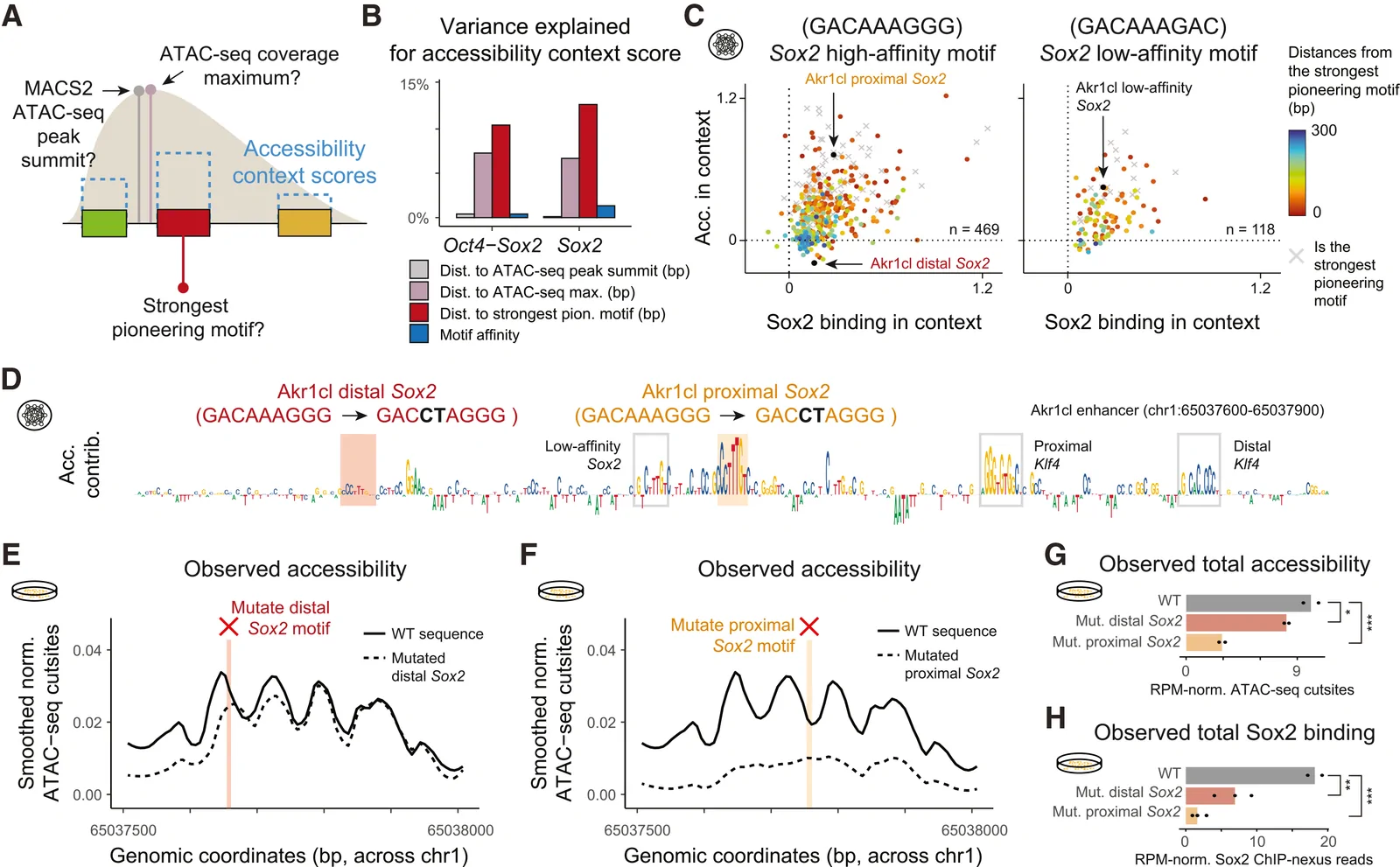
Distance-dependent arrangements of pioneer motifs drive strong effects (A) Graphic depicting different measurements across every mapped motif as follows: (1) the distance from the center of the considered motif to the center of the strongest pioneer motif (defined as possessing the highest accessibility context score), (2) the distance from the MACS2 accessibility peak summit, and (3) the distance from the ATAC-seq fragment coverage maximum. (B) Bar plots of variance explained from each measurement to explain *Oct4-Sox2* and *Sox2* accessibility contribution scores using independent linear regression. Strongest pioneering motifs were excluded. (C) Binding and accessibility context scores of mapped *Sox2* motifs, which contain precise sequence matches to a high-affinity *Sox2* (GACAAAGGG) or a low-affinity *Sox2* (GACAAAGAC), colored by the distance of each *Sox2* to the strongest pioneer motif in the region. Gray crosses indicate the strongest pioneer motifs. (D) Accessibility contribution scores of the *Akr1cl* upstream enhancer (chr1: 65037600–65037900) with contributing motifs marked and the two sequence substitutions of the distal and proximal *Sox2* sequences annotated. (E and F) Smoothed experimental wild type (solid line) and CRISPR mutant (dashed line) ATAC-seq cut-site profiles across the *Akr1cl* enhancer compare the effects of (E) mutating the distal *Sox2* motif and (F) mutating the proximal *Sox2* motif. (G and H) RPM-normalized median (colored bars) total (G) ATAC-seq cut sites or (H) Sox2 ChIP-nexus reads across replicates (black dots) occurring across chr1:65037600–65037900 for wild type (black), distal *Sox2* mutant (red), and proximal *Sox2* mutant (orange) experiments. Significance was tested via a one-tailed *t* test between replicate experiments (black dots).

This suggests that the same motif may mediate substantially stronger pioneering effects when closer to a strong pioneer motif. For example, when considering all motifs of the same sequence, say a high-affinity *Sox2* (GACAAAGGG) or a low-affinity *Sox2* (GACAAAGAC), the motif’s context scores for accessibility and TF binding are both enhanced with closer distances (Figure 3C; Table S5).

To experimentally test whether identical motif sequences have different effects on chromatin accessibility, we performed CRISPR-Cas9 editing on the *Akr1cl* enhancer. This region has two high-affinity *Sox2* motifs of the exact same sequence but with different accessibility context scores. Both are bound by Sox2 (Figures S3A and S3B), but one is located proximally to a low-affinity *Sox2* motif and two *Klf4* motifs, while the other is positioned more distally (Figure 3D). After selecting successful CRISPR-Cas9 clones with targeted point mutations in each motif, we performed ATAC-seq and Sox2 ChIP-nexus experiments to confirm the loss of Sox2 binding (Figures S3C–S3E).

Mutating the distal *Sox2* motif had a slight yet significant decrease in accessibility levels (Figures 3E and 3G), but mutating the proximal *Sox2* motif produced over a 3-fold reduction in accessibility (Figures 3F and 3G). These results differed slightly from the predicted effects (Figure S3F) but confirmed that the proximal *Sox2* motif mutation reduced accessibility more strongly than the distal one.

We also investigated whether the differential effect on accessibility affected Sox2 binding. Sox2 was still bound to the intact proximal motif when the distal *Sox2* motif was mutated (Figure S3G). However, when the proximal *Sox2* motif was mutated and chromatin accessibility was strongly reduced, the intact distal *Sox2* motif was no longer bound by Sox2 (Figure 3H and S3H). This suggests a hierarchy of TF binding where binding to strong pioneer motifs promotes binding to motifs with weaker pioneering effects in the same region.

### Pioneering cooperativity occurs through nucleosome-range soft syntax

Thus far, motif cooperative effects were observed to steeply increase with the number of motifs and to depend on motif proximity. This is consistent with pairwise interactions between motifs, which grow quadratically with the number of motifs per region. We therefore systematically queried the model to identify the rules by which two motifs cooperate. For each genomic region with a motif pair, we mutated one motif alone or both together and tested whether the joint effects (wild type over the double mutant) are more than the sum of each motif’s effect (each single mutant over the double mutant)^19^^,^^37^^,^^77^ (Figure 4A).

**Figure 4:**
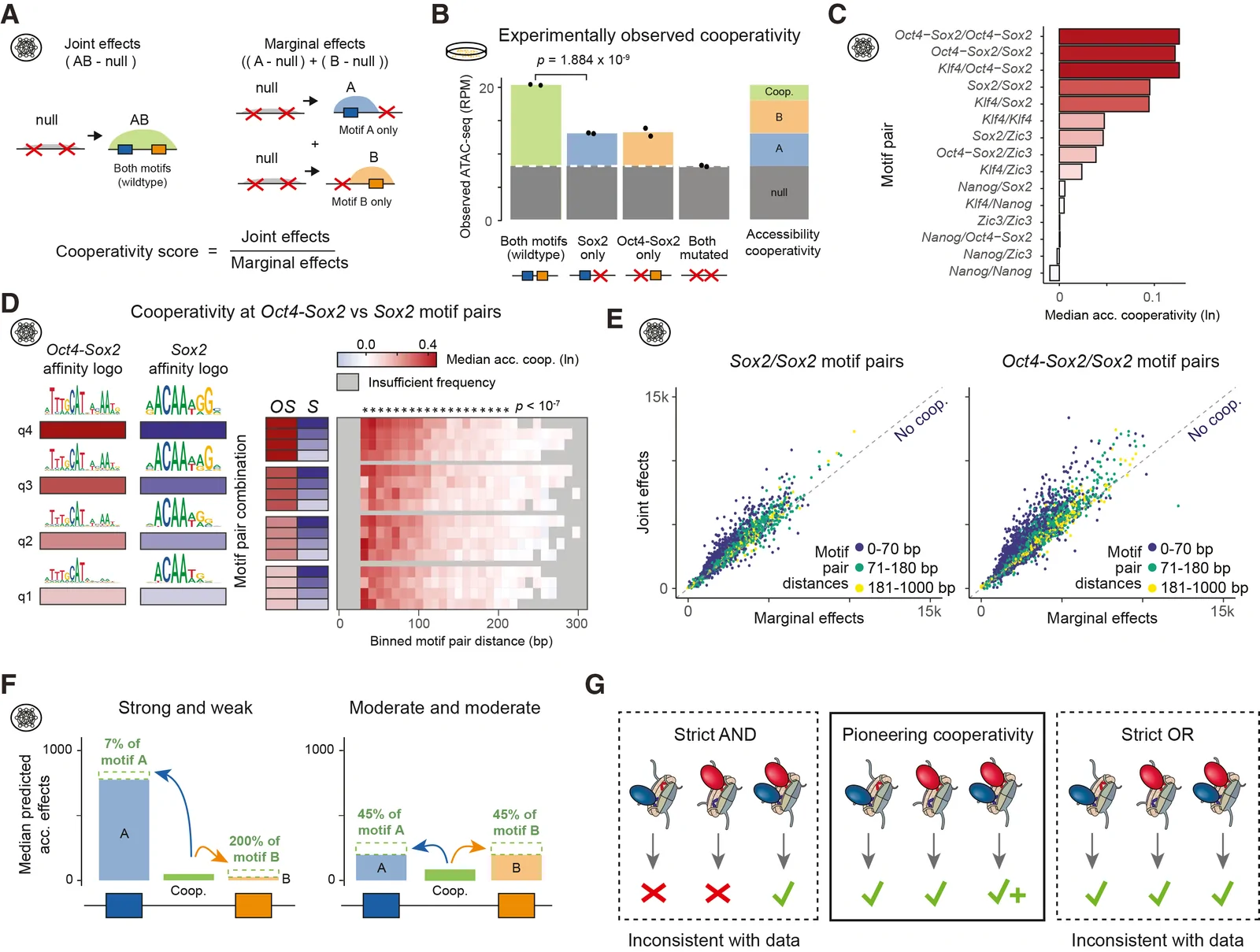
High-affinity and low-affinity motifs cooperate within nucleosome distances to increase accessibility (A) Graphic defining superadditive accessibility cooperativity. (B) RPM-normalized ATAC-seq reads across the *Btbd11* intronic enhancer (chr10:85539400–85539800) from experiments conducted on CRISPR-edited cell lines of the four motif-pair configurations when mutating the high-affinity *Sox2* and low-affinity *Oct4-Sox2* motif. Joint effects and marginal effects of each motif were calculated across each replicate set, with significance derived from a one-tailed *t* test. To the right, calculated cooperative effects (green) are stacked upon individual observed effects. (C) Median accessibility cooperativity (also colored) across all motif pairs of key mESC motifs mapped by both binding and accessibility models occurring within 300 bp of one another. (D) Median accessibility cooperativity between *Oct4-Sox2*/*Sox2* motif pairs across quartiles of motif affinity and motif-pair distances. Motif affinity and binned distance configurations with fewer than 20 motif pairs were not considered (gray boxes). Cooperativity significance was derived from a one-tailed Wilcoxon test comparing whether *Oct4-Sox2*/*Sox2* motif pairs arranged at select binned distances were more cooperative than *Oct4-Sox2*/*Sox2* motif pairs arranged at very long distances (>400 bp) with multiple-comparison Bonferroni corrections and an adjusted significance cutoff of *p* < 10^−7^. (E) Comparison of joint and marginal predicted accessibility effects for the *Sox2*/*Sox2* (left) and *Oct4-Sox2*/*Sox2* (right) motif pairs, colored by the pairs’ relative center-to-center distances to one another. Motif pairs with higher joint effects than marginal effects were interpreted as cooperative. (F) As denoted in (A), median accessibility marginal effects (blue and orange) and calculated cooperativity levels (green) of all pioneer motif pairs containing *Oct4-Sox2*, *Sox2*, and *Klf4* arranged within 200 bp of one another. The left panel depicts motifs with the strongest 20% predicted accessibility context scores paired with motifs with the weakest, non-zero 20% predicted context scores. The right panel depicts paired motifs both within the middle 20% of accessibility context scores. (G) Implications for the mechanisms of nucleosome-mediated cooperativity.

We confirmed that this cooperativity calculation was consistent with our results from CRISPR editing at the *Btbd11* enhancer. We mutated the low-affinity *Oct4-Sox2* motif, the high-affinity *Sox2* motif, or both motifs and measured the changes in ATAC-seq accessibility on validated clones (Figures S1J and S1K). While the observed effects differed slightly from the predicted effects (Figure S1L), the joint effect of both motifs was larger than the added effects of each motif (Figures 4B and S1L), and the cooperative effect was of the expected magnitude for an accessible region containing only two pioneer motifs (Figures 2F and 2G).

We then analyzed the cooperativity between our five pluripotency TFs. All pairs of pioneer motifs showed cooperativity substantially above 1 (or above 0 in log-space) (Figures 4C, S4A, and S4B; Table S6), while the cooperativity was weakened or non-existent when one motif was a non-pioneer motif, such as *Nanog* (Figures 4C, S4A, and S4C). This was true across all accessibility models; thus, it was robust to the training region selection. Furthermore, similar syntax was found for the additional cross-validated motifs beyond those bound by the five pluripotency TFs (Figures S4D and S1C). Even CTCF was subject to cooperative boosts from pioneer motifs (Figure S4E), consistent with experimental evidence.^78^ This suggests that cooperativity is very common and perhaps the default among pioneer TFs.

Moreover, the distance-dependent enhancements were similar between different motif pairs (Figures 4D, S4A, and S4D) and across various motif affinities (Figures 4E, S4B, and S4E). Significant cooperativity scores (above those of very distant motif pairs) typically occurred within distances of 200 bp and were highest for motif distances of <70 bp (Figures 4D, 4E, S4A, and S4B), thus a soft syntax that evokes a nucleosome-mediated mechanism.^36^

These rules of cooperativity are consistent with our initial observation that weak pioneer motifs tend to have strong effects in their genomic context. When weak motifs cooperate with stronger motifs, the resulting accessibility increase is higher for the weak motif than the strong motif, while two motifs of similar strength benefit from cooperating more equally (Figure 4F). Low-affinity motifs are also inherently enhanced because they are only functionally relevant (thus mapped by our models) when they cooperate with stronger pioneer motifs, while strong pioneer motifs may sometimes function on their own (Figure 4F).

Since the strongest effects occurred between two nearby pioneer motifs, we considered the mechanistic implications (Figure 4G). There is accumulating evidence for nucleosome-mediated cooperativity,^79^^,^^80^^,^^81^^,^^82^ but the exact mechanisms are not understood. The involvement of two pioneer TFs suggests that their relationship is not simply a hierarchical one where one pioneer TF enables the binding of another TF to accessible DNA.^80^^,^^81^ Instead, the two pioneer TFs likely cooperate in removing the same nucleosome.

Our analyses rule out two extreme models for this cooperativity. First, we can exclude a model where two or more pioneer TFs must be present together on the same nucleosome to remove it (“Strict AND” in Figure 4G). Such a strict requirement for multiple TFs is inconsistent with the model’s prediction that each pioneer TF produces a certain amount of accessibility per motif. Second, we can exclude a model where each pioneer TF independently removes the same nucleosome with a certain probability, causing increased accessibility across the cell population (“Strict OR” in Figure 4G). If this were the case, this would produce less than additive effects (Figure S4F). This suggests that two pioneer TFs quantitatively enhance each other’s effects in removing a nucleosome and that this mechanism applies to low-affinity motifs.

### Modeling suggests that pioneering cooperativity changes the maximum regulatory potential

We next explored a kinetic modeling framework to understand the implications of nucleosome-mediated cooperativity, especially in response to changing TF concentrations. Based on thermodynamics, increasing TF concentrations lead to increased binding of the TF to its motif, which is roughly linearly at intermediate TF levels and then plateauing toward 100% at high concentrations.^10^^,^^83^^,^^84^ For low-affinity motifs, higher TF concentrations are required to reach full TF occupancy.^17^ How motifs and their cooperative effects modulate chromatin accessibility as a function of TF concentration is not known.

Our approach is similar to previous thermodynamic equilibrium models in that it considers TF binding to a closed and an open conformation for which the TF has different affinities^27^^,^^79^^,^^81^ (Figure 5A). It can be parameterized to be either at thermodynamic equilibrium or away from equilibrium to account for the irreversible nature of nucleosome removal by ATP-dependent remodelers^85^^,^^86^ (Figures S5A and S5B). In this kinetic model, the strength of pioneering at a given TF concentration depends on the motif’s affinity (1/*g*) over non-specific binding, the nucleosome intrinsic affinity for DNA (*k*_*close*_/*k*_*open*_), the nucleosome’s effect on destabilizing TF binding (*β*), and the TF’s ability to increase the rate of opening through nucleosome remodeling (*o*) and possibly preventing nucleosome reformation (1/*c*) (Figures 5A and S5C; STAR Methods).

**Figure 5:**
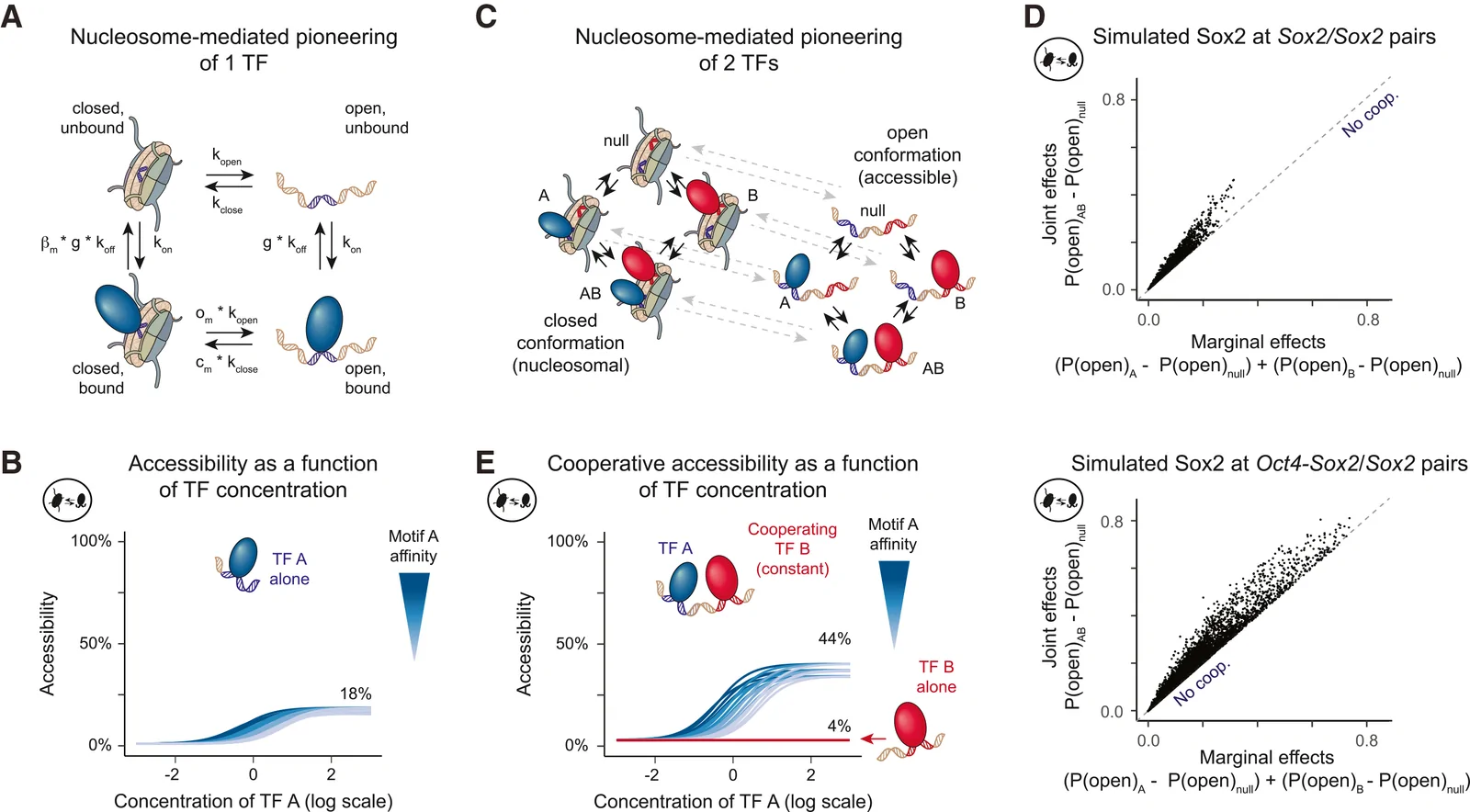
Pioneering cooperativity is predicted to change the TF dose-response curve (A) Graphic depicting a kinetic model out of equilibrium of bound TF effects on nucleosome removal and reformation. Parameter definitions are as follows: affinity (1/*g*, with *g* < 1 the factor decrease in unbinding rate on the motif relative to non-motif DNA sequence); TF apparent association rate (*k*_*on*_), linearly related to TF concentration; TF dissociation rate (*k*_*off*_), chromatin opening rate in absence of bound TF (*k*_*open*_); chromatin closing rate in absence of bound TF (*k*_*close*_); nucleosomal effect upon TF dissociation rate (*β*); TF effect on nucleosome removal (*o*); TF effect on nucleosome reformation (*c*). Subindex _m_ corresponds to the site being a motif (see Figure S5B). (B) 1 TF kinetic model simulations (*n* = 5) measuring the accessibility state, measured as the steady-state probability of the open conformation, upon pioneer TF Sox2 binding over a range of motif affinities across changing concentrations in units of 10∗nM. Parameter ranges are in Table S8. (C) Graphic depicting an expansion of the kinetic model shown in (A), allowing for simultaneous effects of two pioneer TFs. (D) 2 TF kinetic model simulations (*n* = 50,000) comparing joint and marginal accessibility states, measured as steady-state probability of the open conformation, for Sox2 binding at a *Sox2/Sox2* motif pair (top) and an *Oct4-Sox2/Sox2* motif pair (bottom). Parameter ranges are in Table S9. Simulations that returned higher joint effects than marginal effects were interpreted as cooperative. (E) 2 TF kinetic model simulations (*n* = 5) measuring the accessibility state, measured as steady-state probability of the open conformation, upon two pioneer TFs binding over a range of TF A motif affinities across changing concentrations of TF A in units of 10∗nM. TF A was modeled from Sox2 binding dynamics *in vivo* with the same parameters as (B). TF B was modeled with the same pioneering parameters but with static motif affinity and TF concentration, such that its unchanging effect on accessibility in the corresponding 1 TF kinetic model is depicted with a red line. Although TF B affinity and concentration was kept constant, the presence of TF B caused accessibility states to be cooperatively enhanced beyond those of TF A alone (B). Parameter ranges are in Table S9.

We applied this kinetic model to Sox2 since we could choose parameter ranges in agreement with measured Sox2 concentrations in mESCs^87^ and Sox2 binding dynamics *in vitro* and *in vivo*.^88^^,^^89^^,^^90^ By modeling a single *Sox2* motif and choosing diverse parameter sets representing variability of individual genomic regions within plausible ranges (STAR Methods), we observed Sox2 pioneering strength proportional to motif affinity (Figure S5D), as observed in the accessibility deep-learning models (Figure 2C).

We then explored how chromatin accessibility depends on motif affinity and Sox2 concentration. With rising Sox2 concentrations, accessibility levels increased almost linearly. Lower motif affinities required higher Sox2 concentrations to achieve the same effect, but, once the Sox2 concentration reached sufficient levels such that both the high-affinity and low-affinity motifs were maximally occupied, the curves plateaued at the same maximum accessibility (Figure 5B). Thus, even with 100% Sox2 binding occupancy, there could be a limit to the maximum accessibility that can be achieved by a single TF, and this maximum depends on the ability of the TF to promote the open conformation and the nucleosome’s affinity for DNA (Figures 5B and S5C). This is consistent with single-molecule footprinting studies *in vivo* showing that typically only a fraction of nucleosomes are removed at accessible regions, even when multiple pioneer motifs are present.^80^^,^^81^^,^^91^^,^^92^ Thus, our modeling suggests that each pioneer motif likely has a maximum effect, which we term regulatory potential.

To study pioneer cooperativity, we expanded the kinetic model to simultaneously consider two TFs binding to their corresponding motifs (Figure 5C; STAR Methods). We assumed that, when both TFs are bound (“AB” in Figure 5C), the factors by which each TF increases the opening rate (*o*_*m*_) multiply, which can be interpreted to correspond to an additive effect on lowering the activation energy of the transition (STAR Methods). Under this assumption, we could readily simulate the cooperativity effects predicted by our accessibility deep-learning model of Sox2 binding to *Sox2*/*Sox2* and *Oct4-Sox2*/*Sox2* motif pairs (Figure 4E) over a range of biologically plausible motif affinities and parameter settings (Figure 5D; STAR Methods).

We then asked how pioneering cooperativity affects the response curve and regulatory potential (Figures 5E and S5E). We again simulated Sox2 binding (TF A on motif A), but now with a cooperating motif B, whose pioneer TF B was kept at a constant low concentration (STAR Methods). With rising TF A concentrations, the total fraction of accessible chromatin increased more rapidly (Figures 5E and S5E) and reached a higher plateau compared to a single motif (Figure 5B). Thus, putting a motif into a cooperative context confers distinct properties on chromatin opening. While both motif affinity and motif cooperativity increase the chromatin accessibility at lower TF concentrations, only motif cooperativity boosts the accessibility beyond what a single TF can achieve at saturating TF concentrations when it fully occupies nucleosomal and naked DNA (Figures 5E, S5F, and S5G). This suggests that pioneering cooperativity extends the regulatory potential of the motif.

As expected, the cooperative influence of TF B on the effect of TF A was dependent on both the affinity and concentration of TF B. With the concentration of TF B being constant, switching motif B to low affinity lowered the regulatory potential of motif A (Figure S5H). Conversely, increasing the concentration of TF B together with that of TF A enhanced the regulatory potential, maximizing the dynamic range of the response of TF A. We note that, given the parameter values chosen, most of our simulations still do not reach 100% accessibility, consistent with regulatory potential playing a role under physiological conditions.

Overall, these results predict that the response to changing TF concentrations during embryonic development not only depends on motif affinity but also on motif cooperativity. In fact, low-affinity motifs have relatively weak effects and do not cooperate more strongly than high-affinity motifs (Figures 4D and S5G), and thus cooperativity of high-affinity motifs is predicted to have an outsized effect on the response to changing TF concentrations.

### Cooperating motifs are more sensitive to changing Oct4 concentrations *in vivo*

We tested the modeling predictions *in vivo* by analyzing an accessibility time-course dataset in mESCs where the concentration of Oct4 gradually decreases after doxycycline withdrawal (0, 3, 6, 9, 12, and 15 h)^66^ (Figure S6A). To test whether loss of Oct4 was the main driver for the observed accessibility changes, we trained separate models for each time point and analyzed the motifs as before (Figure 6A; Table S2). Contributions of only *Oct4-Sox2* motifs decreased over time (Figure 6B). The *Oct4-Sox2* motif was no longer discovered by 15h, when Oct4 was at 2.5% and the *Oct4-Sox2* motif contributions were minimal (Figure S6B). These results confirm the robustness of model training and interpretation (Figure S6C) and suggest that decreasing contribution of the *Oct4-Sox2* motif is the main driver for the observed accessibility changes.

**Figure 6:**
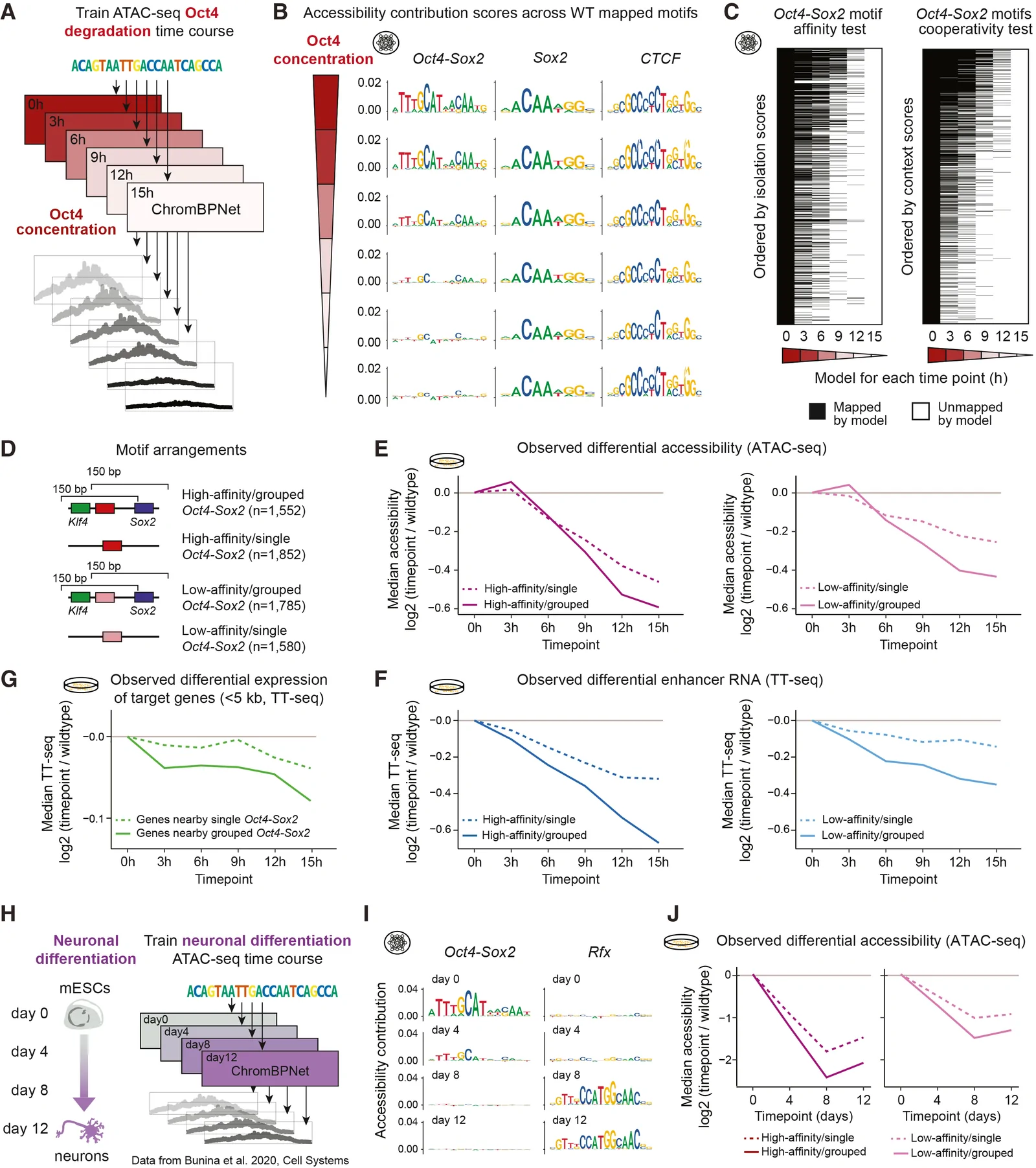
Cooperative motif arrangements bolster motif pioneering across changing TF concentrations (A) ChromBPNet accessibility models trained on ATAC-seq data collected over decreasing Oct4 concentrations via doxycycline withdrawal experiments. (B) Accessibility CWMs measured across all Oct4 concentration time points using mapped motifs derived from the R1 mESC accessibility model (Figures 1B, 1C, and S1C) for *Oct4-Sox2*, *Sox2*, and *Ctcf* motifs. (C) Heatmaps marking which *Oct4-Sox2* motifs mapped by the 0-h time-point model were mapped by the corresponding time-course accessibility models, ordered by motif isolation scores as proxy for motif affinity (left) or context scores (right). Both sets of scores were from the 0-h time-point model. (D) Graphic denoting arrangements of single/grouped and strong/weak *Oct4-Sox2* motifs. *Oct4-Sox2* motifs were derived from the 0-h time-point model, while high- and low-affinity motifs were classified as the top and bottom tertiles of measured PWM scores. *Oct4-Sox2* motifs were considered grouped if they were within 150 bp of either a *Sox2* or *Klf4* motif. Only motif arrangements with total accessibility >60^th^ percentile at 0 h were considered. (E–G) Median experimental differential accessibility (E), TT-seq enhancer expression (F), and TT-seq target gene expression (G) levels measured over the concentration time course across regions with either single (dashed line) or grouped (solid line) *Oct4-Sox2* motifs. For (E and F), subplots depict high (left) and low (right) affinity groups. For (G), target genes were considered if a single *Oct4-Sox2*-containing region was within 100 kb and the nearest target gene was within 5 kb of the nearest *Oct4-Sox2*-containing region. (H) ChromBPNet accessibility models were trained on ATAC-seq data collected over a neuronal-differentiation time course. (I) Accessibility CWMs measured across all neuronal-differentiation time points using mapped motifs derived from the mESC day 0 time point (*Oct4-Sox2*) or the neuronal day 12 time point (*Rfx*). (J) Median experimental differential accessibility levels were measured over the neuronal-differentiation time course across regions with either single (dashed line) or grouped (solid line) *Oct4-Sox2* motifs; subplots depict high (left) and low (right) affinity groups. An arrangement was considered grouped if *Oct4-Sox2* motifs were near any other pioneer found by the accessibility model trained on day 0 (mESC) ATAC-seq experiments.

We next asked what sequence features best predicted when individual *Oct4-Sox2* motifs were no longer detected. Based on thermodynamics, high-affinity motifs should be occupied the longest, thus the *Oct4-Sox2* isolation scores should be a good predictor for the continued motif detection. Our modeling suggested, however, that the loss of accessibility depends on the *Oct4-Sox2* motif in its cooperative context, and thus the context scores should also be a good predictor. Indeed, we found that, while both measurements were predictive, the context scores were the better predictor (Figures 6C and S6D), especially for the *Oct4-Sox2* motifs that were mapped at lower Oct4 concentrations (Figure S6E). This suggests that pioneer motifs are detected at lower TF concentrations if they are contextually enhanced.

We next asked whether motif cooperativity changes the concentration response curve of Oct4. Our kinetic model suggested that cooperative arrangements have higher accessibility potential and thus should start with higher accessibility levels, with a faster loss over time. To test this, we sampled sufficiently accessible regions with exactly one high-affinity, or alternatively one low-affinity, *Oct4-Sox2* motif, which were either found in a grouped cooperative arrangement with *Klf4* and *Sox2* motifs or had a single *Oct4-Sox2* motif (Figures 6D and 6E), while considering confounding variables (Figures S6F–S6H). For each time point, the loss of accessibility compared to the starting point (0 h) was quantified using DESeq2.^93^ Regions with a *Sox2* motif but no *Oct4-Sox2* motif were used as control (Figure S6I).

Grouped *Oct4-Sox2* motifs showed more rapid loss of accessibility over time than single *Oct4-Sox2* motifs (Figure 6E). While high-affinity motifs showed more accessibility loss than low-affinity motifs, both showed bigger effects in the grouped arrangement than when alone. Using all pioneer motifs in the grouped *Oct4-Sox2* motif arrangements had similar effects (Figure S6J). These results corroborate that cooperativity is a critical factor in making an enhancer more sensitive to changing TF concentrations.

Cooperativity-associated rapid loss of chromatin accessibility was also associated with a more rapid drop in enhancer activity and target gene expression, measured as newly transcribed enhancer RNA with transient transcriptome sequencing (TT-seq) over the same Oct4 depletion time course.^66^ Regions with grouped rather than single *Oct4-Sox2* motifs showed a more rapid loss of enhancer transcription and nearby (within <5 kb) target gene expression (Figures 6F and 6G). Control grouped and single *Sox2* motifs not near an *Oct4-Sox2* motif did not show these effects (Figures S6I). Thus, accessibility cooperativity observed for Oct4 over changing TF concentrations also affects enhancer and gene activity, supporting the *in vivo* relevance of this phenomenon.

Finally, we tested whether we could identify the effects of motif cooperativity in a more biological context: the differentiation of mouse embryonic stem cells into neurons^94^ (Figure 6H). We trained ChromBPNet models for ATAC-seq experiments collected along the differentiation time course and found that motifs such as *Rfx4* became more important over time, as one might expect,^95^ while pluripotency *Oct4-Sox2* motifs lost importance (Figure 6I). In this setting, grouped *Oct4-Sox2* arrangements also showed a more rapid loss in accessibility than single *Oct4-Sox2* arrangements (Figure 6J), corroborating that motif cooperativity has an influence on the temporal dynamics of development.

## Discussion

Low-affinity motifs are important during development and can impact the phenotypic outcome of mutations.^6^^,^^7^ However, the mechanisms by which low-affinity motifs regulate enhancer function at the level of chromatin accessibility have remained a gap in knowledge. By learning motifs in an inherently combinatorial manner in their genomic context, we show that deep learning accurately maps low-affinity motifs and that their pioneering effect is strongly dependent on its arrangement with other motifs, more than the motif’s affinity. When a weak pioneer motif cooperates with a strong pioneer motif nearby, it strongly benefits from this cooperativity and produces measurable effects on chromatin accessibility. This cooperativity tends to follow a soft syntax that produces chromatin accessibility enhancements that are distance dependent but occur flexibly when motifs are located together within nucleosome range (∼200 bp).

This has evolutionary implications; i.e., low-affinity motifs cooperate during pioneering with less constrained syntax than classical direct cooperative TF binding, and, thus, low-affinity motifs can more easily evolve *de novo* near an existing pioneer motif. The higher statistical likelihood of low-affinity motifs arising, the flexible distance requirement for cooperativity, and the potential for strong effects make it likely that low-affinity motifs broadly shape enhancer function during evolution.

Such a role in evolution is consistent with—and might even explain—the observation that TF motifs undergo high evolutionary turnover.^96^ With multiple motifs contributing to an accessible region, there are fewer constraints on individual motifs, allowing weaker motifs to evolve and disappear to subtly alter an enhancer’s function. If indeed widespread, this could explain why systematic mutagenesis experiments produce phenotypic changes at sequences outside mapped TF motifs^97^^,^^98^ and why novel regulatory elements often arise near already-accessible regions.^99^^,^^100^

While analyzing low-affinity motifs in their cooperative context, we discovered that pioneering cooperativity has general properties relevant to gene expression. While chromatin accessibility was known to be a rate-limiting step for enhancer function, how exactly each motif influences this process was unclear. When we modeled nucleosome-mediated cooperativity with a kinetic model, we discovered that motif cooperativity extends the intrinsic ceiling of a motif’s effect on chromatin accessibility. This makes intuitive sense since cooperativity produces effects more than the sum of each motif, and, thus, a cooperating motif produces higher maximum accessibility. We termed such maximum impact as the regulatory potential that the TF’s motif has on the enhancer’s function.

The concept of regulatory potential is relevant when the TF’s concentration increases or decreases during development, as we have shown for a time-course experiment of decreasing Oct4 concentrations or neuronal differentiation. A higher regulatory potential due to cooperativity means that the TF’s effect on chromatin accessibility is higher, causing the accessibility to drop more rapidly and linger longer with decreasing TF concentrations. We see this more rapid drop also reflected in the levels of enhancer transcription and target gene expression, consistent with the levels of chromatin accessibility being a parameter that affects gene expression dynamics.

The distance-dependent soft syntax we observed for chromatin accessibility also has mechanistic implications and suggests that pioneering and TF binding are intertwined. When interpreting a BPNet model predicting TF binding in mESCs, we found that the TFs often cooperate through soft syntax in a directional manner.^21^^,^^35^^,^^36^^,^^51^ This suggests that TFs enhance each other’s binding through their effect on chromatin accessibility and that there is a hierarchy by which TF binding occurs *in vivo*. TFs with strong pioneer motifs, i.e., Oct4 and Sox2 to the *Oct4-Sox2* motif, likely bind first, while non-pioneer TFs such as Nanog mostly bind when the chromatin is already accessible. Our CRISPR-editing experiments at the *Akr1cl* enhancer further support and expand upon this model. The *Sox2* motif found in a context where it produces strong pioneering was necessary for Sox2 to bind to the identical *Sox2* motif with weak pioneering effects, but not vice versa. This suggests that motifs in a region are bound in a hierarchical manner, with strong pioneer motifs becoming bound before weaker ones, even when these are identical motif instances of the same TF.

How exactly TFs cooperate in pioneering remains an open question. Our kinetic model of nucleosome-mediated cooperativity readily simulated our data, consistent with TFs having a direct effect on the rate of ATP-dependent nucleosome removal. We note, however, that multiple regulatory steps contributed to the simulated opening effects. For example, our kinetically modeled TFs were set to possess slightly more affinity for DNA in the open versus closed state. This parameter is the main determinant of pioneering strength in classically employed thermodynamic equilibrium models of nucleosome-mediated cooperativity^27^^,^^79^^,^^81^ (Figure S5A). This contradicts the concept of a pioneer TF that exclusively opens chromatin through its ability to bind nucleosomal DNA^18^ but is consistent with the tendency of TFs to bind naked DNA more readily than nucleosomal DNA.^101^^,^^102^^,^^103^ By binding in the open state, a TF could reduce the rate of nucleosome reformation or promote the removal of neighboring nucleosomes.^87^^,^^104^ This could explain the additional accessibility that is observed when enhancers are active^21^ or how non-pioneer TFs might contribute to chromatin accessibility.^105^ How exactly various nucleosome-mediated mechanisms^102^ contribute to making chromatin accessible is an interesting topic for future mechanistic studies.

### Limitations of the study

Interpretation of sequence-to-function deep-learning models to deduce the underlying molecular mechanisms relies on the assumption that these models have been trained on sequences with sufficiently diverse motif affinities and arrangements to correctly learn the sequence rules. Models have been shown to robustly predict motif affinity effects and cooperativity effects,^19^^,^^21^^,^^68^ but there is a risk that predictions of multiple motif perturbations are out of distribution for stable model predictions.^32^ Further, ChromBPNet and BPNet models are designed to predict local regulatory effects of sequences (<3 kb) and are unlikely to learn longer-range impacts of chromatin organization. To confirm the inferred syntactic rules, experimental validations were conducted *in vivo* but were limited to a few individual genomic loci. Moreover, our kinetic modeling is limited to two states (closed/open) with direct transitions not including partially unwrapped conformations or modified nucleosomes.^106^^,^^107^ Finally, we showed that cooperative motif arrangements affect chromatin accessibility, enhancer transcription, and proximal gene transcription, but how distal enhancers regulate transcription remains a complex, unresolved problem.^108^^,^^109^^,^^110^

## Resource availability

### Lead contact

Further information and requests for resources and reagents should be directed to and will be fulfilled by the lead contact, Julia Zeitlinger (jbz@stowers.org).

### Materials availability

This study did not generate new unique reagents.

### Data and code availability

• Raw and supplementary processed data for sequencing experiments have been deposited at GEO: GSE306105 and are publicly available as of the date of publication.
• Trained models supporting intermediate data and motif mappings are available on Zenodo: https://doi.org/10.5281/zenodo.17187407.
• This paper re-analyzes existing, publicly available mESC ZHBTc4 ATAC-seq data, accessible at GEO: GSE174774.
• This paper re-analyzes existing, publicly available ATAC-seq data of mESC differentiation into neurons and is available at ArrayExpress: E-MTAB-8194.
• This paper re-analyzes existing, publicly available human Sox2, mouse Klf1, and human Zic3 protein binding microarray (PBM) data, accessible at UniProbe: UP01612, UP01347, and UP00006, respectively.
• All original code and analysis has been deposited under GitHub: https://github.com/zeitlingerlab/Weilert_mESC_accessibility_2025 and is publicly available.
• Any additional information required to reanalyze the data reported in this paper is available from the lead contact upon request.

## Acknowledgments

We thank Robb Krumlauf, Barak Cohen, Yuning Zhang, Neşet Özel, Sharon Torigoe, and Žiga Avsec for helpful comments and suggestions. We also thank the Stowers Technology Centers for support: Sequencing and Discovery Genomics (Anoja Perera, Michael Peterson, Amanda Lawlor, and Rhonda Egidy), Cytometry (Jose Javier, KyeongMin Bae, and Jeff Haug), Genome Engineering (Kyle Weaver and Victoria Hassebroek), and Tissue Culture (Yan Wang, Sonia Ghosh, Maria Katt, Shilpa Waduwawara, and Chongbei Zhao). Research for this publication has been partially carried out in the Barcelona Collaboratorium for Modelling and Predictive Biology. The research was primarily funded and carried out at the Stowers Institute for Medical Research, with additional funding from the National Institutes of Health (grant 5R01HG010211 to J.Z. and grant F31HD108901 to K.J.B.). Support for R.M.-C. at the Barcelona Collaboratorium for Modelling and Predictive Biology came from the Spanish Ministry of Science, Innovation and Universities MCIU/AEI/10.13039/501100011033 (grant RYC2021-033860-I funded by MICIU/AEI/10.13039/501100011033 and by European Union NextGenerationEU/PRTR; grant PID2022-142210NA-I00 funded by MICIU/AEI/10.13039/501100011033 and by ERDF A Way of Making Europe; Centro de Excelencia Severo Ochoa CEX2020-001049-S) and the Generalitat de Catalunya through the CERCA program.

## Author contributions

M.W. and J.Z. conceived the project with further support from R.M.-C. All data processing, deep-learning modeling, and analysis work was performed by M.W. CRISPR mutations were conceived by M.W. and J.Z., while CRISPR was performed by K.D. Sequencing experiments were generated by K.J.B., K.D., S.K., and H.J. Kinetic models were conceived and designed by R.M.-C. Interpretation of kinetic simulations was done by R.M.-C., J.Z., and M.W. The manuscript was prepared by M.W., R.M.-C., and J.Z. with input from all authors.

## Declaration of interests

J.Z. owns a patent on ChIP-nexus (no. 10287628).

## STAR★Methods

### Key resources table

**Table:** 

REAGENT or RESOURCE	SOURCE	IDENTIFIER
**Antibodies**		

Rabbit polyclonal anti-Sox2	Active Motif	Cat# 3879S; RRID: AB_2255011

**Chemicals, peptides, and recombinant proteins**		

37% formaldehyde solution	VWR	50-00-0
Dynabeads Protein A	ThermoFisher	10008D
phi29 DNA polymerase	New England Biolabs	M0269S
Lambda exonuclease	New England Biolabs	M0262S
Q5 High-Fidelity 2x Master Mix	New England Biolabs	M0492S
dNTP solution mix	New England Biolabs	N0447S
Phenol:chloroform:isoamyl alcohol (25:24:1) (v/v/v)	VWR	136112-00-0
Proteinase K	ThermoFisher	25530049
crRNA and ssODN sequences, see Table S10	IDT	Table S10
Alt-R S.p. Cas9 Nuclease v3	IDT	1081059
Alt-R CRISPR-Cas9 tracrRNA, ATTO 550	IDT	1075928

**Critical commercial assays**		

End Repair Module	New England Biolabs	E6050S
dA-Tailing Module	New England Biolabs	E6053S
Quick Ligation Kit	New England Biolabs	M2200S
Monarch DNA Gel Extraction Kit	New England Biolabs	T1020
Monarch PCR & DNA Cleanup Kit	New England Biolabs	T1030

**Deposited data**		

Raw and analyzed ATAC-seq and ChIP-nexus data	This paper	GEO: GSE306105
Trained deep learning models, supporting intermediate data, and motif mappings (Zenodo)	This paper	Zenodo: https://doi.org/10.5281/zenodo.17187407
Re-analyzed mESC ZHBTc4 ATAC-seq	Xiong et al.^66^	GEO: GSE174774
Re-analyzed ATAC-seq of mESC differentiation into neurons	Bunina et al.^94^	ArrayExpress: E-MTAB-8194
Human Sox2 PBM data	Lai et al.^61^	https://thebrain.bwh.harvard.edu/uniprobe/details46.php?id=1612
Mouse Klf1 PBM data	Mariani et al.^62^	https://thebrain.bwh.harvard.edu/uniprobe/detailsDef.php?id=1347
Human Zic3 PBM data	Badis et al.^63^	https://thebrain.bwh.harvard.edu/uniprobe/details2.php?id=6

**Experimental models: Cell lines**		

R1 mouse embryonic stem cells (ESCs)	Avsec et al.^36^	https://www.nature.com/articles/s41588-021-00782-6
CRISPR ESC line #1: CRISPR-Cas9 of a *Sox2* motif within a *Btb11* enhancer located at chr10:85,539,634-85,539,643	This paper	N/A
CRISPR ESC line #2: CRISPR-Cas9 of a *Oct4-Sox2* motif within a *Btb11* enhancer located at chr10:85,539,666-85,539,681	This paper	N/A
CRISPR ESC line #3: CRISPR-Cas9 of both *Sox2* (chr10:85,539,634-85,539,643) and *Oct4-Sox2* (chr10:85,539,666-85,539,681) motifs within a Btb11 enhancer	This paper	N/A
CRISPR ESC line #4: CRISPR-Cas9 of a Sox2 motif within an *Akr1cl* enhancer located at chr1:65037654-65037663	This paper	N/A
CRISPR ESC line #5: CRISPR-Cas9 of a Sox2 motif within an *Akr1cl* enhancer located at chr1:65037753-65037762	This paper	N/A

**Oligonucleotides**		

Oligonucleotides for ChIP-nexus, see Table S11	IDT	https://research.stowers.org/zeitlingerlab/protocols.html
Illumina Index primer 1: 5′-CAAGCAGAAGACGGCATACGA GAT[i7]GTCTCGTGGGCTCGG-3′	IDT	https://support-docs.illumina.com/SHARE/AdapterSeq/1000000002694_17_illumina_adapter_sequences.pdf
Illumina Index primer 2: 5′-AATGATACGGCGACCACCGAG ATCTACAC[i5]TCGTCGGCAGCGTC-3′	IDT	https://support-docs.illumina.com/SHARE/AdapterSeq/1000000002694_17_illumina_adapter_sequences.pdf
Illumina Transposase adapter read 1 (Nextera A): 5′- TCGTC GGCAGCGTCAGATGTGTATAAGAGACAG-3′	IDT	https://support-docs.illumina.com/SHARE/AdapterSeq/1000000002694_17_illumina_adapter_sequences.pdf
Illumina Transposase adapter read 2 (Nextera B): 5′- GTCTC GTGGGCTCGGAGATGTGTATAAGAGACAG-3′	IDT	https://support-docs.illumina.com/SHARE/AdapterSeq/1000000002694_17_illumina_adapter_sequences.pdf
Mosaic end primer:/5Phos/CTGTCTCTTATACA/3ddC/	IDT	Tn5mC1.1-A1block

**Recombinant DNA**		

pETM11-Sumo3-Tn5 plasmid	Hennig et al.^111^	E54K,L372P
His6-tagged SenP2 protease plasmid	Hennig et al.^111^	N/A

**Software and algorithms**		

CRIS.py	Connelly and Pruett-Miller^112^	https://github.com/patrickc01/CRIS.py
Cutadapt v.4.2	Martin et al.^113^	https://cutadapt.readthedocs.io/en/stable/
Bowtie2 v.2.3.5.1	Langmead and Salzberg^114^	https://bowtie-bio.sourceforge.net/bowtie2/manual.shtml
Picard v.2.23.8	Broad Institute of MIT and Harvard^115^	https://broadinstitute.github.io/picard/
MACS2 v.2.2.7.1	Zhang et al.^116^	https://github.com/macs3-project/MACS
Irreproducible Discovery Rate framework v.2.0.3	Li et al.^117^	https://github.com/nboley/idr
Locally Estimated Scatterplot Smoothing	Cleveland and Devlin^118^	N/A
ggplot2 v.3.5.2	Wickham^119^	https://ggplot2.tidyverse.org/
STAR v.2.7.3a	Dobin et al.^120^	https://github.com/alexdobin/STAR
BPReveal v.4.0.4	McAnany et al.^51^	https://github.com/mmtrebuchet/bpreveal
tfmodisco-lite v.2.0.7 (implementation of TF-MoDISco)	Shrikumar et al.^54^	https://github.com/jmschrei/tfmodisco-lite
FIMO from MEMESuite	Grant et al.^59^	https://meme-suite.org/meme/doc/fimo.html
DESeq2	Love et al.^93^	https://github.com/thelovelab/DESeq2
Kinetic models of nucleosome-mediated TF cooperativity	This paper	https://github.com/zeitlingerlab/Weilert_mESC_accessibility_2025/blob/main/2_analysis/19_visualize_kinetics.Rmd
SciPy v.1.0	Virtanen et al.^121^	https://scipy.org/

**Other**		

All code and analyses that contributed to this work	This paper	https://github.com/zeitlingerlab/Weilert_mESC_accessibility_2025
Ensembl v.98 gene annotations for mouse genome assembly mm10	Harrison et al.^122^	https://www.ensembl.org/index.html
Bioruptor Pico sonication device	Diagenode	https://www.diagenode.com/en/p/bioruptorpico2
Neon Transfection System	Life Technologies	MPK5000
FACSymphony cell sorter	BD Biosciences	S6

### Experimental model and study participant details

#### Cell culture

R1 ESCs were cultured on 0.1% gelatin-coated plates without feeder cells in N2B27 medium (DMEM/F12 with 1:1 mix of GlutaMax/N2 and Neurobasal medium/B27, Invitrogen) supplemented with 2 mM L-glutamine (Stemcell Technologies), 1x 2-mercaptoethanol (Millipore), 1x NEAA (Stemcell Technologies), 3 μM CHIR99021 (Stemcell Technologies), 1 μM PD0325901 (Stemcell Technologies), 0.033% BSA solution (Invitrogen) and 107 U/mL LIF (Millipore).

### Method details

#### CRISPR-Cas9 motif mutations

For each of the five candidate mutations across two genomic loci described below in mouse R1 ESCs, guide RNA target sites were designed using the IDT target predictor tool by evaluating the predicted on-target efficiency score and off-target potential. Alt-R CRISPR-Cas9 crRNA was designed to contain ∼40 bases of homology from the targeted cut site (ssODN sequences are shown in Table S10). Equimolar amounts (stock of 100 μm) of Alt-R crRNA and tracrRNA-ATTO550 were mixed to form gRNA at a final concentration of 50 μM. The mixture was heated at 95°C for 5 min and cooled at RT. The single-stranded donor oligonucleotides (ssODN) were also designed to contain ∼40 bases of homology from the targeted cut site using the IDT software tool. A ribonucleoprotein (RNP) complex was formed by combining 150 pmol of gRNA (crRNA+tracrRNA) and 125 pmol of Cas9 HiFi v3 protein (IDT) with hybridization for 20 min at RT. The RNP was combined with 100 pmol of ssODN donor and 100 pmol of electroporation enhancer v2 and delivered to 1.5e5 cells by Neon electroporation (1,400 V, 10 m, 3 pulses; Neon Transfection System, MPK5000, Life Technologies). Immediately after electroporation, cells were cultured in 0.5 μM Alt-R HDR enhancer V2 of 0.69 mM. After 24 h, cells were washed with PBS before FACS sorting on S6 FACSymphony. Single cells were directly sorted into 96-well plates. Cells were screened for the expected mutations through paired-end sequencing on an Illumina MiSeq instrument (250 cycles). On-target indel frequency and expected mutations were analyzed using CRIS.py.^112^ Only clones with the intentional mutation and sequence alignments >90% were chosen for future experiments. Close selections for each candidate mutation/locus are described below. Upon clone selection, ATAC-seq and ChIP-nexus experiments were prepared alongside wild type R1 ESC control samples.

The first candidate set of mutations were located at the intronic *Btbd11* enhancer assessed in previous work.^36^ We mutated every pairwise combination of the predicted *Sox2* motif on chr10:85,539,634-85,539,643 (mm10) and the predicted low-affinity *Oct4-Sox2* motif on chr10:85,539,666-85,539,681 (mm10). This resulted in three total CRISPR mutations: (1) *Sox2* mutated, (2) *Oct4-Sox2* mutated and (3) both motifs mutated. In these cases, the *Sox2* motif was mutated from CCTTTGTTCC (wild type) to CCTAGGTTCC (mutant) and the *Oct4-Sox2* motif was mutated from AATTATAATGATAAT (wild type) to AATCATAAGGATAAT (mutant). One clonal cell line for each mutation was used to perform two biological ATAC-seq replicate experiments.

The second set of mutations were introduced at the upstream *Akr1cl* enhancer. We mutated one of the two *Sox2* motifs, located at chr1:65037654-65037663 (mm10) and chr1:65037753-65037762 (mm10). These two *Sox2* motifs contained the exact same motif sequence CCCTTTGTC (wild type) and each was mutated into the motif sequence CCCTAGGTC (mutant), which differs by two bases. One clonal cell line for each mutation was used to perform two biological ATAC-seq replicate experiments.

#### ATAC-seq experiments

For each ATAC-seq experiment, 100,000 R1 wild type or CRISPR-edited mouse embryonic stem cells were used. Cells were washed once in cold PBS, with centrifugation at 500 x g and 4°C for 5 min. The cell pellets were then resuspended in 50 μL of cold ATAC Resuspension Buffer (10 mM Tris-HCl pH 7.4, 10 mM NaCl, 3 mM MgCl2) supplemented with 0.1% IGEPAL CA-630, 0.1% Tween 20 and 0.01% Digitonin (Promega, G9441). After a 3 min incubation on ice, 1 mL of cold ATAC Resuspension Buffer supplemented with 0.1% Tween 20 was added and the samples were centrifuged at 500 x g and 4°C for 10 min. Tagmentation was performed at 1000 rpm and 37°C for 30 min in a 50 μL reaction volume containing 10 μL of 5x Tagment DNA Buffer (50 mM Tris-HCl pH 7.4, 25 mM MgCl2, 50% DMF), 0.5 μL 10% Tween 20, 0.5 μL 1% Digitonin,1 μL of 20 μM Tn5 transposase loaded with oligonucleotides and 38 μL water. For all experiments, the Tn5 used was purified in-house using pETM11-Sumo3-Tn5 and His6-tagged SenP2 protease plasmids as performed previously.^21^^,^^24^^,^^36^^,^^111^^,^^123^ DNA fragments were purified using the Monarch PCR & DNA Cleanup Kit (NEB, #T1030) and used as input for library preparation using Illumina Nextera Dual Indexing. During library preparation, qPCR was performed to prevent over-amplification^123^ and libraries were purified using a 2% agarose gel and the Monarch DNA Gel Extraction kit (NEB, #T1020). Multiple, highly correlated biological replicates were performed for all ATAC-seq experiments and paired-end sequencing was performed on an Illumina NextSeq 500 and 2000 instruments (2 × 75 bp cycles).

#### ChIP-nexus experiments

For each ChIP-nexus experiment, 10 million R1 wild type or CRISPR-edited mouse embryonic stem cells were used, as described previously.^35^^,^^36^ In brief, the cells were first washed once with PBS, crosslinked using 1% formaldehyde and quenched after 10 min using 125 mM glycine. Fixed cells were then washed twice more with ice-cold PBS and resuspended in chilled lysis buffer (15 mM HEPES pH 7.5, 140 mM NaCl, 1 mM EDTA, 0.5 mM EGTA, 1% Triton X-100, 0.5% *N*-lauroylsarcosine, 0.1% sodium deoxycholate and 0.1% SDS). After a 10 min incubation on ice, a Bioruptor Pico (Diagenode) was used to sonicate the chromatin with five or six cycles, 30 s on/off. The commercially available Sox2 antibody (Active Motif, 39843) was used for these experiments. For each ChIP, 10 μg of antibody was conjugated to 50 μL of Protein A or G dynabeads (Invitrogen). Following the ChIP, the standard ChIP-nexus library preparation, including end repair, dA-tailing, adapter ligation, barcode extension and lambda exonuclease digestion, was performed as described,^124^ except for the following two changes: the ChIP-nexus adapter mix contained four fixed barcodes, and PCR library amplification was performed directly after circularization of the purified DNA fragments (without addition of the oligo and BamHI digestion). Single-end sequencing was performed on both Illumina NextSeq 500 and Illumina NextSeq 2000 instruments (75 or 150 cycles) at the Stowers Institute. For all experiments, at least two biological replicates were performed. The current ChIP-nexus protocol can be found on the Zeitlinger lab website at https://research.stowers.org/zeitlingerlab/protocols.html.

### Quantification and statistical analysis

#### ATAC-seq data processing

Paired-end ATAC-seq reads from a doxycycline-inducible knockout cell line ZHBTc4 stored under GEO: GSE174774^66^ and paired-end ATAC-seq reads from a neuronal differentiation time course derived from WT129 mESCs under ArrayExpress: E-MTAB-8194^94^ were reprocessed alongside the paired-end sequencing reads from ATAC-seq experiments conducted in this work. Reads were pre-trimmed for adapters using Cutadapt (v.4.2)^113^ and aligned to the mouse genome assembly mm10 using bowtie2 (v.2.3.5.1).^114^ Any CRISPR ATAC-seq experiments were aligned to a custom mm10 genome assembly modified to contain the expected CRISPR mutations. Duplicated alignments were marked using Picard (v.2.23.8).^115^ Alignments were then deduplicated, filtered for fragment lengths exceeding 600 bp, corrected for dovetailed alignment pairs and end-adjusted to accommodate the Tn5 enzymatic cut correction of −4/+4. Normalized read-per-million (RPM) ATAC-seq tracks were generated by scaling the aligned coverage to the weighted total reads as described previously.^21^ Cut site ATAC-seq coverage used for training ChromBPNet models were generated by isolating the bases on each end of an aligned fragment, consolidating those “cut sites” into coverage tracks. ATAC-seq peaks were mapped using MACS2 (v.2.2.7.1)^116^ with default settings. High-confidence peaks were then kept from the most reproducible replicate pair using the Irreproducible Discovery Rate framework (IDR) (v.2.0.3)^117^ for downstream analysis and model training. Whenever visualizing observed ATAC-seq cut site coverage at individual loci, we used ggplot2 (v.3.5.2) to implement Locally Estimated Scatterplot Smoothing (span = 0.2) rather than ATAC-seq fragment pileup.^118^^,^^119^

#### ChIP-nexus data processing

Single-end sequencing reads from GEO: GSE137193 were reprocessed alongside the single-end sequencing reads from ChIP-nexus experiments conducted in this work. Reads were preprocessed by trimming off fixed and random barcode components from the sequenced reads, reassigning barcode information as read names for downstream processing. Next, reads were pre-trimmed for adapters using Cutadapt (v.4.2)^113^ and aligned to the mouse genome assembly mm10 using bowtie2 (v.2.3.5.1).^114^ Any CRISPR ChIP-nexus experiments were aligned to a custom mm10 genome assembly modified to contain the expected CRISPR mutations. Alignments were then deduplicated from aligned coordinates and their corresponding barcodes. Coverage was generated pooling the first “stop” base of each deduplicated read, separated into two tracks by read orientation. Normalized read-per-million (RPM) ChIP-nexus tracks were generated by scaling the aligned coverage to the weighted total reads as described previously.^21^ ChIP-nexus peaks were mapped using MACS2 (v.2.2.7.1)^116^ with parameters designed to resimulate the full fragment length coverage rather than the single stop base coverage (--keep-dup = all -f = BAM --shift = −75 --extsize = 150). High-confidence peaks were then kept from the most reproducible replicate pair using the Irreproducible Discovery Rate framework (IDR) (v.2.0.3).^117^ Peaks were then filtered for non-standard chromosomes, sequence ambiguities and presence on chromosome boundaries. After filtering, 154,827 peaks were used for downstream analysis and model training.

#### TT-seq data processing

Paired-end TT-seq reads from a doxycycline-inducible knockout cell line ZHBTc4 stored under GEO: GSE174774 were reprocessed. Using STAR (v.2.7.3a)^120^ on default settings, transcript reads were both aligned to the mouse genome assembly mm10 and collected into gene counts tables derived from Ensembl v.98 gene annotations.^122^ Alignments were then deduplicated, filtered for fragment lengths exceeding 600 bp and corrected for dovetailed alignment pairs. We generated strand-separated genome-wide coverage using the by isolating the 5′ ends of each aligned read, thus representing each single-base read as an “event of transcription”, rather than fragment pileup. Normalized read-per-million (RPM) TT-seq single-base tracks were generated by scaling the stranded, aligned coverage to the weighted total reads as described above for ATAC-seq and as done previously.^21^

#### Model training and validation

BPNet is a convolutional network designed to learn genomic data from input DNA sequences alone.^36^ Using the BPReveal implementation^51^ (v.4.0.4) of BPNet’s previously described architecture, we trained a multi-task model to learn the ChIP-nexus binding of Oct4, Sox2, Nanog, Klf4 and Zic3 signals. Input DNA sequences were derived from the sequences of 154,827 IDR-reproducible peak regions, described above. Of these peak regions, 71% of them overlapped with IDR-reproducible ATAC-seq peaks (*n* = 113,160). We validated that these genomic regions possessed sufficiently diverse accessibility for ChromBPNet model to distinguish between accessible and inaccessible loci by comparing their interquartile ranges (IQR). The IQR of IDR-reproducible ATAC-seq peaks was 1,142 reads with quartile ranges of [513,1655] reads. The IQR of IDR-reproducible ATAC-seq peaks was 1,749 reads with quartile ranges of [384,1,749] reads. Because the binding peaks covered a broader range of accessibility levels, we deemed it as an appropriate dataset by which to train both binding (BPNet) and accessibility models (ChromBPNet). Models used for downstream analysis were validated on 27,866 sequences from chromosomes 2/3/9, tested on 25,351 sequences from chromosomes 1/8/17 and trained on 101,451 sequences from the remaining standard chromosomes. Post-optimization, the final BPNet binding model was trained with 8 convolutional layers, a filter depth of 128, an input filter length of 7 and an output filter length of 7. Model performance was determined based on Spearman correlations of predicted versus observed total experimental counts, Jensen-Shannon distances of predicted and observed profiles and multinomial negative log-likelihoods of predicted and observed profiles. With this associated architecture, input DNA sequences were 2032 bp long, resulting in the desired output prediction window of 1000 bp, as BPReveal’s reimplementation of BPNet does not pad through convolutions, but rather requires a larger input sequence to provide the receptive field with sufficient sequence information as the model deepens. Model stability was cross-validated by training two other models with different training, validation and test regions and assessed via described performance metrics alongside consistently learned sequence patterns upon application of BPReveal’s implementation of interpretation tool DeepSHAP^52^^,^^53^ and the tfmodisco-lite (v.2.0.7) implementation of TF-MoDISco.^36^^,^^54^

ChromBPNet is a modified training approach to BPNet, applying BPNet convolutional architecture to learning ATAC-seq data in a two-step model training process to eliminate the positional Tn5 sequence biases of ATAC-seq experiments.^38^ Using the BPReveal implementation^51^ (v.4.0.4) of ChromBPNet, we trained the first “Tn5 bias” model on 22,053 inaccessible, low-count reads from our pooled ATAC-seq experimental coverage in wild type mESCs, using the same training, validation and test chromosomes described above for the final BPNet binding model. We confirmed that this bias model did not learn TF binding motifs and that only the expected Tn5 bias sequence patterns were returned through assessing model predictions upon *in silico* motif injection alongside application of BPReveal’s implementation of interpretation tool DeepSHAP^52^^,^^53^ and tfmodisco-lite (v.2.0.7) implementation of TF-MoDISco.^36^^,^^54^ We next trained a second, residual model alongside the now-frozen bias model to explain bias-removed ATAC-seq accessibility using the same 154,827 sequences split between the same training, validation and test sets used in the BPNet model training, described above. These regions were confirmed to contain an appropriately diverse range of accessibility to train a robust ChromBPNet model. During this training step, we considered only the positional information provided by the bias model when assessing output predictions. We optimized ChromBPNet model architecture using the same performance metrics described above for the BPNet binding model and post-optimization, the final residual ChromBPNet model was trained with 8 convolutional layers, a filter depth of 128, an input filter length of 7 and an output filter length of 7. With this associated architecture, input DNA sequences were 2032 bp long, resulting in the desired output prediction window of 1000 bp. Model stability was first assessed with cross-validation training, performed identically to BPNet models described above. Model stability was next assessed by training additional cross-validated accessibility models under three different region sets including 2,553,950 genome-wide regions, 113,117 accessible regions from IDR-reproducible ATAC-seq peaks, and finally an even combination of the accessible regions just described with an additional 113,117 regions sampled from sufficiently inaccessible loci totaling to 226,069 regions. Differences in model performance were small and are reported in Table S2. It was also confirmed that all models learned similar contextual and cooperative effects of key pioneer motifs, reported in Table S3. TF-MoDISco motifs returned by deep-learning models, related to Figure 1, Table S4. Correlations of motif strength and function, related to Figure 2, Table S5. Correlations of motif arrangements and function, related to Figure 3, Table S6. Motif cooperativities across deep-learning models, related to Figure 4.

To capture accessibility rules after depletion of Oct4,^66^ a separate ChromBPNet model was trained for each of the six time points (0h, 3h, 6h, 9h, 12h and 15h) from the reprocessed GEO: GSE174774 ZHBTc4 ATAC-seq data in mESCs, using the same bias model, optimized architecture and cross-validated region sequence sets as the original mESC R1 ChromBPNet model. Model performance and stability were determined using the same performance metrics described above for the ChromBPNet accessibility models and the same cross-validation training approach. After confirming that the models were stable and performed well, we also validated that the sequence rules and motifs returned by the initial time point (0h) ChromBPNet model of the ZHBTc4 cell line were comparable to our wild type R1 ChromBPNet model.

To capture accessibility rules over a neuronal differentiation time course,^94^ a separate ChromBPNet model was trained for each of the four time points (day 0, day4, day 8, day 12) from the reprocessed ArrayExpress: E-MTAB-8194 WT129 ATAC-seq data in mESCs, using the same bias model and optimized architecture as the original mESC R1 ChromBPNet model. While cross-validated chromosome selection remained the same as the original mESC R1 ChromBPNet model, each model belonging to the neuronal differentiation time course was trained on a region set consisting of merged IDR-reproducible regions from each time point, resulting in 78,369 regions for training, tuning and testing. Model performance and stability were determined using the same performance metrics described above for the ChromBPNet accessibility models and the same cross-validation training approach. After confirming that the models were stable and performed well, we also validated that the sequence rules and motifs returned by the initial time point (day 0) ChromBPNet model of the WT129 cell line were comparable to our wild type R1 mESC ChromBPNet model.

#### Motif mapping

For all trained ChromBPNet residual accessibility and BPNet binding models, we generated sequence contribution scores using BPReveal’s implementation^51^ (v4.0.4) of DeepSHAP, modified to generate hypothetical contribution scores akin to those from DeepLIFT.^52^^,^^53^ Using counts contribution scores (explaining the total amount of binding or accessibility across a region) inputted to the tfmodisco-lite implementation of TF-MoDISco^36^^,^^54^ (v2.0.7, https://github.com/jmschrei/tfmodisco-lite), we generated contributing motif representations called CWMs (contribution weight matrices) for each trained model. For all model/TF-MoDISco runs, we manually curated motif/TF identities based on existing literature, returning expected pioneer motifs and motifs important for TF binding in each corresponding cell type and validated that the cross-validated models returned the same motif representations.

It should be noted that while the *Oct4* motif was discovered in mESC models, this motif was not robustly discovered across model folds for either binding or accessibility, mapped with low frequencies (*n* = 132), produced poor Oct4 ChIP-nexus footprinting across mapped motifs and was often enriched in ERV regions. Thus, we excluded the *Oct4* motif from further downstream analysis.

To map the TF-MoDISco CWM motif representations back to the genome, we performed contribution weight matrix scanning (CWM scanning) implemented by BPReveal^51^ (v4.0.4), as described previously.^36^ Briefly, across all regions considered for each respective model training, we mapped a motif if the genomic region matched criteria designated by the corresponding TF-MoDISco motif distributions. The criteria used in motif mapping here required that (1) the Jaccardian similarity between the motif CWM and the genomic site’s sequence contribution exceeded the 20th percentile of the TF-MoDISco motif representations and (2) the contribution L1 magnitude is higher than the TF-MoDISco motif seqlets lowest contribution score. Unlike the previous approach,^36^ we did not require that the log-odds score relative to the TF-MoDISco motif PWM be greater than zero. This allowed us to rely solely on contribution similarity and magnitude, to encourage our mapping strategy to allow very degenerate sequences, provided they possess sufficiently well-positioned counts contributions across the motif coordinate bases. Upon CWM scanning, we refined the mappings by resolving redundant (e.g., *Oct4-Sox2* and *Sox2*) and palindromic mappings. For each motif, its percentile relative to the original seqlet distributions of CWMs (Jaccardian similarity) and PWMs (information content) was calculated. We note here that unless otherwise explicitly stated, PWM scores refer to the information content score and not the log-odds score.

For our R1 mESC models, we performed PWM-scanning in order to benchmark our contribution-derived mapping approach using the FIMO tool from MEMESuite^59^^,^^60^ (--skip-matched-sequence --max-strand --thresh 0.001) with TF-MoDISco defined seqlets as the consensus frequencies across only the corresponding 154,827 regions of interest used in R1 mESC models. Thus, all comparisons between CWM-mapped and PWM-mapped motifs were conducted on the same region set. Thresholds of significant matches (*p* = 0.001) were set low enough to return common and poor mappings.

#### Isolation and context scores

Previous work has shown that predicting the log-fold-change effects of an injected TF binding motif over a random *in silico* background using BPNet can result in a strong proxy for motif affinity.^67^ Referred to here as the “isolation” scores of an injected sequence, this injection strategy removes the surrounding genomic context of a mapped motif to measure its intrinsic effects out of context. Briefly, a sequence was injected into randomly generated 256 background sequences, predictions were measured with and without the injected sequence, then the log-fold-change was calculated between averaged injected and background levels. Background sequences were derived by sampling trained peak regions to match the baseline CpG enrichment and GC-content of the mouse genome, then di-nucleotide shuffling the underlying sequences. CpG ratios were calculated as (GC-content/2)^2^ and normalized to each region width, as previously described.^125^ Since the background sequences may contain functional motifs during in silico injections, we average the effect of 256 trials, which averages out these spurious effects.

In contrast to isolation scores, the “context” scores of a mapped motif involves measuring a motif’s effect within its genomic context rather than in isolation, as described previously.^21^ Mapped motif sequences were mutated via replacement with random insertions. Across 16 trials of mutations, average mutation response was calculated, then log-fold-change was computed between mutated and wild type profiles. If two or more motifs are in the same region, but do not share overlapping coordinates and are at least 20 bp apart center-to-center, individual context scores are calculated for each motif.

#### Cooperativity characterization

To characterize the amount of accessibility allocated to non-motif effects, motif effects and cooperativity in a genomic region we performed two *in silico* mutation experiment sets for each region containing at least two pioneering motifs mapped by the accessibility model (derived from Figure S1C). For all motif mutations described henceforth, we performed 16 random sequence replacements, averaging the mutated response to a single value. To capture non-motif effects on predicted accessibility, we simultaneously mutated every mapped pioneer motif coordinate in the region and measured the predicted response (null). We next measured the accessibility upon simultaneously mutating every other mapped pioneer motif (A), subtracting it from the (null) to capture a motif’s individual effects on predicted accessibility (A_only_ = A - null). We added the individual effects of every motif in a region (A_only_) together to consider total motif effects in a region (all_only_). Beyond non-motif effects and individual motif effects, we deemed the remainder predicted accessibility in the wild type sequence (WT) as due to pioneer motif cooperativity, calculating it as (cooperativity = WT - all_only_ - null).

When considering only a pair of mapped motifs, we considered them as cooperative when they produced accessibility greater than the sum of their individual parts. More precisely, for each pair of mapped motifs we predicted the accessibility effects across the *in vivo* wild type sequence (AB), sequences with one motif mutated (A or B) and sequences with both motifs mutated (null). We characterized accessibility effects as the total predicted ATAC-seq reads within the surrounding 1 kb region. These total predicted reads were exponentiated from the log-space counts outputs of BPReveal models. As previously described,^19^ “joint” effects of paired motifs were characterized as (AB - null). “Marginal effects” of individual motifs were characterized as ((A - null) + (B - null)). Cooperativity scores were assigned by taking the log-fold-change of the joint effects over the marginal effects for all mapped motif pairs. When testing whether a specific type of motif pair was significantly cooperative, we used a one-tailed Wilcoxon test comparing whether mapped motif pairs belonging to that type arranged at specified distances (typically bins of 10 bp) had greater cooperativity scores than mapped motif pairs at distances that were deemed too long to correspond to cooperative pioneering (greater than 400 bp), with multiple-comparison Bonferroni corrections and an adjusted significance cutoff requirement of *p* < 10^−7^.

#### PBM comparisons

Processed PBM experiments that were close matches to our TFs of interest were downloaded from UniPROBE^64^ in order to compare the *in vitro* binding Z-scores of each measured 8-mer sequence to the PWM and isolation scores of our TF/motif pairs. To compare PBM Z-scores to either mapped motif PWM scores or isolation scores, we trimmed the mapped motif sequences down to the expected “core” 8-mer sequences. If this trimming resulted in multiple redundant 8-mers, we took the one with the maximum isolation score (PWM scores would be identical). When 8-mer sequences were redundant within the PBM datasets due to reverse complement matches, we took the maximum *Z* score. We compared human Sox2 PBM data to our Sox2/*Sox2* TF/motif pair.^61^ We compared mouse Klf1 PBM data to our Klf4/*Klf4* TF/motif pair.^62^ We compared human Zic3 PBM data to our Zic3/*Zic3* TF/motif pair.^63^ We compared human POU5F1 PBM data to our Oct4/*Oct4-Sox2* TF/motif pair, favoring the *Oct4* motif component of the heterodimer *Oct4-Sox2* motif when trimming down 8-mers.^61^

#### Differential enrichment analysis

In order to measure differential enrichment of accessibility and enhancer RNA across changing Oct4 concentrations, we performed DESeq2^93^ on the reprocessed ATAC-seq and TT-seq data derived from a time course experiment with decreasing Oct4 levels after doxycycline withdrawal.^66^ DESeq2 was performed in all cases with default parameters and FDR = 0.05. For each DESeq2 model described below, we conducted pairwise comparisons between the wild type time point (0h) and all corresponding mutant time points (3h–15h), computing the log2(mutant/wt) values for each. As we previously described,^21^ log2(mutant/wt) < 0 will represent a loss in signal enrichment in the mutant, while log2(mutant/wt) > 0 represents a gain in enrichment in the mutant.

More specifically, to measure differential accessibility across accessible regions of interest over changing Oct4 concentrations, we built a DESeq2 model containing ATAC-seq cut site coverage for every replicate and time point within the 154,827 genomic regions (see “Model training and validation”) used for model training. To measure differential enhancer expression over changing Oct4 concentrations, we used the same procedure described above for differential accessibility. We collected base-resolution TT-seq coverage across the 154,827 accessible regions of interest for every replicate and time point, building a DESeq2 model from the collected coverage.

To measure differential target gene expression over changing Oct4 concentrations, we built a DESeq2 model containing TT-seq coverage across gene counts tables derived from Ensembl v.98 gene annotations.^122^ We next kept all genes that contained only one *Oct4-Sox2* or *Sox2*-containing region within 100 kb, to avoid the confounding influence of multiple enhancer-gene target effects. Following this, median target gene expression levels were calculated over changing Oct4 concentrations for different motif arrangements of *Oct4-Sox2* or *Sox2* that were within 5 kb of the target gene start.

To measure differential enrichment of accessibility across a neuronal differentiation time course, we performed DESeq2 as described above, considering instead the 78,369 regions found to be accessible via the neuronal differentiation ATAC-seq time course.

#### Nucleosome-mediated TF cooperativity modeling

Nucleosome-mediated TF cooperativity has often been modeled under thermodynamic equilibrium,^27^^,^^79^^,^^81^ where the chromatin exists in two conformations, closed (with nucleosome) and open (without), with basal transitions between the closed and the open conformations. In these models, a TF promotes the open conformation when the affinity of the TF is higher in the open conformation than in the closed conformation. Given the assumption of thermodynamic equilibrium, this implies an equivalent reduction in the affinity of the nucleosome in the open chromatin conformation relative to the closed conformation, thereby accounting for a competition between the TF and the nucleosome. This model contradicts however the concept of a pioneer TF, whose ability to open chromatin depends on its ability to bind the nucleosome in the closed state.^18^ Intuitively, pioneering TFs should produce strong opening effects that do not entirely rely on higher affinity binding in the open state. To achieve this, we chose to consider a kinetic model, a two-conformation TF-nucleosome model away from equilibrium. This allowed us to separate the effect of the nucleosome on TF binding, which can be small, from the opening effect of the TF, which can be strong.

For the single motif case, we consider a model with 4 states: (1: closed, unbound), (2: closed, bound), (3: open, unbound), (4: open, bound), with reversible transitions between states 1–2, 1–3, 2–4, 3–4 (Figure S5B). In order to account for 2 TF motifs, the model is extended to 8 states, accounting for all possibilities of TF and nucleosome bound or not, and the corresponding transitions (Figure 5C). In both cases, the system is assumed to evolve over time according to Markovian dynamics, which settles into a steady-state. To quantify accessibility, we consider the sum of the steady-state probabilities of the open states, here represented as states 3 and 4 for the single TF model (Figure S5B). Such steady-state probabilities can be calculated by solving for the null space of the transition rate matrix (formally, the Laplacian of the graph associated with the system). For a model with N states, this is a square matrix of size *N x N*, where the value of the entry in position *i, j* contains the transition rate from state *j* to *i* if *i ≠ j*, and the diagonals contain the negative of the sum of the rest of the column.^126^^,^^127^ For a given set of parameters, we obtain a basis for the nullspace of this matrix using SciPy (v.1.12.0, scipy.linalg.null_space)^121^ and obtain the steady-state probabilities by normalizing each entry by the total sum, such that the probabilities sum to one.

As we saw, a TF has motifs of different affinities, whose effective affinity *in vivo* can be modulated by the surrounding sequence context. Similarly, the effect of the TF once bound at the motif may also depend on surrounding molecules. Therefore, in order to explore how the model behaved for a single TF motif and for two TF motifs, we sampled parameter sets accounting for potential variation in the motif binding affinity and effect of the TF(s) once bound.

For a given parameter set, we consider that the TF binding rate (*k’*_*on*_) is independent from whether or not the motif or the nucleosome is present. This is in line with an assumption for a diffusion-limited binding rate. TF concentration is absorbed in the apparent TF association rate (*k*_*on*_, such that *k*_*on*_ = *k’*_*on*_ *[A]*, with *[A]* the concentration of TF A, such that changing concentrations is simulated by changing *k*_*on*_). Similarly, we consider motif-independent basal opening rate (*k*_*open*_) and closing rate (*k*_*close*_), and the effect of the TF on these rates, which are modulated by a factor *o* for the opening rate and *c* for the closing rate when the TF is bound. We select a value for the unbinding rate of the TF from the open conformation in the absence of TF motif (non-specific unbinding rate on naked DNA, *k*_*off*_). This unbinding rate is assumed to be reduced by a factor *g* in the presence of a motif. The destabilizing effect of the nucleosome on the TF is also introduced in the unbinding rate, which is increased by a factor (*β*_*n*_) in the absence of motif, and a factor (*β*_*m*_) in the presence of a motif. Given these parameters, we can calculate the steady-state probability of the accessible conformation for the parameters corresponding to the motif being present, and for those corresponding to the motif being absent, such that we can calculate the log-fold-change in accessibility. This provides an equivalent metric to the isolation scores derived from deep learning predictions of sequence injections *in silico*.

For two TFs A and B, a similar approach applies where we expand the model to consider four sequence scenarios: no motifs, motifs A and B, motif A only and motif B only. When two TFs are bound, we must make an assumption for what *o*_*AB*_ and *c*_*AB*_ are. If *o*_*A*_ is the factor increase in the opening rate when TF is bound at motif A and *o*_*B*_ is the factor increase in the opening rate when TF is bound at motif B, we choose *o*_*AB*_ *= o*_*A*_ *x o*_*B*_ for the opening rate when both are bound. The same multiplicativity assumption is made for the joint effects on the closing rate *c*_*AB*_. This multiplicativity arises naturally if we assume that there is an activation energy *E* associated to a transition rate *k*, with each TF having an additive effect on this activation energy *x*_*A*_ or *x*_*B*_. Following the Arrhenius equation:

k=C×exp(−E/(RT))

$$k_{A}=C\times \exp\left(-\left(E-x_{A}\right)/\left(RT\right)\right)=k\times o_{A}$$

$$k_{B}=C\times \exp\left(-\left(E-x_{B}\right)/\left(T\right)\right)=k\times o_{B}$$

$$k_{A,B}=C\times \exp\left(-\left(E-x_{A}-x_{B}\right)/\left(RT\right)\right)=k\times o_{A}\times o_{B}$$

When simulating pioneering TF binding to its respective motif, we typically chose to model Sox2 binding to the *Sox2* and *Oct4-Sox2* motifs. We chose parameters from plausible biological ranges, described below, depicted graphically (Figure S5B) and reported each parameter regime for every simulation conducted in Table S7. Parameter selection for 1TF kinetic model in equilibrium, related to Figure 5, Table S8. Parameter selection for 1TF kinetic model out of equilibrium, related to Figure 5, Table S9. Parameter selection for 2TF kinetic model out of equilibrium, related to Figure 5.

For TF apparent binding on rates (*k*_*on*_), we assume a diffusion-limited binding on rate. This rate is therefore unaffected by whether or not there is a nucleosome and unaffected by whether or not there is a motif. Diffusion-limited binding rates for TFs have been estimated to be on the order of 10^−2^-10 s^−1^nM^−1^.^128^ For Sox2, an *in vitro* binding constant for the DNA binding domain has been measured to be 0.75∗10^−3^ s^−1^nM^−1^.^90^ The Sox2 concentration in mESCs is on the order of 60–120 nM,^87^ which would correspond to an apparent binding rate of 0.045–0.09 s^−1^. In line with these numbers, the apparent *k*_*on*_ for Sox2 has been previously estimated using single-molecule tracking^89^ to have a value of 0.27 s^−1^. Therefore, we typically chose an intermediate of *k*_*on*_ = 0.1s^−1^, which we kept fixed throughout all simulations.

Given that *k*_*on*_ is fixed for a given concentration, it is the TF binding off rates (*k*_*off*_) that account for differences in affinity due to nucleosome or motif presence. For TF binding off rate from the open conformation without motif (*k*_*off,4→3, no motif*_), indicating a non-specific off rate, we chose a value of *k*_*off,4→3, no motif*_ = 1 s^−1^, in line with previous observations.^89^ For TF binding off rate from the open conformation with the motif (*k*_*off,4→3, motif*_), this off rate is reduced by a factor *g* taken to be between *g* = [0.001, 0.9]. These values are aimed to cover very low-affinity motifs (high *g*∼1), stronger motifs which have been measured *in vitro* to have Sox2 binding affinities of up to 10-fold that of naked DNA (*g* = 0.1),^88^ and potentially higher motif affinities that could arise through binding cooperativity with TFs that we do not model explicitly (lowest *g*≪1). For TF binding off rate from the nucleosomal conformation without motif (*k*_*off,2→1, no motif*_), this off rate is increased by 5-fold relative to that for the open conformation,^88^ specified by *β*_*n*_ in the graphical depiction (Figure S5B). For the TF binding off rate from the nucleosomal conformation with motif (*k*_*off,2→1, motif*_), specified by *β*_*m*_, this off rate is decreased by a factor taken from the range *β*_*m*_ = [2,10].

We assumed a basal chromatin opening rate (*k*_*open*_), in the absence of a bound TF and independently of whether there is or not the motif, to be *k*_*open*_ = 0.001 s^−1^, corresponding to a half-life in the order of approximately 10–20 min. To choose the basal closing rate (*k*_*close*_), we defined the nucleosome dissociation constant *d*, which we chose from a given range, and set *k*_*close*_ = *k*_*open*_/*d*. Note that in the absence of a bound TF, the probability of the system being open is *d*/(1 + *d*) ∼ *d* for *d*≪1. We typically chose a range for *d* = [0.001, 0.05] to model a predominantly closed region in the absence of a bound TF. This corresponds to *k*_*close*_ = [0.02, 1] s^−1^. Iurlarlo et al. measured the closing dynamics of chromatin upon SWI/SNF inhibition in mESCs and found that by 10 min, many sites exhibited a noticeable decrease in openness.^129^ This suggests a half-life for the closing reaction in the presence of TFs on the order of a few minutes. Therefore, it is plausible that the basal closing rate is even faster and consistent with rates on the order of seconds as we chose. With the TF bound, the chromatin opening rate (*k*_*open*_) is increased by a factor *o* and the chromatin closing rate is decreased by a factor *c*, which are reported in Table S7. Parameter selection for 1TF kinetic model in equilibrium, related to Figure 5, Table S8. Parameter selection for 1TF kinetic model out of equilibrium, related to Figure 5, Table S9. Parameter selection for 2TF kinetic model out of equilibrium, related to Figure 5.

## Supplemental information

Document S1. Figures S1–S6 and Table S11

Table S1. Performances of deep-learning model optimization, related to Figure 1

Table S2. Performances of final deep-learning models, related to Figure 1

Table S3. TF-MoDISco motifs returned by deep-learning models, related to Figure 1

Table S4. Correlations of motif strength and function, related to Figure 2

Table S5. Correlations of motif arrangements and function, related to Figure 3

Table S6. Motif cooperativities across deep-learning models, related to Figure 4

Table S7. Parameter selection for 1TF kinetic model in equilibrium, related to Figure 5

Table S8. Parameter selection for 1TF kinetic model out of equilibrium, related to Figure 5

Table S9. Parameter selection for 2TF kinetic model out of equilibrium, related to Figure 5

Table S10. CRISPR single-stranded donor oligonucleotide sequences

Document S2. Transparent peer review records for Weilert et al.

Document S3. Article plus supplemental information
