# Supplementary material for: Cooperativity enables widespread role of low-affinity motifs in chromatin accessibility and increases regulatory potential
Source: Cell Genom. 2026 Aug 25;6(10):101339. doi: 10.1016/j.xgen.2026.101339 (PMC13689504; doi:10.1016/j.xgen.2026.101339)
Supplement: Supplementary file 1 [file mmc1.pdf]

Cell Genomics, Volume *6*

Supplemental information

Cooperativity enables widespread role of low-affinity motifs in chromatin accessibility and increases regulatory potential

Melanie Weilert, Kaelan J. Brennan, Khyati Dalal, Sabrina Krueger, Haining Jiang, Rosa Martinez-Corral, and Julia Zeitlinger

Supplemental information

Cooperativity enables widespread role of low-affinity motifs in chromatin accessibility and increases regulatory potential

Figure S1

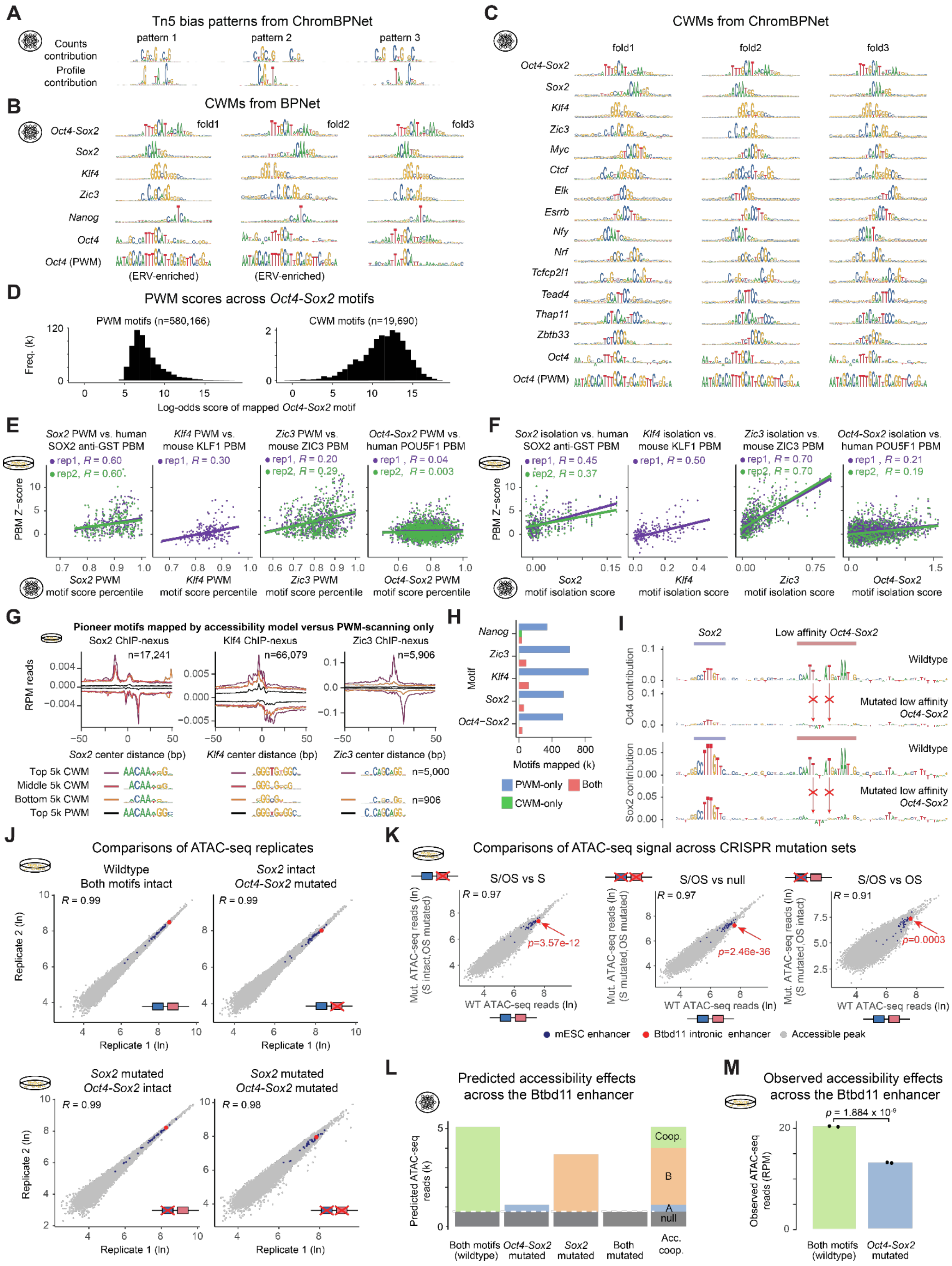

A
Tn5 bias patterns from ChromBPNet
pattern 1
pattern 2
pattern 3
Counts contribution
Profile contribution
B
CWMs from BPNet
fold1
fold2
fold3
Oct4-Sox2
Sox2
Klf4
Zic3
Nanog
Oct4
Oct4 (PWM)
(ERV-enriched)
C
CWMs from ChromBPNet
Oct4-Sox2
Sox2
Klf4
Zic3
Myc
Ctcf
Elk
Esrrb
Nfy
Nrf
Tcfcp2l1
Tead4
Thap11
Zbtb33
Oct4
Oct4 (PWM)
D
PWM scores across Oct4-Sox2 motifs
PWM motifs (n=580,166)
CWM motifs (n=19,690)
Freq. (k)
Log-odds score of mapped Oct4-Sox2 motif
E
Sox2 PWM vs. human SOX2 anti-GST PBM
Klf4 PWM vs. mouse KLF1 PBM
Zic3 PWM vs. mouse ZIC3 PBM
Oct4-Sox2 PWM vs. human POU5F1 PBM
rep1, R = 0.60
rep2, R = 0.60
rep1, R = 0.30
rep1, R = 0.20
rep2, R = 0.29
rep1, R = 0.04
rep2, R = 0.003
PBM Z-score
Sox2 PWM motif score percentile
Klf4 PWM motif score percentile
Zic3 PWM motif score percentile
Oct4-Sox2 PWM motif score percentile
F
Sox2 isolation vs. human SOX2 anti-GST PBM
Klf4 isolation vs. mouse KLF1 PBM
Zic3 isolation vs. mouse ZIC3 PBM
Oct4-Sox2 isolation vs. human POU5F1 PBM
rep1, R = 0.45
rep2, R = 0.37
rep1, R = 0.50
rep1, R = 0.70
rep2, R = 0.70
rep1, R = 0.21
rep2, R = 0.19
Sox2 motif isolation score
Klf4 motif isolation score
Zic3 motif isolation score
Oct4-Sox2 motif isolation score
G
Pioneer motifs mapped by accessibility model versus PWM-scanning only
Sox2 ChIP-nexus
Klf4 ChIP-nexus
Zic3 ChIP-nexus
n=17,241
n=66,079
n=5,906
RPM reads
Sox2 center distance (bp)
Klf4 center distance (bp)
Zic3 center distance (bp)
Top 5k CWM
Middle 5k CWM
Bottom 5k CWM
Top 5k PWM
n=5,000
n=906
H
Nanog
Zic3
Klf4
Sox2
Oct4-Sox2
Motif
Motifs mapped (k)
PWM-only
Both
CWM-only
I
Sox2
Low affinity Oct4-Sox2
Wildtype
Mutated low affinity Oct4-Sox2
Oct4 contribution
Sox2 contribution
J
Comparisons of ATAC-seq replicates
Wildtype Both motifs intact
Sox2 intact Oct4-Sox2 mutated
Sox2 mutated Oct4-Sox2 intact
Sox2 mutated Oct4-Sox2 mutated
R = 0.99
R = 0.98
Replicate 2 (ln)
Replicate 1 (ln)
mESC enhancer
Btbd11 intronic enhancer
Accessible peak
K
Comparisons of ATAC-seq signal across CRISPR mutation sets
S/OS vs S
S/OS vs null
S/OS vs OS
R = 0.97
R = 0.91
p=3.57e-12
p=2.46e-36
p=0.0003
Mut. ATAC-seq reads (ln) (S intact,OS mutated)
Mut. ATAC-seq reads (ln) (S mutated,OS mutated)
Mut. ATAC-seq reads (ln) (S mutated,OS intact)
WT ATAC-seq reads (ln)
L
Predicted accessibility effects across the Btbd11 enhancer
Predicted ATAC-seq reads (k)
Coop.
B
A
null
Both motifs (wildtype)
Oct4-Sox2 mutated
Sox2 mutated
Both mutated
Acc. coop.
M
Observed accessibility effects across the Btbd11 enhancer
p = 1.884 x 10⁻⁹
Observed ATAC-seq reads (RPM)
Both motifs (wildtype)
Oct4-Sox2 mutated

Figure S1. Sequence-to-function binding and accessibility models can learn functional low-affinity motifs, related to Figure 1

A) The ChromBPNet Tn5 bias model learned expected bias motifs, shown as PWMs for the top three patterns, summarized by TF-MoDISco from sequences with high counts contribution or profile contribution scores.

B and C) Motifs shown as CWMs learned by **(B)** the BPNet binding model and **(C)** the ChromBPNet accessibility model across different folds as summarized by TF-MoDISco. While most motifs were robustly discovered in all folds, the single *Oct4* motif was not robustly discovered, often returning ERV-enriched mappings as these are recognizable in the *Oct4* PWMs (bottom row).

D) *Oct4-Sox2* motifs learned by the accessibility model showed a distribution of motif affinities based on PWM log-odds scores (left) or CWM log-odds scores (right).

E and F) Scatterplots showing a relationship between model-derived motif features and available *in vitro* measured PBM binding data for human SOX2, mouse KLF1, mouse ZIC3 and human POU5F1 (OCT4), the TFs in the UniPROBE's database that best match our TFs of interest. The PBM binding data are summarized as Z-scores for the 8-mer sequences that match the *Sox2*, *Klf4*, *Zic3* and *Oct4-Sox2* motifs. **(E)** The PWM score percentiles of all uniquely mapped motif sequences and **(F)** the corresponding binding isolation scores both correlate with the PBM Z-scores, as shown by Spearman correlation scores. PBM replicate experiments are shown in separate colors.

G) The top, middle and bottom 5,000 *Klf4, Sox2* and *Zic3* pioneer motifs based on the PWM scores are shown with their PWM logos (bottom) and alongside the average ChIP-nexus profiles of the corresponding TF (top).

H) Comparison of the number of motifs discovered only by PWM scanning, only by CWM scanning or both, using a low-stringency cutoff for PWM scanning necessary for discovering low-affinity motifs. As expected, PWM scanning discovered vastly more motifs. Motifs discovered by CWM scanning were typically a subset of the motifs discovered by PWM scanning, except for *Nanog* motifs, which are more difficult to map with PWM scanning.

I) The intronic *Btbd11* enhancer with contribution scores from the BPNet binding model for Oct4 and Sox2 across the wildtype sequence (top) and mutated sequence (bottom). This shows that mutating the low-affinity *Oct4-Sox2* motif abolishes the predicted binding of Oct4 and Sox2. Note that the *Sox2* motif on the left is still predicted to be bound, although with slightly lower contribution in the mutant.

J) ATAC-seq replicate comparisons show that the ATAC-seq experiments on wildtype and CRISPR clones mutating the *Btbd11* enhancer are reproducible (Spearman correlation coefficients reported on the scatterplots). Total reads were measured across ATAC-seq peaks found in wildtype experiments.

K) Direct comparisons of ATAC-seq reads between wildtype and CRISPR *Btbd11* mutant experiments. As expected, the Spearman correlation coefficients are overall high but show differential accessibility across the *Btbd11* region, as calculated by DESeq2 with adjusted p values (red arrows).

L) Accessibility across the *Btbd11* region as predicted by the ChromBPNet model for wildtype and after the high-affinity *Sox2* and/or low-affinity *Oct4-Sox2* motif sequences were mutated. Changes were measured across coordinates chr10:85539400-85539800. To the right, predicted cooperative effects (green) are stacked upon individual marginal effects of motifs.

M) RPM-normalized quantifications of total ATAC-seq cut sites across replicate experiments (black dots) at coordinates chr10:85539400-85539800 across wildtype (green) and the low-affinity *Oct4-Sox2* mutant (blue) cell lines. Significance was tested via a one-tailed t-test between replicate experiments (black dots).

Figure S2

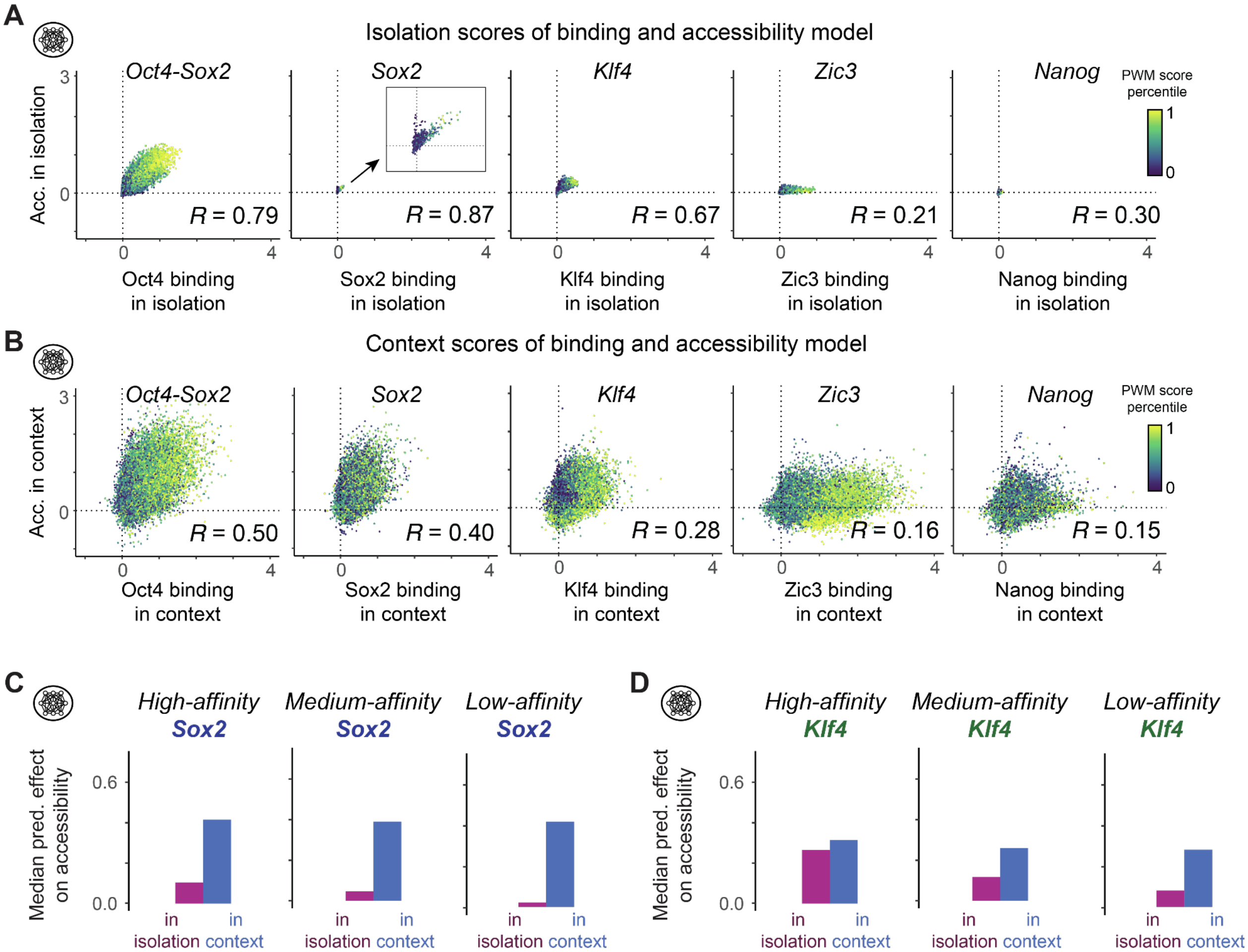

Figure S2. Isolation and context scores predicted by the accessibility model indicate that pioneering motifs are boosted by surrounding genomic sequence, related to Figure 2

A and B) Comparison of the **(A)** isolation scores or **(B)** context scores obtained from the binding model (x-axis) and the accessibility model (y-axis) for the *Oct4-Sox2*, *Sox2*, *Klf4, Zic3* and *Nanog* mapped motifs, colored by their PWM score percentiles.

C and D) For all sets of motifs motifs mapped by only accessibility models, median isolation and context scores predicted by the accessibility model for **(C)** *Sox2* motifs and **(D)** *Klf4* motifs of high, medium and low affinity motifs (5k each, based on PWM score) show that low-affinity motifs receive a bigger boost in context than high-affinity motifs.

Figure S3

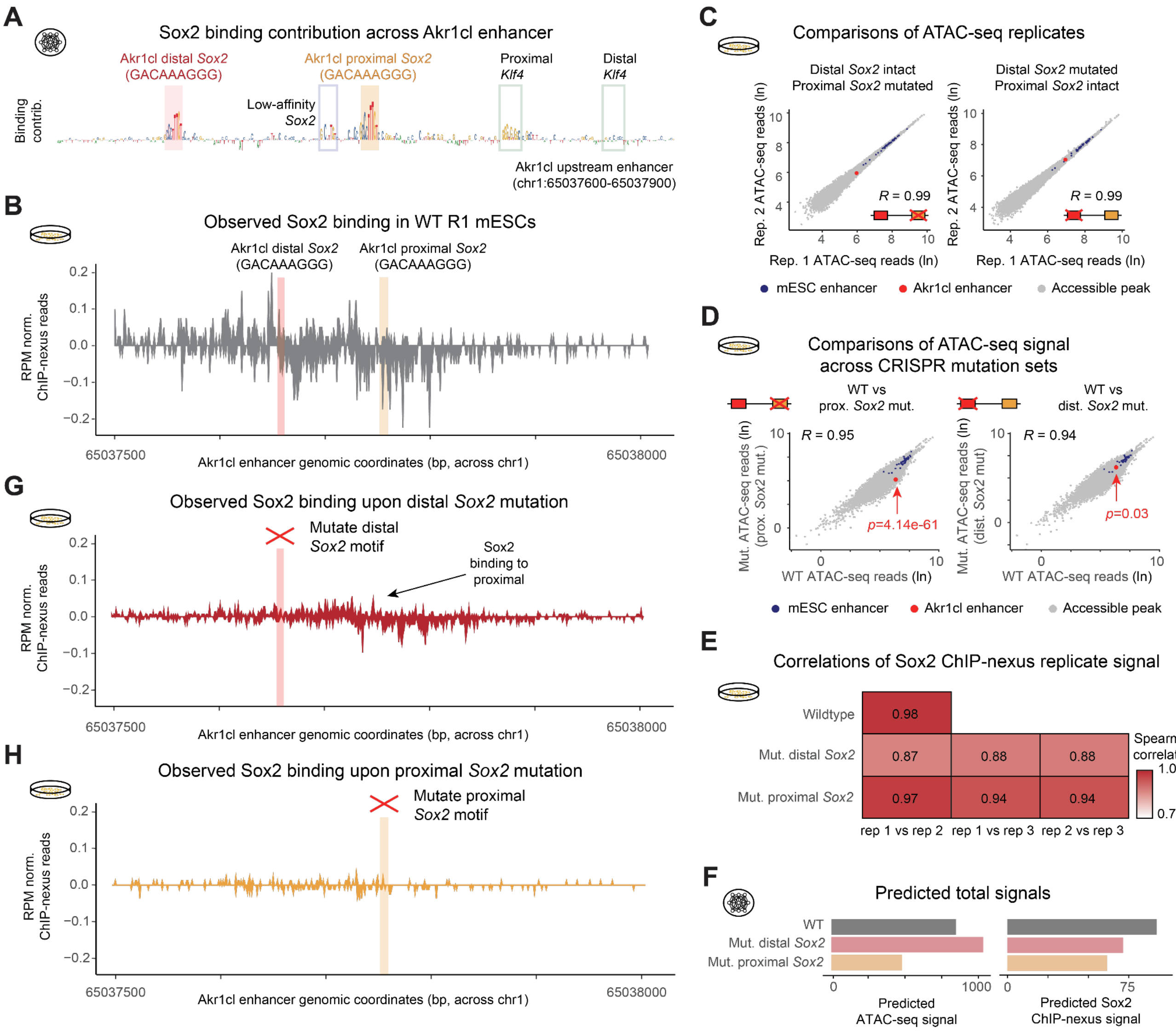

Figure S3. CRISPR mutant validation experiments confirm that motif placement within an enhancer influences its ability to bind and pioneer chromatin, related to Figure 3

A) Sox2 binding contribution scores of the Akr1cl upstream enhancer (chr1: 65037600-65037900) with contributing motifs marked.

B) RPM-normalized Sox2 ChIP-nexus across the Akr1cl enhancer, denoting distinct Sox2 binding profiles at both *Sox2* motifs.

C) Replicate comparisons measuring total ATAC-seq reads across reproducible ATAC-seq peaks from wildtype and CRISPR *Akr1cl* mutant experiments. Spearman correlation coefficients are reported on scatterplots.

D) Pairwise comparisons of pooled ATAC-seq reads from wildtype and CRISPR Akr1cl mutant experiments. Spearman correlation coefficients are reported on scatterplots. Differential accessibility between wildtype and each CRISPR Akr1cl mutant experiment was calculated using DESeq2, with adjusted significance values reported for the Akr1cl enhancer (red arrows).

E) Replicate Spearman correlations measuring total Sox2 ChIP-nexus reads across reproducible ATAC-seq peaks from CRISPR *Akr1cl* mutant experiments.

F) Predicted ATAC-seq signal or predicted Sox2 ChIP-nexus signal occurring across chr1: 65037600-65037900 across wildtype (black), distal *Sox2* mutant (red) and proximal *Sox2* mutant (orange) sequences.

G and H) RPM-normalized Sox2 ChIP-nexus profiles across the *Akr1cl* enhancer from **(G)** the distal *Sox2* mutation and the **(H)** the proximal *Sox2* mutation CRISPR experiments.

Figure S4

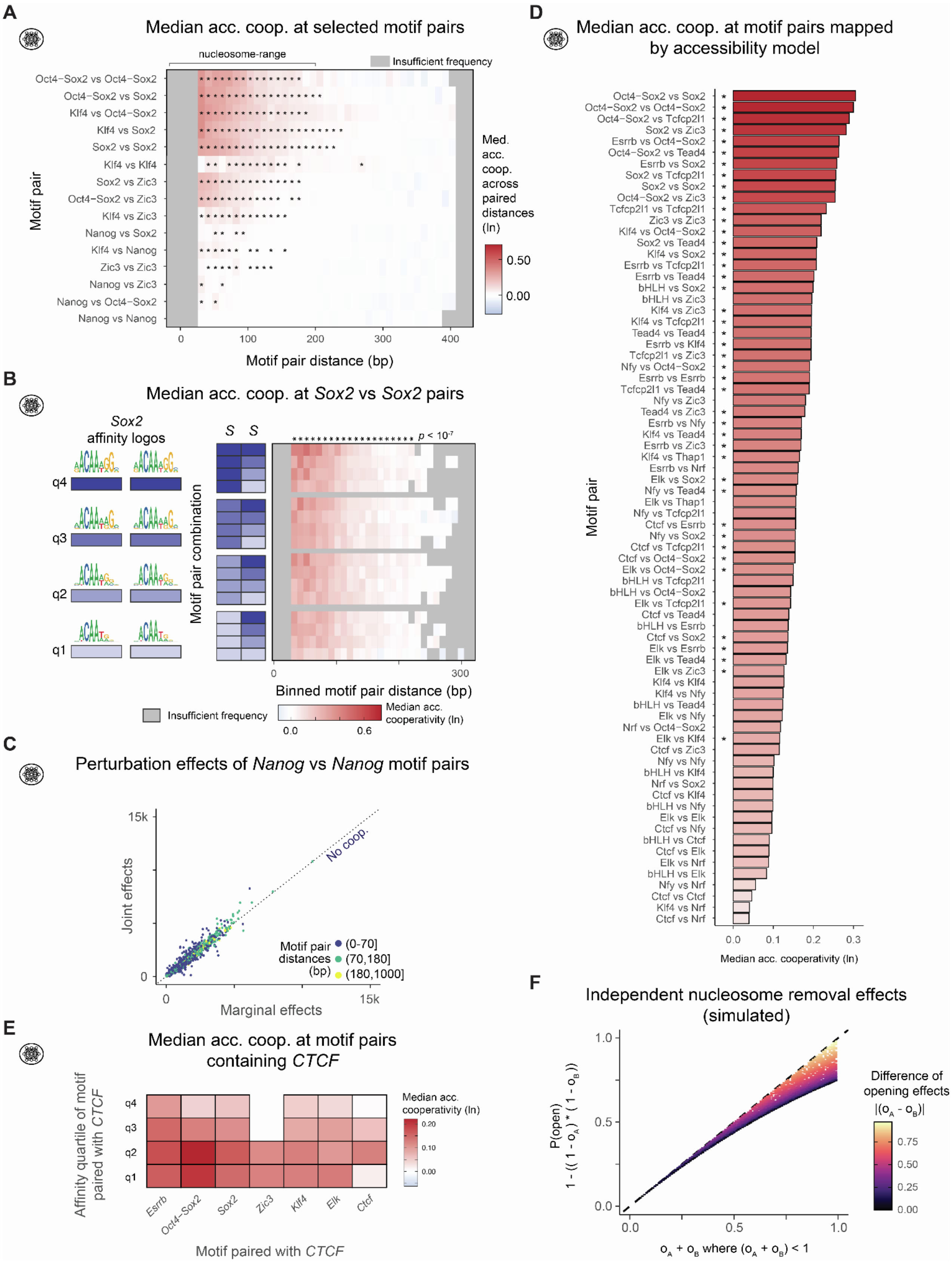
A
Median acc. coop. at selected motif pairs
nucleosome-range
Insufficient frequency
Oct4−Sox2 vs Oct4−Sox2
Oct4−Sox2 vs Sox2
Klf4 vs Oct4−Sox2
Klf4 vs Sox2
Sox2 vs Sox2
Klf4 vs Klf4
Sox2 vs Zic3
Oct4−Sox2 vs Zic3
Klf4 vs Zic3
Nanog vs Sox2
Klf4 vs Nanog
Zic3 vs Zic3
Nanog vs Zic3
Nanog vs Oct4−Sox2
Nanog vs Nanog
Motif pair
Med. acc. coop. across paired distances (ln)
0.50
0.00
0
100
200
300
400
Motif pair distance (bp)
B
Median acc. coop. at Sox2 vs Sox2 pairs
Sox2 affinity logos
q4
q3
q2
q1
S
S
Motif pair combination
p < 10^-7
0
300
Binned motif pair distance (bp)
Insufficient frequency
Median acc. cooperativity (ln)
0.0
0.6
C
Perturbation effects of Nanog vs Nanog motif pairs
15k
0
Joint effects
No coop.
Motif pair distances (bp)
(0-70]
(70,180]
(180,1000]
Marginal effects
D
Median acc. coop. at motif pairs mapped by accessibility model
Oct4−Sox2 vs Sox2
Oct4−Sox2 vs Oct4−Sox2
Oct4−Sox2 vs Tcfcp2l1
Sox2 vs Zic3
Esrrb vs Oct4−Sox2
Oct4−Sox2 vs Tead4
Esrrb vs Sox2
Sox2 vs Tcfcp2l1
Sox2 vs Sox2
Oct4−Sox2 vs Zic3
Tcfcp2l1 vs Tcfcp2l1
Zic3 vs Tcfcp2l1
Klf4 vs Oct4−Sox2
Sox2 vs Tead4
Klf4 vs Sox2
Esrrb vs Tcfcp2l1
Esrrb vs Tead4
bHLH vs Sox2
bHLH vs Zic3
Klf4 vs Zic3
Klf4 vs Tcfcp2l1
Tead4 vs Tead4
Esrrb vs Klf4
Tcfcp2l1 vs Zic3
Nfy vs Oct4−Sox2
Esrrb vs Esrrb
Tcfcp2l1 vs Tead4
Nfy vs Zic3
Tead4 vs Zic3
Esrrb vs Nfy
Klf4 vs Tead4
Esrrb vs Zic3
Klf4 vs Thap1
Esrrb vs Nrf
Elk vs Sox2
Nfy vs Tead4
Elk vs Thap1
Nfy vs Tcfcp2l1
Ctcf vs Esrrb
Nfy vs Sox2
Ctcf vs Tcfcp2l1
Ctcf vs Oct4−Sox2
Elk vs Oct4−Sox2
bHLH vs Tcfcp2l1
bHLH vs Oct4−Sox2
Elk vs Tcfcp2l1
Ctcf vs Tead4
bHLH vs Esrrb
Ctcf vs Sox2
Elk vs Esrrb
Elk vs Tead4
Elk vs Zic3
Klf4 vs Klf4
Klf4 vs Nfy
bHLH vs Tead4
Elk vs Nfy
Nrf vs Oct4−Sox2
Elk vs Klf4
Ctcf vs Zic3
Nfy vs Nfy
bHLH vs Klf4
Nrf vs Sox2
Ctcf vs Klf4
bHLH vs Nfy
Elk vs Elk
Ctcf vs Nfy
bHLH vs Ctcf
Ctcf vs Elk
Elk vs Nrf
bHLH vs Elk
Nfy vs Nrf
Ctcf vs Ctcf
Klf4 vs Nrf
Ctcf vs Nrf
Motif pair
0.0
0.1
0.2
0.3
Median acc. cooperativity (ln)
E
Median acc. coop. at motif pairs containing CTCF
Affinity quartile of motif paired with CTCF
q4
q3
q2
q1
Esrrb
Oct4-Sox2
Sox2
Zic3
Klf4
Elk
Ctcf
Motif paired with CTCF
Median acc. cooperativity (ln)
0.20
0.10
0.00
F
Independent nucleosome removal effects (simulated)
P(open)
1 - ((1 - o_A) * (1 - o_B))
1.0
0.5
0.0
0.0
0.5
1.0
o_A + o_B where (o_A + o_B) < 1
Difference of opening effects
|(o_A - o_B)|
0.75
0.50
0.25
0.00

Figure S4. Pioneering cooperativity occurs between most pioneer motifs depends on a distance-dependent soft motif syntax, related to Figure 4

A) Median accessibility cooperativity across all motif pairs of key mESC motifs, separated by motif pair distances. Motif/distance combinations with fewer than 20 motif pairs were not considered (gray boxes). Cooperativity significance was derived from a one-tailed Wilcoxon test comparing whether motif pairs arranged at select binned distances were more cooperative than motif pairs arranged at very long distances (>400 bp) with multiple-comparison Bonferroni corrections and an adjusted significance cutoff of $p < 10^{-7}$.

B) Median accessibility cooperativity between *Sox2*/*Sox2* motif pairs across quartiles of motif affinity and motif pair distances, binned to 10 bp. Motif/distance combinations with fewer than 20 motif pairs were not considered (gray boxes). Cooperativity significance was derived from a one-tailed Wilcoxon test comparing whether *Sox2*/*Sox2* motif pairs arranged at select binned distances were more cooperative than *Sox2*/*Sox2* motif pairs arranged at very long distances (>400 bp) with multiple-comparison Bonferroni corrections and an adjusted significance cutoff of $p < 10e^{-7}$.

C) Comparison of joint and marginal predicted accessibility effects for the non-pioneer *Nanog*/*Nanog* motif pairs, colored by the pairs' relative center-to-center distances to one another.

D) Median accessibility cooperativity across all cross-validated motif pairs found and mapped by the accessibility model (Figure S1C) occurring within 300 bp of one another, also colored by median accessibility cooperativity. Cooperativity significance was derived from a one-tailed Wilcoxon test comparing whether each combination of motif pairs were more cooperative than motif pairs of any combination arranged at very long distances (>400 bp) with multiple-comparison Bonferroni corrections and an adjusted significance cutoff of $p < 10e^{-7}$.

E) Median accessibility cooperativity between motif pairs mapped by the accessibility model that contain *CTCF* motifs, stratified across quartiles of motif affinities for the motifs paired with *CTCF*, but not *CTCF* itself. Motif pair combinations with fewer than 20 motif pairs were not considered.

F) Opening likelihoods for two pioneer motifs ($o_A = [0.01, 1]$ and $o_B = [0.01, 1]$ where $o_A + o_B < 1$) were independently sampled (n=10,000) and simulated based on the premise that each pioneer TF independently removes the same nucleosome with a certain probability ($P(open) = 1 - ((1 - o_A) * (1 - o_A)))$, producing less than additive effects.

Figure S5

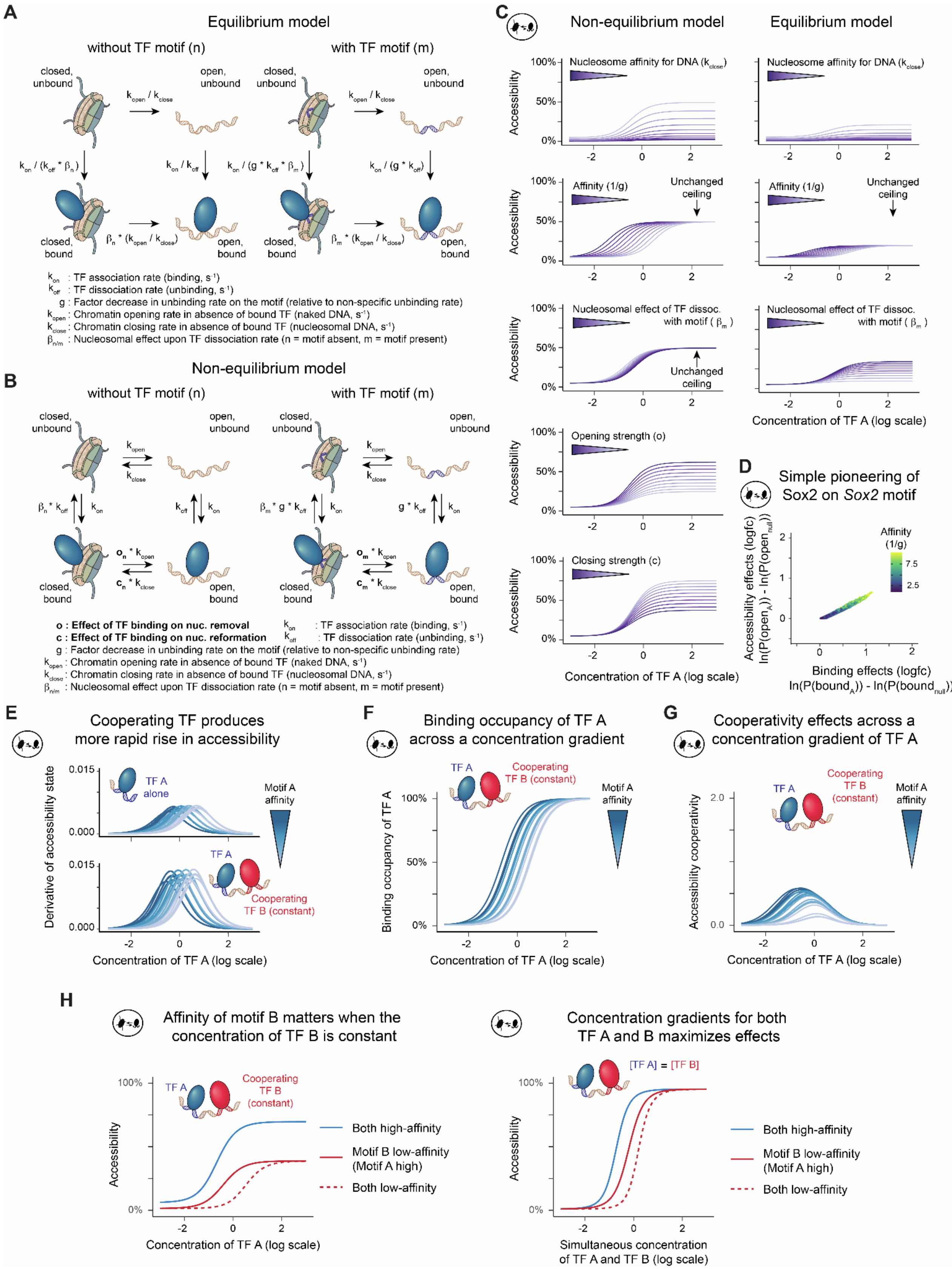

A
Equilibrium model
without TF motif (n)
with TF motif (m)
closed, unbound
open, unbound
closed, bound
open, bound
k_{on} : TF association rate (binding, s^{-1})
k_{off} : TF dissociation rate (unbinding, s^{-1})
g : Factor decrease in unbinding rate on the motif (relative to non-specific unbinding rate)
k_{open} : Chromatin opening rate in absence of bound TF (naked DNA, s^{-1})
k_{close} : Chromatin closing rate in absence of bound TF (nucleosomal DNA, s^{-1})
$\beta_{n/m}$: Nucleosomal effect upon TF dissociation rate (n = motif absent, m = motif present)
B
Non-equilibrium model
without TF motif (n)
with TF motif (m)
o : Effect of TF binding on nuc. removal
c : Effect of TF binding on nuc. reformation
C
Non-equilibrium model
Equilibrium model
Nucleosome affinity for DNA (k_{close})
Affinity (1/g)
Unchanged ceiling
Nucleosomal effect of TF dissoc. with motif (β_m)
Opening strength (o)
Closing strength (c)
Accessibility
Concentration of TF A (log scale)
D
Simple pioneering of Sox2 on *Sox2* motif
Affinity (1/g)
Accessibility effects (logfc) ln(P(open$_A$)) - ln(P(open$_{null}$))
Binding effects (logfc) ln(P(bound$_A$)) - ln(P(bound$_{null}$))
E
Cooperating TF produces more rapid rise in accessibility
TF A alone
Motif A affinity
Cooperating TF B (constant)
Derivative of accessibility state
Concentration of TF A (log scale)
F
Binding occupancy of TF A across a concentration gradient
Binding occupancy of TF A
G
Cooperativity effects across a concentration gradient of TF A
Accessibility cooperativity
H
Affinity of motif B matters when the concentration of TF B is constant
Both high-affinity
Motif B low-affinity (Motif A high)
Both low-affinity
Concentration gradients for both TF A and B maximizes effects
[TF A] = [TF B]
Simultaneous concentration of TF A and TF B (log scale)

Figure S5. Kinetic modeling of nucleosome-mediated TF cooperativity shows that pioneer cooperativity extends the regulatory potential of a motif, related to Figure 5

A) Parameterized graphic depicting a kinetic model in equilibrium of bound TF effects on nucleosome removal and reformation. Transitions are reversible, but they are only shown in one direction given that the equilibrium steady state is only determined by the ratios between forward and backward rates, as indicated. On the left, the motif is not present and therefore recruits only non-specific binding to the TF. On the right, the motif is present and recruits specific TF binding, with motif affinity effectively given by the factor 1/*g*.

B) Parameterized graphic depicting a kinetic model out of equilibrium of bound TF effects on nucleosome removal and reformation. Parameter definitions are the same from graphical representation in **(A)**. Additional non-equilibrium parameters include *o* (effect of bound TF on nucleosome removal) and *c* (effect of bound TF on nucleosome reformation. Note that $c \leq 1$, such that a higher value of c means less of an effect).

C) 1 TF kinetic model simulations in equilibrium (right) and non-equilibrium (left) showing the accessibility state over changing concentrations for a range of changing model parameters. We tested ranges of nucleosome intrinsic affinity for DNA by varying k_{close}, motif affinity, nucleosome effect upon TF dissociation in the presence of a motif, and in the case of the non-equilibrium model, TF binding effects on nucleosome removal (opening strength, *o*) and reformation (closing strength, *c*). For the equilibrium model, the parameters were set to simulate moderate pioneering in the presence of a nucleosome with low intrinsic affinity to DNA. Parameter ranges can be found in Table S7 (equilibrium model) and Table S8 (non-equilibrium model).

D) 1 TF kinetic model (out of equilibrium) simulations (n=50,000) showing the correlation between steady-state accessibility state, binding occupancy and motif affinity in the presence of a pioneer TF. Parameter ranges can be found in Table S8. Note that $c = 1$ (effect of TF on nucleosome reformation), indicating that the simulated simple pioneering does not allow reduction of nucleosome reformation as a mechanism to increase accessibility, only nucleosome removal.

E-G) Expansion of the 1 TF kinetic model from Figure 5B to the 2 TF kinetic model from Figure 5E over a range of TF A motif affinities across changing concentrations of TF A. TF B affinity and concentration was kept constant and parameter ranges can be found in Table S9. **E)** Accessibility curve derivatives show that the cooperative (2 TF) conformation produces a more rapid rise in accessibility.

F) Binding occupancy curves show that as concentration of TF A increases, binding occupancy reaches the maximum value of 100%, unlike accessibility state which plateaus at values less than 100% (Figure 5E).

G) Accessibility cooperativity shows that though TF B affinity and concentration was kept constant, the presence of B caused accessibility states to be cooperatively enhanced. Note that at high TF A concentrations, increased non-specific TF binding decreases the cooperative influence of motifs, particularly low-affinity motifs. Because high-affinity motifs are able to bind more specifically at lower concentrations, maximum cooperativity is increased with affinity.

H) 2 TF kinetic model simulations measuring the accessibility state, measured as steady-state probability of the open conformation, upon two pioneer TFs binding when TF A and TF B motif affinities are both high (blue solid line), are both low (red dashed line) and when TF A is high-affinity and TF B is low-affinity (red solid line). These affinity comparisons are conducted when TF A concentrations are allowed to change but TF B concentrations are not (left panel) and when both TF A and TF B concentrations are allowed to change at the same rate (right panel). TF A and TF B were modeled from Sox2 binding dynamics *in vivo* with the same parameters as **(B)**, excluding motif affinity. Parameter ranges can be found in Table S9. For **C,E-H)**, concentrations are in units of 10*nM.

Figure S6

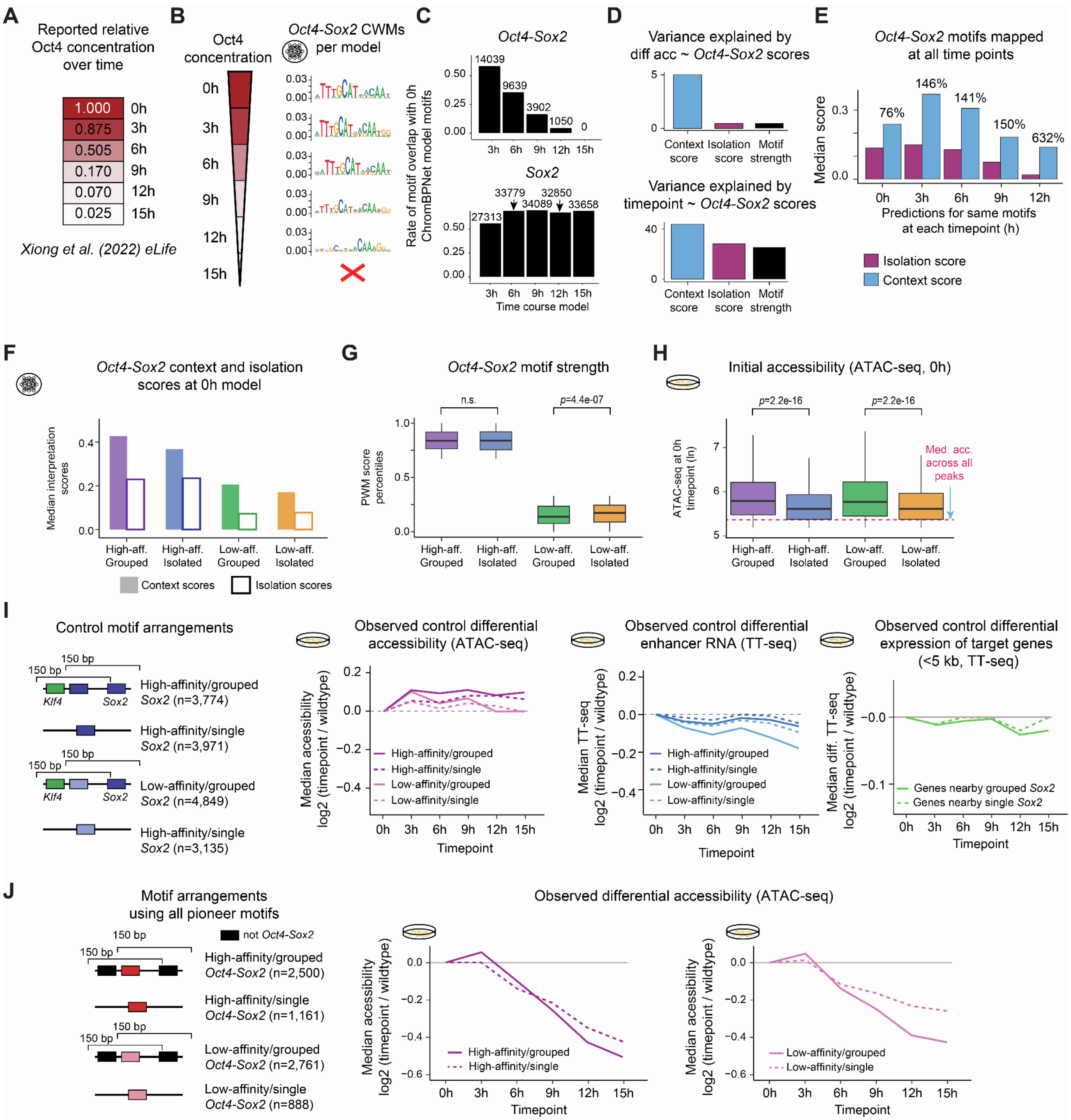
A
Reported relative Oct4 concentration over time
1.000 0h
0.875 3h
0.505 6h
0.170 9h
0.070 12h
0.025 15h
Xiong et al. (2022) eLife
B
Oct4 concentration
0h
3h
6h
9h
12h
15h
Oct4-Sox2 CWMs per model
C
Oct4-Sox2
14039
9639
3902
1050
0
3h 6h 9h 12h 15h
Sox2
27313
33779
34089
32850
33658
3h 6h 9h 12h 15h
Time course model
Rate of motif overlap with 0h ChromBPNet model motifs
D
Variance explained by diff acc ~ Oct4-Sox2 scores
Context score
Isolation score
Motif strength
Variance explained by timepoint ~ Oct4-Sox2 scores
E
Oct4-Sox2 motifs mapped at all time points
76%
146%
141%
150%
632%
Median score
0h 3h 6h 9h 12h
Predictions for same motifs at each timepoint (h)
Isolation score
Context score
F
Oct4-Sox2 context and isolation scores at 0h model
Median interpretation scores
High-aff. Grouped
High-aff. Isolated
Low-aff. Grouped
Low-aff. Isolated
Context scores
Isolation scores
G
Oct4-Sox2 motif strength
n.s.
p=4.4e-07
PWM score percentiles
H
Initial accessibility (ATAC-seq, 0h)
p=2.2e-16
p=2.2e-16
Med. acc. across all peaks
ATAC-seq at 0h timepoint (ln)
I
Control motif arrangements
150 bp
Klf4
Sox2
High-affinity/grouped Sox2 (n=3,774)
High-affinity/single Sox2 (n=3,971)
Low-affinity/grouped Sox2 (n=4,849)
High-affinity/single Sox2 (n=3,135)
Observed control differential accessibility (ATAC-seq)
Median accessibility log2 (timepoint / wildtype)
High-affinity/grouped
High-affinity/single
Low-affinity/grouped
Low-affinity/single
Timepoint
Observed control differential enhancer RNA (TT-seq)
Median TT-seq log2 (timepoint / wildtype)
Observed control differential expression of target genes (<5 kb, TT-seq)
Median diff. TT-seq log2 (timepoint / wildtype)
Genes nearby grouped Sox2
Genes nearby single Sox2
0h 3h 6h 9h 12h 15h
J
Motif arrangements using all pioneer motifs
not Oct4-Sox2
150 bp
High-affinity/grouped Oct4-Sox2 (n=2,500)
High-affinity/single Oct4-Sox2 (n=1,161)
Low-affinity/grouped Oct4-Sox2 (n=2,761)
Low-affinity/single Oct4-Sox2 (n=888)
Observed differential accessibility (ATAC-seq)
Median accessibility log2 (timepoint / wildtype)
High-affinity/grouped
High-affinity/single
Low-affinity/grouped
Low-affinity/single
Timepoint

Figure S6. Pioneer cooperativity sensitizes an enhancer to changing TF concentrations *in vivo*, related to Figure 6
A) Reported relative Oct4 protein levels upon doxycycline withdrawal time course.
B) Accessibility TF-MoDISco CWMs of discovered *Oct4-Sox2* motifs from accessibility models of all Oct4 concentration time points. Accessibility model capturing the 15h time point failed to discover a contributing role of the *Oct4-Sox2* motif to accessibility.
C) Rates by which *Oct4-Sox2* and *Sox2* motifs mapped by the reference 0h time point model were mapped by the corresponding time course accessibility models, with the number of mapped motifs shown above each rate.
D) Independent linear regression was used to determine the variance that each *Oct4-Sox2* motif score explains of the observed differential accessibility changes over the concentration time course (top) or the time point (treated as numeric value) at which each *Oct4-Sox2* motif was last mapped (bottom). Context scores explain more variance, showing that context matters beyond motif affinity.
E) For the same *Oct4-Sox2* motifs mapped throughout the time course until 12h, the median context and isolation scores were calculated for each time point. While the scores decrease with decreasing Oct4 concentrations as expected, the context scores become larger relative to the isolation scores (shown as % increase on top). Given that the motifs and concentration of the other TFs remain constant, this shows that weak motifs are contextually more enhanced.
(F-J) Selection of single and group motif arrangements from accessible regions (above the 60th percentile of total accessibility at 0h). **F)** Median context and isolation scores of *Oct4-Sox2* motifs mapped by the 0h time point model. While both grouped and single motif arrangements possess higher context scores than isolation scores (likely because grouped arrangements only consider *Sox2* and *Klf4* motif partners and not other motifs), grouped arrangements possess a higher relative ratio of the two interpretation scores.
G) Boxplots depicting PWM score percentiles of *Oct4-Sox2* motifs across the motif arrangements denoted in Figure 6E. Statistics were performed using a Wilcoxon test.
H) Boxplots depicting initial ATAC-seq reads at the 0h time point across the motif arrangements denoted in Figure 6E. For reference, median accessibility across all accessible regions used for time course ChromBPNet model training is projected as a dashed blue line. Statistics were performed using a Wilcoxon test. Note that grouped motif arrangements possess intrinsically higher initial accessibility levels than single motif arrangements.
I) Control arrangements of either single (dashed line) or grouped (solid line) high- and low-affinity *Sox2* motifs without an *Oct4-Sox2* motif nearby (graphic on the left) show little changes in median differential levels for ATAC-seq accessibility (pink), TT-seq enhancer RNA (blue), and TT-seq nearest target gene expression (green) over the concentration time course. For accessibility (pink) and enhancer RNA (blue) arrangements, high- and low-affinity *Sox2* motifs were the top and bottom tertiles of PWM scores. For target gene expression measurements (green), target genes were considered if only a single *Sox2*-containing region was within 100 kb, the nearest target gene start was within 5 kb of a *Sox2*-containing region, and this *Sox2*-containing region contained either a single (solid line) or grouped (dashed line) *Sox2* motif without an *Oct4-Sox2* nearby, with all considered motifs derived from the 0h time point model.
J) Motif arrangements (shown as graphics on the left) with a high-affinity *Oct4-Sox2* motif (middle) or low-affinity *Oct4-Sox2* motif (right) either single (dashed line) or in a grouped arrangement (solid line) with any pioneer motif found by the accessibility model within 150 bp (Figure S1C) show distinct median experimental differential accessibility levels over the concentration time course. *Oct4-Sox2* motifs were derived from the 0h time point model, and high- and low-affinity motifs were classified as the top and bottom tertiles of measured PWM scores. Note that grouped arrangements show a steeper loss of accessibility.

Table S11. Oligonucleotides used for ChIP-nexus, related to STAR Methods.

Name	Identity	Modification	Barcode	Sequence
Nex_adapter_U	Adaptor: universal	5' phosphate	/	/5Phos/GATCGGAAGAGCACACGTCTGGATCCACGACGCTCTTCC
Nex_adapter_1	Adaptor: barcoded	5' phosphate	AGTCNNNNN	/5Phos/AGTCNNNNNAGATCGGAAGAGCGTCGTGGATCCAGACGTGTGCTCTTCCGATCT
Nex_adapter_2	Adaptor: barcoded	5' phosphate	CAGTNNNNN	/5Phos/CAGTNNNNNAGATCGGAAGAGCGTCGTGGATCCAGACGTGTGCTCTTCCGATCT
Nex_adapter_3	Adaptor: barcoded	5' phosphate	GTCANNNNN	/5Phos/GTCANNNNNAGATCGGAAGAGCGTCGTGGATCCAGACGTGTGCTCTTCCGATCT
Nex_adapter_4	Adaptor: barcoded	5' phosphate	TCAGNNNNN	/5Phos/TCAGNNNNNAGATCGGAAGAGCGTCGTGGATCCAGACGTGTGCTCTTCCGATCT
Nex_primer_U	Primer: universal	3' phosphoro-thioate bond	/	AATGATACGGCGACCACCGAGATCTACACTCTTTCCCTACACGACGCTCTTCCGATC*T
Nex_primer_01	Primer: indexed	3' phosphoro-thioate bond	ATCACG	CAAGCAGAAGACGGCATACGAGATCGTGATGTGACTGGAGTTCAGACGTGTGCTCTTCCGATC*T
Nex_primer_02	Primer: indexed	3' phosphoro-thioate bond	CGATGT	CAAGCAGAAGACGGCATACGAGATACATCGGTGACTGGAGTTCAGACGTGTGCTCTTCCGATC*T
Nex_primer_03	Primer: indexed	3' phosphoro-thioate bond	TTAGGC	CAAGCAGAAGACGGCATACGAGATGCCTAAGTGACTGGAGTTCAGACGTGTGCTCTTCCGATC*T
Nex_primer_04	Primer: indexed	3' phosphoro-thioate bond	TGACCA	CAAGCAGAAGACGGCATACGAGATTGGTCAGTGACTGGAGTTCAGACGTGTGCTCTTCCGATC*T
Nex_primer_05	Primer: indexed	3' phosphoro-thioate bond	ACAGTG	CAAGCAGAAGACGGCATACGAGATCACTGTGTGACTGGAGTTCAGACGTGTGCTCTTCCGATC*T
Nex_primer_06	Primer: indexed	3' phosphoro-thioate bond	GCCAAT	CAAGCAGAAGACGGCATACGAGATATTGGCGTGACTGGAGTTCAGACGTGTGCTCTTCCGATC*T
Nex_primer_07	Primer: indexed	3' phosphoro-thioate bond	CAGATC	CAAGCAGAAGACGGCATACGAGATGATCTGGTGACTGGAGTTCAGACGTGTGCTCTTCCGATC*T
Nex_primer_08	Primer: indexed	3' phosphoro-thioate bond	ACTTGA	CAAGCAGAAGACGGCATACGAGATTCAAGTGTGACTGGAGTTCAGACGTGTGCTCTTCCGATC*T

ChIP-nexus adapters are generated using the universal adapter (Nex_adapter_U) and the barcoded adapters (Nex_adapter_1, Nex_adapter_2, Nex_adapter_3, Nex_adapter_4). The universal (Nex_primer_U) and indexed (Nex_primer_01 - Nex_primer_08) primers are used for amplification of the library. Oligonucleotides can be ordered from Integrated DNA Technologies (IDT).
