# Supplementary material for: Cooperativity enables widespread role of low-affinity motifs in chromatin accessibility and increases regulatory potential
Source: Cell Genom. 2026 Aug 25;6(10):101339. doi: 10.1016/j.xgen.2026.101339 (PMC13689504; doi:10.1016/j.xgen.2026.101339)
Supplement: Supplementary file 2 [file mmc12.pdf]

Cell Genomics Transparent Peer Review Record

Cooperativity enables widespread role of lowaffinity motifs in chromatin accessibility and increases regulatory potential

Author list: Melanie Weilert, Kaelan J. Brennan, Khyati Dalal, Sabrina Krueger, Haining Jiang, Rosa Martinez-Corral & Julia Zeitlinger

Summary

Initial submission:	Received : December 5th 2025
	Scientific editor: Judith Nicholson
First round of review:	Number of reviewers: 3<br>Revision invited : January 28th 2026<br>Revision received : June 10th 2026
Second round of review:	Number of reviewers: 3<br>Accepted : August 4th 2026
Data freely available:	Yes
Code freely available:	Yes

This transparent peer review record is not systematically proofread, type-set, or edited. Special characters, formatting, and equations may fail to render properly. Standard procedural text within the editor's letters has been deleted for the sake of brevity, but all official correspondence specific to the manuscript has been preserved.

Referees' reports, first round of review

Reviewer 1:

Weilert et al. used a deep learning-based approach in combination with genetic perturbation experiments in mESCs to systematically map and assess the contribution of low-affinity TF-binding sites to DNA accessibility. This is a significant question in the field, since previous studies showed the importance of low-affinity TF-binding sites at selected loci for enhancer specificity in vivo (e.g., Farley, Levine, Crocker, Stern labs) and in vitro (e.g., Bulyk lab). However, their general importance for enhancer activity and their role in opening chromatin remained unclear.

The authors trained a state-of-the-art BPNet model on ChIP-nexus data to predict TF binding for 5 pioneer factors (Oct4, Sox2, Nanog, Klf4, and Zic3) and ChromBPNet on ATAC-seq data to predict DNA accessibility. The authors then systematically mapped contributing TF motifs and showed that even low-affinity motifs, defined by low PWM scores, significantly contribute to the model. Importantly, they performed experimental validation of model predictions by mutating both high- and low-affinity pioneer TF-binding sites, confirming their contribution to endogenous DNA accessibility (Fig. 1G,H; 3E,F and 4A). They further demonstrated that, especially for low-affinity motifs, the context in which the motif appears is crucial for pioneering activity. While the median predicted effect on accessibility is low for low-affinity motifs when surrounded by random sequence, it increases significantly when these motifs are present alongside other motifs in a genomic context. Based on these results and supported by modeling, the authors conclude that low-affinity motifs cooperate with neighboring motifs to increase accessibility.

Overall, this is an important study that clearly shows the general importance of previously overlooked low-affinity TF-binding sites for DNA accessibility genome-wide for several factors. That said, there are concerns about study design and data interpretation that should be addressed before publication. Most notably, the authors introduce a systematic bias in a study design by using a training set with heavily overrepresented accessible chromatin regions and focusing on the SOX2-OCT4 motif, which is already cooperative, undermining claims about context and potentially missing important regulatory logic. Moreover, the

definition of low affinity is ambiguous. Finally, the data presented cannot rule out a simple additive model in which each TF contributes additively toward general accessibility. These and other comments are listed below:

Major Comments:

1. The training set, which includes ~150,000 1-kb open chromatin regions, is heavily biased toward accessible regions, which may bias model findings. This bias may cause the model to overtrain on contextual scores within open chromatin, thereby missing features of repression. They should clarify the rationale for limiting the analysis to 154,827 1-kb intervals. Do the authors observe the same findings if the model is trained on the entire genome? These comparisons should be included in the revised manuscript.

2. It is unclear how "low-affinity" is defined. This is an important consideration, as studies on low-affinity motifs will be compared, and it would be helpful to know whether the reported low affinities are actually comparable.

o If it's based solely on the match score to the PWM, did the authors use consistent cutoffs across different TFs?

o Are these cutoffs similar to those reported for low-affinity TFBS in functional enhancers (Crocker et al., 2015; Farley et al., 2015; Lim et al., 2024, etc.)?

o Fig 1G: Are neighboring Sox2 motifs high or low-affinity? Please state the cutoff thresholds if yes.

2. The authors claim that pioneering cooperativity is the primary mechanism by which low-affinity TF motifs contribute to accessibility. However, a simpler additive contribution, in which each motif contributes a little bit, cannot be excluded. Moreover, their experimental data support an additive model rather than cooperativity. First, the effects of low-affinity motif deletions (Fig. 1H and 4B) seem to be rather mild, which is more consistent with an additive model. Second, the result of the combined deletions in 4B shows a marginally smaller effect than a simple sum of individual deletions, indicating that it's mostly additive, but the authors interpret it as "cooperativity". Third, in Fig. 3D, they delete only strong Sox2 motifs, not the weak ones, and the results further support an additive model (3G,H). The authors should either remove the cooperativity conclusion, for which they don't have experimental validation, or call it context-sensitivity or context-specificity, which is more accurate given the data presented.

3. The authors focus their analysis and experiments on the composite OCT4-

SOX2 motif, treating it as a binding site for a single TF, which introduces some confusion and complicates interpretations. Specifically:

o The OCT4-SOX2 motif does not appear representative of other pioneer TF binding motifs, which do not directly cooperate. Are the same findings true for more general pioneers?

o How exactly is the OCT4-SOX2 affinity defined? It often appears that the OCT4 and SOX2 motif matches degrade independently (Fig 4D, Fig 6B, etc.). In Fig 1E, the bottom mapping, presumed to be low affinity, contains a perfect OCT4 motif and a degenerate SOX2 motif. It would be helpful to analyze these motifs separately.

o While SOX2 is examined independently, OCT4 is not (Fig1C, Fig4, and Fig6). The same analysis should be presented for OCT4 as well.

o OCT-SOX composite motif is an inherently cooperative interaction, which makes comparisons to other motifs confusing - is it a three-way cooperativity?

4. Fig 1F: This result is not surprising, given that only a single-digit percentage of all motif occurrences in the genome are bound by a given pioneer TF. A more rigorous control would be to score PWMs within already accessible peaks (since ATAC-seq data exist). Is ChromBPnet better at mapping TFBS than simply overlapping PWMs with ATAC-seq/ChIP peaks?

5. Fig 6: Potential biases in comparing group versus single motif arrangements. How is affinity managed across groups, such as a high-affinity motif group versus low-affinity ones, or vice versa? The authors should account for the potential confounding effects of motif-rich versus motif-poor regions. Perhaps compare grouped versus single motif arrangements of Oct4-Sox2 motif combined with non-ESC or non-Pioneering TF motifs.

6. Fig 6F/G: Are low-affinity/single motifs accessible at all (only fold-changes shown)?

7. Fig 1H: Provide a quantitative comparison (normalized for read density) as it is done in Fig 3G, H

8. After Fig 1, it is not clear which model is used throughout the text. Is it BPnet, chromBPnet, both? Please clarify.

9. Provide a detailed explanation of isolation and context score derivation in the main text (not just figure legends) since these are novel key definitions. Also how are isolation/context scores calculated when the same motifs occur multiple times within a short stretch? Are these discarded?

10. Provide more detail on the overlap between ChIP-nexus and ATAC-seq peaks (unique vs. shared Venn diagram).
11. Fig 3: Define "strongest pioneer motif". Is this based on motif score or accessibility contribution?
12. Fig 6E has an incorrect label (bottom should be "low-affinity/single")
13. Fig 4E: (0-70] then "(70,180]" is confusing. Please make labels consistent.

Reviewer 2:

The study 'Widespread low-affinity motifs enhance chromatin accessibility and regulatory potential in mESCs' from Weilert et al. trains deep learning models on DNA accessibility and ChIP-nexus TF occupancy data to investigate how the combinatorial presence of TF motifs (enhancer context) shapes enhancer accessibility and its sensitivity to TF concentration in mESCs. They show that using the model's contribution scores improves motif mapping by enhancing the specificity with which lower affinity motifs are mapped (based on ChIP-nexus data) compared to using PWM scoring alone. Based on this mapping, they investigate how combinations of pioneer motifs modulate enhancer accessibility in silico, finding widespread cooperativity, by which pioneer motif combinations within nucleosome-range distances lead to more accessibility as the combination of their individual contributions would suggest. The authors provide a kinetic non-equilibrium model for this behavior, which shows that motif cooperativity leads to an increase in the fraction of accessible enhancers and a more rapid rise in opening as a function of TF concentration. Using time-course data measuring DNA accessibility upon degradation of Oct4, they show that Oct4-Sox2 dimer motifs that occur in cooperative contexts (multiple pioneer motifs) lead to a steeper and higher magnitude drop in enhancer accessibility and RNA-expression compared Oct4-Sox2 motifs in isolation and that this effect occurs for both high and low affinity sites. This suggests that motif combinations heighten an enhancer's sensitivity to changing TF concentrations, a finding in line with motif cooperativity.

The study addresses an important outstanding question in the field of regulatory genomics, namely how a combinatorial TF code contributes to gene expression control. It outlines how pioneer nucleosome-range cooperativity may contribute to fine-tuning enhancer responsiveness to changing TF concentrations and how low affinity sites may contribute to this phenomenon. This finding provides an

interesting angle on non-canonical (not directly mediated through DNA binding) TF cooperativity and how it modulates enhancer activities and ultimately gene expression control and should thus be of interest to a broad readership. The strongest point of the study is the use of time-course experiments that show that enhancers with multiple pioneer motifs lead to a more rapid and higher magnitude change in overall accessibility as a function of decreasing TF concentration for a single TF (Oct4), which supports the cooperativity model. A weak point of the study is that the modeling on cooperativity is solely based on in-silico predictions and is thus entirely depended on the model's ability to learn cooperativity in the first place, which may or may not be the case. While there are some CRISPR experiments, the generated cell lines do not explicitly test for the presence of motif cooperativity, i.e. mutating one or the other or both pioneering motifs within proximity and comparing accessibility levels to confirm cooperativity.

Another emphasized aspect is the importance of low affinity binding sites, however, after reading the study one is left with the impression that different affinity sites are essentially interchangeable, and low affinity sites are simply used because they are easier to generate. This contradicts previous work showing that turning low affinity sites into higher affinity ones can have detrimental consequences.

In summary, the study would benefit from providing hard experimental evidence for TF motif cooperativity and a clear delineation of how low-affinity sites contribute to an optimal enhancer response curve (i.e. exploring how combinations of different affinity motifs alter enhancer accessibility in the kinetic model).

Main points:

- In the text it says that BPNet models were trained independently of one another, however, from the methods it reads as if the regions for training the accessibility model were pre-selected based on information of the ChIP-nexus data, thus informing the accessibility model of true TF occupancy. How would the results change if this was not the case, could this influence what motif combinations can effectively be learned in the accessibility model?

- Experiments in Figure 3 demonstrate that some motifs (perhaps because they are near other pioneer motifs) lead to larger accessibility changes, but it does not explicitly test whether TF-A and TF-B act cooperatively. There are other

explanations, like positioning of the motif with respect to the preferred nucleosome location. What happens if the low-affinity motif is deleted independently? How does accessibility change when the proximal or the low-affinity Sox2 motif are mutated in isolation, and is the sum of those less than the total accessibility measured in the native enhancer? Demonstrating at least for one case that the in silico predictions of cooperativity (green bars in subsequent plots) are real, would make this study much stronger as in its current form it hinges on the model's ability to correctly learn cooperativity, which was never experimentally validated.

- For the kinetic model it would help to illustrate the boundaries, e.g. what parameters are required to achieve 100% accessibility and how does this depend on the affinity of both TFs (varying both TF-A and TF-B). Can the authors provide an illustration of how different affinities alter the kinetic parameters and which parameter ranges (and motif affinities) are within physiologically relevant ranges? This gets to the point of how low and high affinity sites differ. They clearly are not interchangeable, as low affinity motifs operate at different TF concentrations and also lead to less cooperativity (SF5g). Reading that high and low sites are essentially interchangeable and that low affinity sites are not there due to their affinity but rather of how easy they are to make is quite confusing. If it's so simple, wouldn't that put developmental enhancers at constant risk of overshooting their activity levels? Besides, other studies have shown that high affinity sites can overshoot the target range, so affinity clearly is relevant.

- The data in Figure 6 is strong, however there is again no direct comparison of low and high affinity sites. It looks like high affinity has an impact on the magnitude of the change? Also, how comparable are the enhancers in terms of their affinity, i.e. are the grouped and single motif enhancers matched in their affinities (no statistically significant difference) ?

- Could the authors comment on the fact that they do not observe cooperativity with non-pioneer motifs. Is this because of the constraints placed on the training regions (selecting against certain motif pairs), or could this have to do with what the model is capable of learning?

* Can the authors elaborate in their modelling how non-pioneers would behave? I assume they should contribute to preventing nucleosomes from returning? Why would this not lead to cooperativity as has previously postulated for nucleosome-mediated cooperativity?

* There was another recent study that looked at accessibility changes at SNPs that if I recall correctly found non-pioneer motifs enriched in the context when

outsized accessibility changes occurred (PMID: 39363017). This seems somewhat contradicting, but might have to do with the differences in study design (SNPs as perturbations versus AI model), however it would be good to discuss how the study setup might influence the results.

* This goes back to the first point. Was the accessibility model trained only on regions with ChIP-nexus confirmed binding of a select number of TFs? Do those regions differ in their motif composition from the accessible peaks not chosen? How would this affect the results? Does this lead to (as the author's state in their limitation) 'out-of-distribution' sampling?

Minor:

- Not the best choice of title. It suggests that low-affinity sites are responsible for altering regulatory potential when in fact the study finds that this is not specific to low-affinity sites but rather due to combinatorial motif presence, so why not say that motif cooperativity is the main driver of altered regulatory potential? The regulatory amplitude (Fig 6) is in fact larger for high-affinity sites, so I'm not sure why the title emphasizes low-affinity sites? Similarly, the CRISPR validation experiments target high-affinity sites.

- At times it is confusing whether comparisons use in-silico predictions of accessibility and binding or the actual experimental data. A consistent use of 'predicted' or some other proxy would help.

Reviewer 3:

The authors present a study on low-affinity binding sites, elegantly combining deep learning model predictions and motif interpretation with targeted experiments and thermodynamic modeling. They show the importance of low-affinity binding sites using mouse embryonic stem cells as a model (mESCs), and as such convey an important conceptual advance. The authors start very broadly, stating that they would study pioneer transcription factors, defining pioneer TFs as any TF that has a measurable effect on chromatin accessibility, this is a great approach. They also present the unsolved issue of understanding "mechanisms by which enhancers function in vivo", this is an important question to address. They state the issue of lack of interpretation of deep-learning sequence-to-function models, this is indeed true. They start off with a global analysis of BPNet predictions of chromatin accessibility in mESCs and get all the way to thermodynamic modeling of TF binding based on these models. This is impressive.

Thus, overall they study asks all the right questions and make the right choice of global analyses. Unfortunately, they then don't fully deliver on their promise of unbiased global analysis and restricted their analysis a set of 5 very well-known TFs in mESCs. Thus, I have four major comments:

1) When the authors state "We then calculated the in silico cooperativity scores for all motif pairs across the genome[…] 4C" - are these all the motif pairs that were found? Or all the motif pairs of the 5 TFs that were analysed in more detail? Can they do this globally across all motifs?

2) Given the powerful pipeline that the authors present about BPNet predictions, deep understanding of motifs and cooperativity, I would have expected the authors to investigate some of the more functional effects on cooperative binding - e.g. on enhancer function in terms of expression. The authors specifically choose a very well-studied system (mESCs), so they could make use of these data that have been collected for the cell line (or produce their own).

3) Currently, the study is lacking any prediction of how all these CRE-grammar and cooperativity actually matter. For example, can the authors now predict how cells would adapt to changes and how cooperative binding or low-affinity motifs may be important for cellular function or differentiation? mESC are a great model for looking at differentiation. Along this line, one suggestion is to make use of data from a recent study by Bunina et al (https://doi.org/10.1016/j.cels.2020.05.003) - that has investigated Sox2 cooperative binding in mESCs differentiating to neurons and collected ATAC (as well as RNA and proteomic) data along a differentiation time course. This study suggests that Sox2 cooperatively binds with Oct4 as long as Oct4 is expressed and starts partnering with other TFs (such as Atrx) upon differentiation. The model the authors present would be a perfect tool to mechanistically investigate some of these aspects, and more generally, how cooparative TF binding may mechanistically underpin cellular differentiation.

4) Finally, the authors view the genome as one-dimensional while 3D confirmation is important for enhancer function as well. In that context, one TF

that is very obviously missing is CTCF, as it does affect chromatin accessibility. Investigating the relationship with CTCF would also alleviate the current issue that the authors treat the (3D) genome as a one-dimensional. Getting back to CTCF - do the low-affinity binding sites interact with CTCF? Is there reason to believe they would be involved in - or not involved in - shaping 3D chromatin architecture?

Minor points:

1) Figure 3E,F, what are the pattern in accessibility? Seems to small for nucleosomes

2) Effects for validations often don't fully recapitulate the predictions but go in the same directions :

e.g. p8: "These results differed slightly from the predicted effects (Figure S3F), but confirmed […]"

Or p9: "While the observed effects differed slightly from the predicted effects, […] (Figures 4B and S1K)."

do these discrepancies with the predictions point at specific / systematic weaknesses of the model that can be addressed in the futures?

3) Discussion: Another study to integrate into the interpretation is Baderna et al (https://www.biorxiv.org/content/10.1101/2025.03.26.645413v1), which uses not only DNA-sequence context but also chromatin context (modifications) to interpret cooperative and non-cooperative TFs.

Authors' response to the first round of review

Point-by-point response letter to the reviewers

Dear reviewers,

We genuinely appreciate the suggestions regarding the manuscript "Widespread low-affinity motifs enhance chromatin accessibility and regulatory potential in mESCs". We were pleased that our work was described as an "impressive" study addressing an "important outstanding question", that "asks all the right questions", and that it "should thus be of interest to a broad readership". The provided comments were comprehensive, thoughtful, and constructive, allowing us to significantly improve the manuscript.

The most notable additions include 1) additional model training with different region selections to confirm that our conclusions on motif cooperativity are robust, 2) the addition of *in silico* experiments showcasing how small pairwise motif cooperativities can result in large contextual effects in a genomic context, 3) an expansion of our kinetic model simulations showing how motif affinities of two transcription factors interplay cooperatively to change accessibility, 4) results showing how pioneering cooperativity impacts not only enhancer activation but target gene expression, and finally 5) leveraging our approach to show that cooperative motif arrangements are also important for developmental time courses in vivo, using neuronal differentiation as an example.

In this response letter, each question is addressed in blue text, with individual points separated into section identifiers. In the manuscript, notable additions and changes are shown in red text. Code and analysis pertaining to all changes have been updated in GitHub under the same repository.

Reviewer #1

Weilert et al used a deep learning-based approach in combination with genetic perturbation experiments in mESCs to systematically map and assess the contribution of low-affinity TF-binding sites to DNA accessibility. This is a significant question in the field, since previous studies showed the importance of low-affinity TF-binding sites at selected loci for enhancer specificity in vivo (e.g., Farley, Levine, Crocker, Stern labs) and in vitro (e.g., Bulyk lab). However, their general importance for enhancer activity and their role in opening chromatin remained unclear.

The authors trained a state-of-the-art BPNet model on ChIP-nexus data to predict TF binding for 5 pioneer factors (Oct4, Sox2, Nanog, Klf4, and Zic3) and ChromBPNet on ATAC-seq data to predict DNA accessibility. The authors then systematically mapped contributing TF motifs and showed that even low-affinity motifs, defined by low PWM scores, significantly contribute to the model. Importantly, they performed experimental validation of model predictions by mutating both high- and low-affinity pioneer TF-binding sites, confirming their contribution to endogenous DNA accessibility (Fig. 1G,H; 3E,F and 4A). They further demonstrated that, especially for low-affinity motifs, the context in which the motif appears is crucial for pioneering activity. While the median predicted effect on accessibility is low for low-affinity motifs when surrounded by random sequence, it increases significantly when these motifs are present alongside other motifs in a genomic context. Based on these results and supported by modeling, the authors conclude that low-affinity motifs cooperate with neighboring motifs to increase accessibility.

Overall, this is an important study that clearly shows the general importance of previously overlooked low-affinity TF-binding sites for DNA accessibility genome-wide for several factors.

We thank the reviewer for this positive assessment!

That said, there are concerns about study design and data interpretation that should be addressed before publication. Most notably, the authors introduce a systematic bias in a study design by using a training set with heavily overrepresented accessible chromatin regions and focusing on the SOX2-OCT4 motif, which is already cooperative, undermining claims about context and potentially missing important regulatory logic. Moreover, the definition of low affinity is ambiguous. Finally, the data presented cannot rule out a simple additive model in which each TF contributes additively toward general accessibility.

We appreciate this feedback and have now addressed these points below. Briefly, the training approach follows standard best practices, but we have now added additional training paradigms to the manuscript to reassure the reader that the interpretation is not dependent on the training set. Regarding the motif cooperativity, we agree that multi-way motif cooperativity is indeed what the model has learned, and we have now added an additional **Figure 2F,G** to more explicitly show that a large proportion of the "context scores" comes from the cooperativity of multiple motifs, while the pairwise cooperativity between motifs is indeed fairly small.

These and other comments are listed below:

1.0. [Region selection for model training] The training set, which includes ~150,000 1-kb open chromatin regions, is heavily biased toward accessible regions, which may bias model findings. This bias may cause the model to overtrain on contextual scores within open chromatin, thereby missing features of repression. They should clarify the rationale for limiting the analysis to 154,827 1-kb intervals. Do the authors observe the same findings if the model is trained on the entire genome? These comparisons should be included in the revised manuscript.

We appreciate this clarifying question. Our training approach is based on extensive experience with various genomics data sets and is consistent with approaches from other labs (e.g. Maslova et al PNAS 2020; Kim et al Nat Genetics 2021; Taskiran et al Nature 2023; Pampari et al biorXiv 2025; Chandra et al biorXiv 2025, Liu et al biorXiv 2025). For the model to learn how motifs interact, it has to be trained on regions that have a high content of relevant motifs, and the easiest way to do so is to "bias" the training set towards accessible regions. This does not prevent our model from learning repressive motifs (see Section 2.7 regarding the *Hic1* repressive motif), since repressive motifs are typically found in accessible regions intermingled with pioneer motifs. In addition, whenever training ChromBPNet models, we purposely include a fraction of regions of low accessibility (at least 5%) in order for the model to learn a reasonable spectrum of accessibilities. In this paper specifically, we used the regions bound by the five pluripotency transcription factors of interest so we could directly compare this model to the model of transcription factor binding. These regions naturally have a wide range of accessibility (described now in the **Methods**), thus passing our criteria for a good training set, and are intended to focus specifically on the accessibility behavior of these five pluripotency TFs.

To provide the reader reassurance that the training set selection did not affect our conclusions, we have now trained additional cross-validated accessibility models with different region sets, including on 2.55M genome-wide regions, 113K accessible-only regions, and 226K regions split between accessible and inaccessible ones. These sets produce small differences in model performance but **all models learned the key pioneer motifs, the importance of low-affinity motifs, as well as the distance-dependent rules of motif cooperativity between key pioneer motif pairs**, supporting the conclusions described in the manuscript (new **Supplementary Table 3 and 6, additions to Supplementary Tables 4-5**). We have expanded upon these models and their robustness in the **Methods** under the **Model Training and Validation** section and referenced these models, when relevant, in the manuscript directly.

1.1. [Definition of low-affinity motifs] It is unclear how "low-affinity" is defined. This is an important consideration, as studies on low-affinity motifs will be compared, and it would be helpful to know whether the reported low affinities are actually comparable. If it's based solely on the match score to the PWM, did the authors use consistent cutoffs across different TFs? Are these cutoffs similar to those reported for low-affinity TFBS in functional enhancers (Crocker et al, 2015; Farley et al, 2015; Lim et al, 2024, etc.)?

This is an important question–we have often debated about how to define "low-affinity" throughout this work. The problem is that there is no general sequence consensus cutoff for low-affinity motifs since the extent by which low-affinity motifs are bound *in vivo* is dependent on the cellular TF concentration and the K_d of the motif. Consistent with this, experimentally lowering the TF's concentration affects the extent by which low-affinity motifs were learned and mapped by the model (as shown in Figure 6B).

Given the absence of a consistent cutoff, most published work in the last decade has defined low-affinity motifs as "sub-optimal" or "less than perfect matches" to their cognate motif, typically quantified by PWM or in vitro binding representations (e.g. Farley et al 2015 Science; Lim et al 2024, Nature; Kim and Wysocka 2023, Mol Cell).

This "less than perfect" match criteria has also been applied to analyze *Oct4-Sox2* composite motifs in independent previous work (Waite et al 2024, PLoS ONE). We note that the low-affinity *Oct4-Sox2* motifs identified by Waite et al were also identified as low-affinity motifs in our study: they gave PWM match score quantiles ranging from 0.65 to 0.98., which mainly fall into our "middle 5K mappings" of *Oct4-Sox2* motifs in Figure 1D. Thus, our low-affinity motifs are consistent with previous literature and capture motifs of even lower affinity.

To be clear about this definition, we defined low-affinity motifs at the beginning of our results section as "imperfect motif match" and cite the review by Kim and Wysocka 2023, Mol Cell, which explicitly uses the same definition:

pg. 4 "For simplicity, we refer to an imperfect motif match as 'low-affinity' (Kim et al, 2023), regardless of its in vitro binding affinity".

Fig1G: Are neighboring Sox2 motifs high or low-affinity? Please state the cutoff thresholds if yes.

The *Sox2* motif at the *Btbd11* enhancer is an optimal match to the PWM representations of *Sox2* and is thus defined as high-affinity. We have clarified this in the results section:

pg. 5: "We selected the previously examined intronic Btbd11 enhancer since we discovered that, **next to a high-affinity *Sox2* motif, a low-affinity *Oct4-Sox2* pioneer motif is present** with key mismatches across both the Oct4 and Sox2 motif components (Figure 1G)."

We have also clarified this in the *Sox2* labels in F1G as well as the legends of F1, F4, and SF1.

1.2. [Additive cooperativity] The authors claim that pioneering cooperativity is the primary mechanism by which low-affinity TF motifs contribute to accessibility. However, a simpler additive contribution, in which each motif contributes a little bit, cannot be excluded. Moreover, their experimental data support an additive model rather than cooperativity. First, the effects of low-affinity motif deletions (Fig. 1H and 4B) seem to be rather mild, which is more consistent with an additive model. Second, the result of the combined deletions in 4B shows a marginally smaller effect than a simple sum of individual deletions, indicating that it's mostly additive, but the authors interpret it as "cooperativity". Third, in Fig. 3D, they delete only strong Sox2 motifs, not the weak ones, and the results further support an additive model (3G,H). The authors should either remove the cooperativity conclusion, for which they don't have experimental validation, or call it context-sensitivity or context-specificity, which is more accurate given the data presented.

The reviewer is correct that the pairwise cooperativity between two motifs is just a bit more than additive, which is predicted by the model, matches our CRISPR validations, is recapitulated in the kinetic model and is summarized in Figure 4. While the pairwise motif cooperativity is small, it does however add up when multiple motifs are present, creating a large difference between the effects of a motif in isolation versus its effect in context, our initial observation from the model interpretation in Figure 3.

It seems that the reviewer acknowledges the "context-specific" enhancements of motifs, but questions whether it can be explained by the pairwise motif cooperativity we describe. To bridge this gap, we have now done an intermediate *in silico* experiment, where we ask how much accessibility in a region is explained by the cooperativity of all motifs present (if there are at least two). Specifically, we isolated non-motif effects (such as CpG/GC content as shown by Fenouil et al 2012, Genome Res; Papin et al 2021, J Mol Biol), the effects of each motif added together, and the effects due to motif cooperativity. This shows that the cooperative effects grow steeply with the increasing number of motifs, as one would expect for a larger number of pairwise interactions between the motifs.

We now show this analysis in the revised manuscript as **Figure 2F,G**.

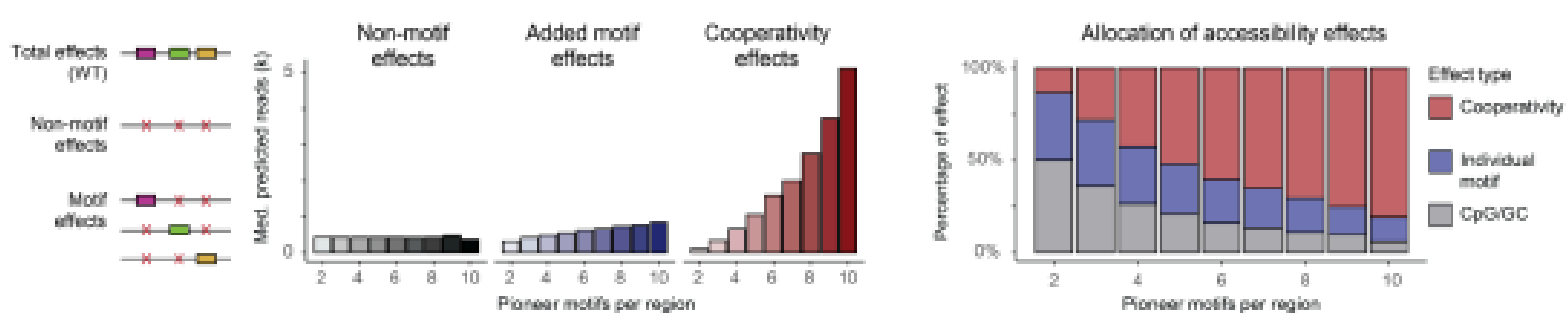

1.3. [The role and treatment of the *Oct4-Sox2* composite motif] The authors focus their analysis and experiments on the composite OCT4-SOX2 motif, treating it as a binding site for a single TF, which introduces some confusion and complicates interpretations.
Specifically:

o The OCT4-SOX2 motif does not appear representative of other pioneer TF binding motifs, which do not directly cooperate. Are the same findings true for more general pioneers?

The *Oct4-Sox2* motif is a composite motif that is inherently strong due to the DNA-mediated binding cooperativity between Oct4 and Sox2. When interpreting a deep learning sequence model, composite motifs are returned as a separate motif pattern, which is why we are treating the *Oct4-Sox2* motif as its

own motif. We chose it as a showcase because its inherent strength allows a wider range of mutations to the consensus motif, thereby providing a large range of effects.

The same trends are observed for other motifs, just at a smaller range, and we have now added this to the manuscript. In the new **Supplementary Figure 1G**, we show that Sox2, Klf4 and Zic3 high-, middle-, and low-affinity motifs mapped by ChromBPNet are bound by the corresponding ChIP-nexus experiment at higher levels than the highest-affinity PWM-only mapped motifs.

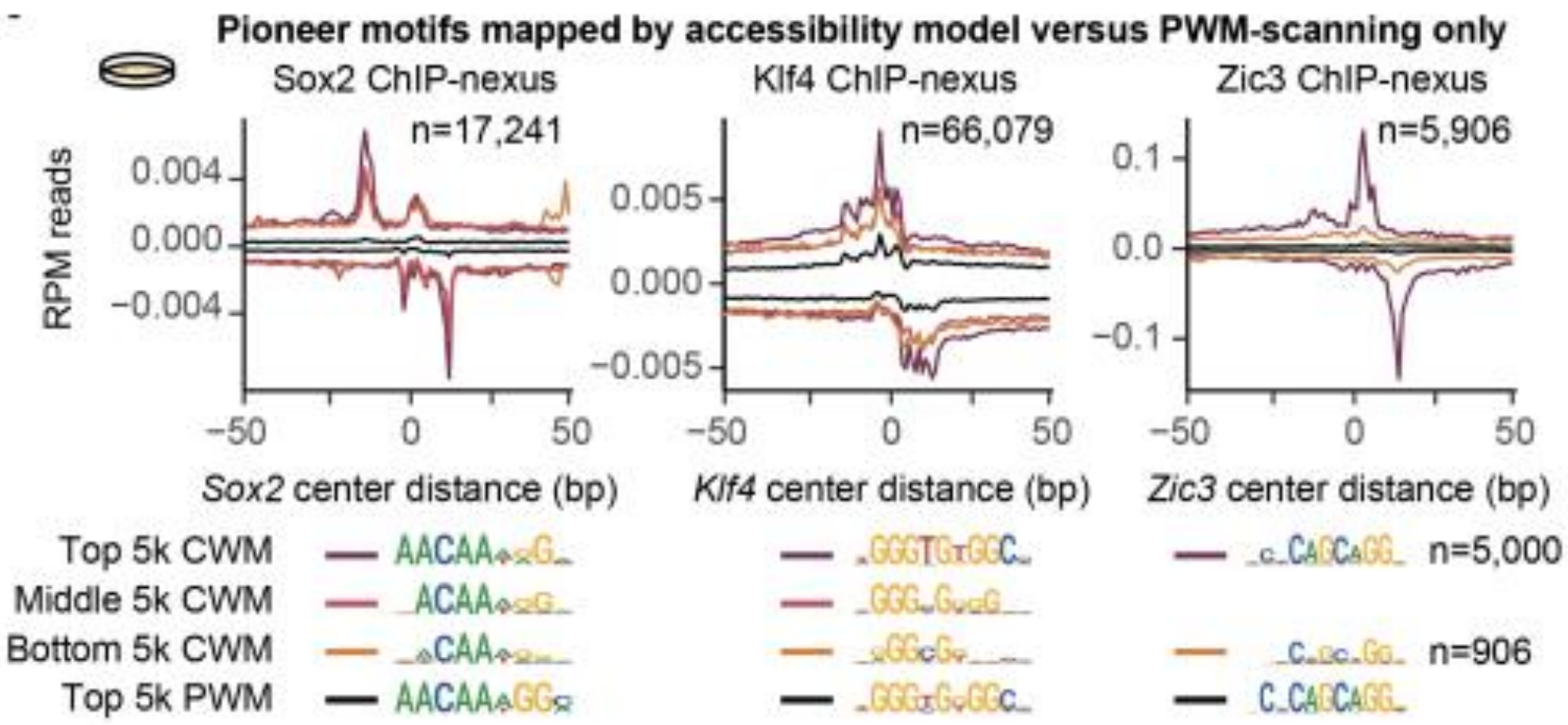

o How exactly is the OCT4-SOX2 affinity defined? It often appears that the OCT4 and SOX2 motif matches degrade independently (Fig 4D, Fig 6B, etc.). In Fig 1E, the bottom mapping, presumed to be low affinity, contains a perfect OCT4 motif and a degenerate SOX2 motif. It would be helpful to analyze these motifs separately.

The affinity of the *Oct4-Sox2* composite motif is defined through the PWM score of the entire composite motif. The observation that the *Sox2* motif component degrades more readily (Figures 1D, 4D, 6B) is indeed interesting since it suggests that a perfect *Oct4* motif can cooperate with a degenerate *Sox2* motif when correctly spaced. We know that this motif is not a single *Oct4* motif because we observe *Sox2* ChIP-nexus footprints on them (Figure 1E).

Whether this observation means that Oct4 binds first is however not clear. Previous data on imaging (Chen et al 2014, Cell), structural approaches (Michael et al 2020, Science) and *in vivo* pioneering function (Soufi et al 2015, Cell) are contradictory in this regard and did not provide a clear answer. Some work proposes that Sox2 initiates pioneering across the *Oct4-Sox2* heterodimer motif, after which Oct4 stabilizes Sox2 binding at the loosened chromatin, working to more effectively remodel chromatin (Chen et al 2014, Cell; Malik et al 2019, Nat Comm; Tan and Takada 2020, Proc Natl Acad Sci USA; MacCarthy et al 2022, Nuc Acids Research). Conversely, other work has found that Oct4 can access nucleosomal sites more robustly than Sox2, suggesting that Oct4 would bind first (Li et al 2019 Cell Rep).

Thus, we believe that our model interpretation is correct, but due to the contradictory previous evidence, we did not want to speculate about the mechanistic underpinning of this observation. Thanks for asking though.

o While SOX2 is examined independently, OCT4 is not (Fig1C, Fig4, and Fig6). The same analysis should be presented for OCT4 as well.

Every binding and accessibility model (including additional models trained in Section 1.0) learned not only the *Oct4-Sox2* and single *Sox2* motifs, but also the single *Oct4* (as summarized by TF-MoDISco). However, upon mapping the learned motif representations, single *Oct4* motifs were rare (**132** distinct mappings) compared to the single *Sox2* motifs (**59,781** distinct mappings). Moreover, over 50% *Oct4* motifs were mapped across ERVs, which hinders the study of syntax rules. Due to these low numbers, we chose to exclude Oct4 as a showcase motif by which we characterize pioneering syntax in mESCs.

To provide these results to the reader, we have now added the *Oct4* motif and the learned ERV-enriched mappings via side-by-side PWM representations in Supplementary Figure 1B for the binding models, and we have expanded this representation for **Supplementary Figure 1C** for the accessibility models:

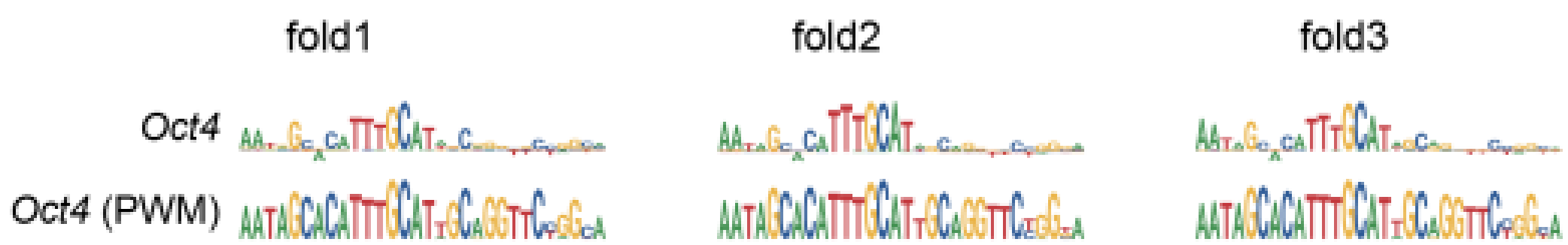

o OCT-SOX composite motif is an inherently cooperative interaction, which makes comparisons to other motifs confusing - is it a three-way cooperativity?

It could indeed be seen as a three-way cooperativity. As we have shown above, accessible regions tend to have multiple motifs that each interact with each other to produce the predicted outcome as multi-way cooperativity. However, since the *Oct4-Sox2* motif is so frequent, always has the same spacing, and is frequently found together with other motifs, we can treat it here as its own single motif and obtain consistent results.

1.4. [Clarification of regions mapped when comparing PWM- vs CWM-scanning] Fig 1F: This result is not surprising, given that only a single-digit percentage of all motif occurrences in the genome are bound by a given pioneer TF. A more rigorous control would be to score PWMs within already accessible peaks (since ATAC-seq data exist). Is ChromBPnet better at mapping TFBS than simply overlapping PWMs with ATAC-seq/ChIP peaks?

When comparing PWM-scanning with CWM-scanning, the regions allowed for mapping were limited to the original ~150k genomic loci bound by Oct4, Sox2, Klf4, Nanog, and Zic3. It would not be a fair comparison to compare all motif occurrences in the genome for the exact reason this reviewer noted. We recognize this was poorly worded and have clarified this in the **Results** section (with corresponding clarification in the **Methods** section):

pg. 5: "**As a comparison, we mapped motifs in the same genomic regions using traditional PWM scanning (Methods).**"

1.5. [Addressing biases in motif groups] Fig 6: Potential biases in comparing group versus single motif arrangements. How is affinity managed across groups, such as a high-affinity motif group versus low-affinity ones, or vice versa? The authors should account for the potential confounding effects of motif-rich versus motif-poor regions. Perhaps compare grouped versus single motif arrangements of Oct4-Sox2 motif combined with non-ESC or non-Pioneering TF motifs.

In order to retain sufficient numbers of *Oct4-Sox2* motifs, we defined affinity groups as the top and bottom tertile of sequence affinities for all *Oct4-Sox2* motifs. This allowed us to choose motif affinities that were sufficiently similar between grouped/isolated arrangements, depicted in Supplementary Figure 6G.

There was however an interesting exception. The low-affinity motifs in the grouped arrangements had significantly poorer sequence matches compared to the low-affinity motifs in isolated arrangements. This makes sense since low-affinity motifs can only be functional when nearby other motifs. This small difference would have predicted stronger effects for the isolated low-affinity motifs in the time-course accessibility changes, but we observed the exact opposite. The grouped arrangements had a bigger effect despite these motifs being of lower affinity.

We agree with the reviewer that investigating additional motifs across these *Oct4-Sox2* regions is a good control. Thus, we have a subplot to **Supplementary Figure 6** which reexamines grouped/isolated and high/low-affinity *Oct4-Sox2* motif arrangements with all pioneer motifs (Supplementary Figure 1C). In this way, we show that despite the changes in fundamental motif composition the grouped motif arrangements impart stronger accessibility effects than isolated arrangements, at similar levels as in Figure 6E.

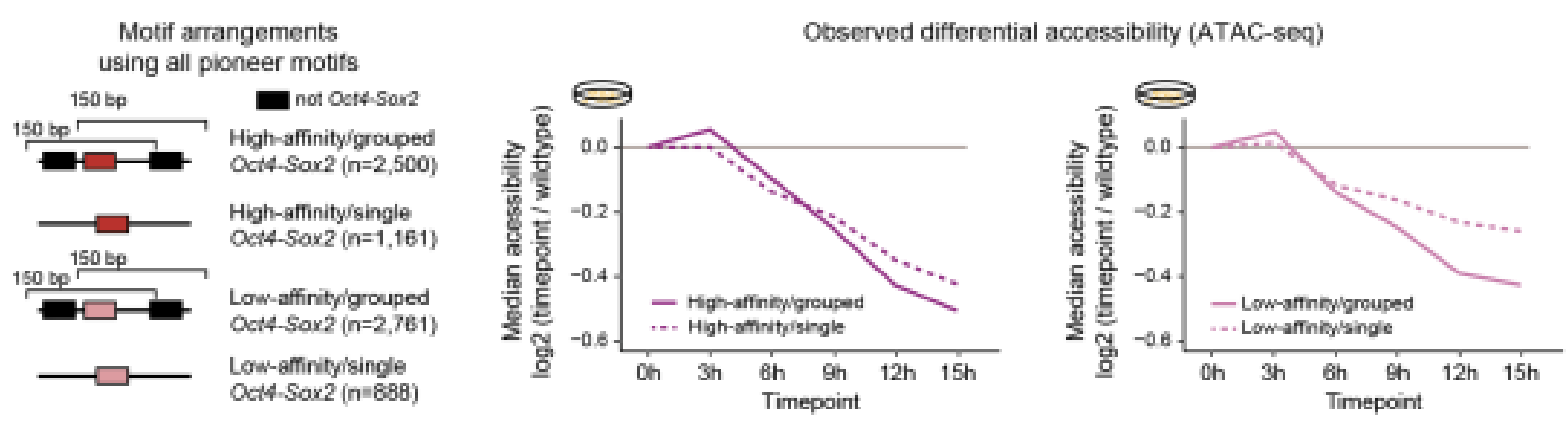

1.6. [Addressing biases in motif groups] Fig 6F/G: Are low-affinity/single motifs accessible at all (only fold-changes shown)?

Yes. We had measured differences in initial accessibility levels for each arrangement (**Supplementary Figure 6H**). In this way, we could confirm that our low-affinity/single motif groups were indeed accessible. To help the reader understand that all four motif arrangements are sufficiently open in the initial time point, we have added annotation lines depicting median accessibility across all motif-containing regions mapped by our models (red dashed line), shown below.

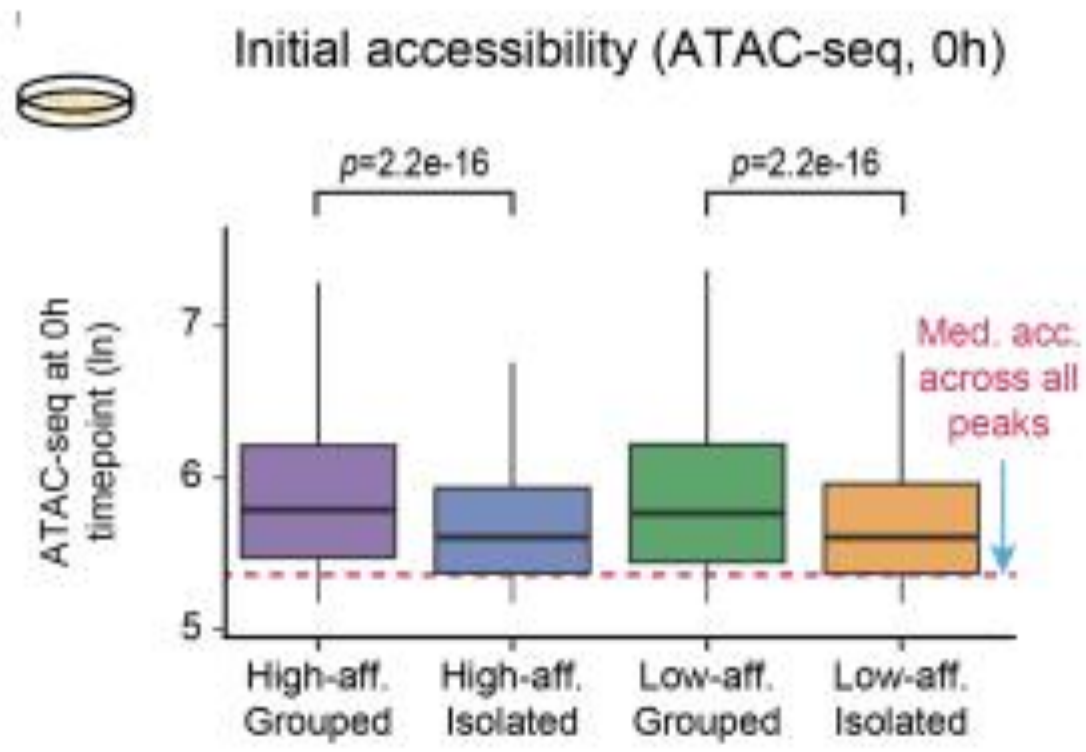

1.7. [Quantification of Btbd11 *Oct4-Sox2* motif mutation] Fig 1H: Provide a quantitative comparison (normalized for read density) as it is done in Fig 3G, H

As requested, the quantifications of read density for the *Oct4-Sox2* motif mutations at the Btbd11 enhancer (depicted also in F4B) have now been added as **Supplemental Figure 1M**, shown below.

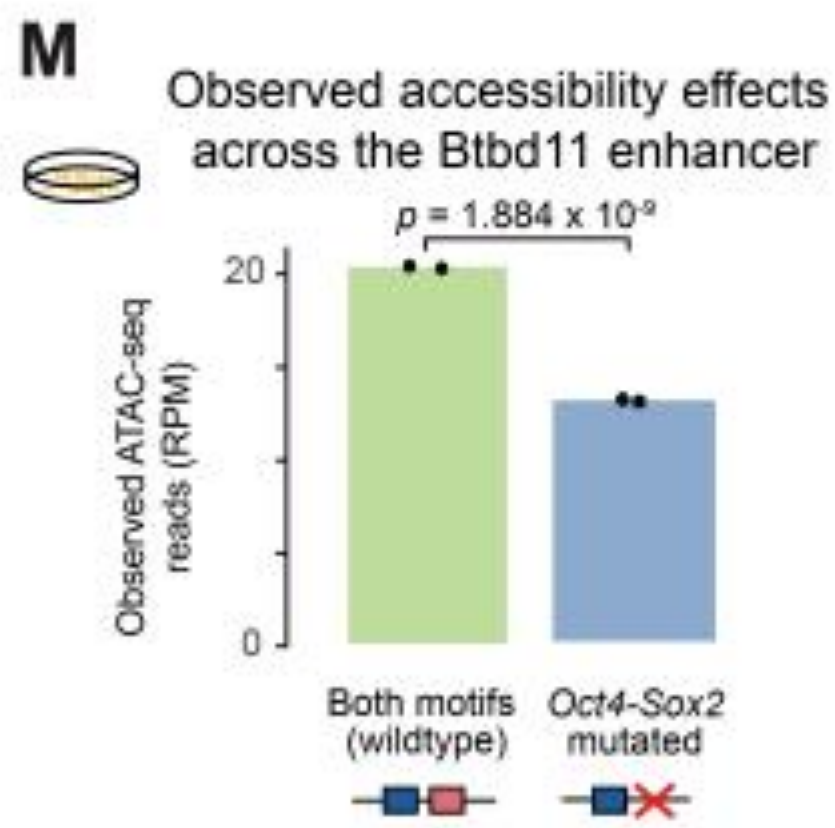

1.8. [Consistency in referencing models] After Fig 1, it is not clear which model is used throughout the text. Is it BPnet, chromBPnet, both? Please clarify.

We appreciate the reviewer pointing this out, as Figures 2 and 3 rely heavily on the comparison of accessibility (ChromBPNet) and binding (BPNet) predictions. We have clarified that both model predictions are used in the appropriate sections below and made minor adjustments beyond these excerpts to ensure it is clear which models are used throughout the manuscript.

pg. 6: "To do so, we extracted two measurements from **both our accessibility (ChromBPNet) and binding (BPNet)** models across all mapped motifs, isolation scores and context scores, which we will use throughout this work."

pg. 8: "To visualize this correlation, we plotted the context scores **derived from both the accessibility (ChromBPNet) and binding (BPNet) models** for all motifs of the same sequence…"

1.9. [Elaborate on isolation/context score] Provide a detailed explanation of isolation and context score derivation in the main text (not just figure legends) since these are novel key definitions. Also how are isolation/context scores calculated when the same motifs occur multiple times within a short stretch? Are these discarded?

As per the reviewer's suggestion, the following expansion has been added in the main text:

pg. 6: "Isolation scores (also called marginalization scores[21,68] or global importance scores[69]) measure the predicted effect of each motif sequence *in isolation*, **by injecting a motif sequence into randomized sequences and measuring the average log-fold-change over the uninjected control (Figure 2A, Methods).** For a TF binding model, the isolation scores for mapped motifs have been shown to be a proxy for relative motif affinities.[68] In contrast, context scores, a motif-centric expansion of *in silico* mutagenesis[70,71] or the necessity test,[72] measure the predicted **log-fold-change** loss when a motif is **mutated** in its genomic sequence context[36] (Figure 2B, Methods)."

We have expanded the "Isolation and context scores" Methods section to address how we deal with closely placed motifs and their context scores.

pg. 27-28: "Background sequences were derived by sampling trained peak regions to match the baseline CpG enrichment and GC-content of the mouse genome, then di-nucleotide shuffling the underlying sequences. CpG ratios were calculated as (GC-content / 2)^2 and normalized to each region width, as previously described.[120] **Since the background sequences may contain functional motifs during *in silico* injections, we average the effect of 256 trials, which averages out these spurious effects.**

In contrast to isolation scores, the "context" scores of a mapped motif involves measuring a motif's effect within its genomic context rather than in isolation, as described previously.[21] Mapped motif sequences were mutated via replacement with random insertions. Across 16 trials of mutations, average mutation response was calculated, then log-fold-change was computed between mutated and wildtype profiles. **If two or more motifs are in the same region, but do not share overlapping coordinates and are at least 20 bp apart center-to-center, individual context scores are calculated for each motif.**"

1.10. [ChIP-nexus/ATAC peak overlaps] Provide more detail on the overlap between ChIP-nexus and ATAC-seq peaks (unique vs. shared Venn diagram).

We have expanded the Methods section under "Model training and validation" to add more detail about the overlap between ChIP-nexus and ATAC-seq peaks.

pg. 23-24: "Input DNA sequences were derived from the sequences of 154,827 IDR-reproducible peak regions, described above. **Of these peak regions, 71% of them overlapped with IDR-reproducible ATAC-seq peaks (n=113,160). We validated that these genomic regions possessed sufficiently diverse accessibility for ChromBPNet model to distinguish between accessible and inaccessible loci by comparing their interquartile ranges (IQR). The IQR of IDR-reproducible ATAC-seq peaks was 1,142 reads with quartile ranges of [513,1655] reads. The IQR of IDR-reproducible TF peaks was 1,749 reads with quartile ranges of [384,1,749] reads. Because the binding peaks covered a broader range of accessibility levels, we deemed it as an appropriate dataset by which to train both binding (BPNet) and accessibility models (ChromBPNet).**"

1.11. [Strongest pioneer motif clarification] Fig 3: Define "strongest pioneer motif". Is this based on motif score or accessibility contribution?

As per the reviewer's suggestion, the following expansion has been added in the main text:

pg. 8: "We found that high pioneering effects of a weaker pioneering motif were best predicted by small distances to the strongest pioneer motif, **thus the motif with the highest accessibility context score in the region** (Spearman correlation 0.37 to 0.38)**.** "

1.12. [Typo] Fig 6E has an incorrect label (bottom should be "low-affinity/single")

This typo has been corrected, thank you for catching that.

1.13. [Clarification of labeling] Fig 4E: (0-70] then "(70,180]" is confusing. Please make labels consistent.

This typo has been corrected and assigned more explicit labeling, thank you for noting this.

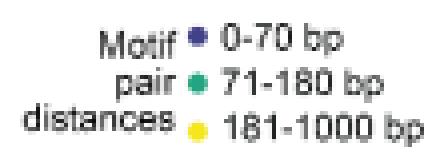

Reviewer #2

The study 'Widespread low-affinity motifs enhance chromatin accessibility and regulatory potential in mESCs' from Weilert et al trains deep learning models on DNA accessibility and ChIP-nexus TF occupancy data to investigate how the combinatorial presence of TF motifs (enhancer context) shapes enhancer accessibility and its sensitivity to TF concentration in mESCs. They show that using the model's contribution scores improves motif mapping by enhancing the specificity with which lower affinity motifs are mapped (based on ChIP-nexus data) compared to using PWM scoring alone. Based on this mapping, they investigate how combinations of pioneer motifs modulate enhancer accessibility in silico, finding widespread cooperativity, by which pioneer motif combinations within nucleosome-range distances lead to more accessibility as the combination of their individual contributions would suggest. The authors provide a kinetic non-equilibrium model for this behavior, which shows that motif cooperativity leads to an increase in the fraction of accessible enhancers and a more rapid rise in opening as a function of TF concentration. Using time-course data measuring DNA accessibility upon degradation of Oct4, they show that Oct4-Sox2 dimer motifs that occur in cooperative contexts (multiple pioneer motifs) lead to a steeper and higher magnitude drop in enhancer accessibility and RNA-expression compared Oct4-Sox2 motifs in isolation and that this effect occurs for both high and low affinity sites. This suggests that motif combinations heighten an enhancer's sensitivity to changing TF concentrations, a finding in line with motif cooperativity.

The study addresses an important outstanding question in the field of regulatory genomics, namely how a combinatorial TF code contributes to gene expression control. It outlines how pioneer nucleosome-range cooperativity may contribute to fine-tuning enhancer responsiveness to changing TF concentrations and how low affinity sites may contribute to this phenomenon. This finding provides an interesting angle on non-canonical (not directly mediated through DNA binding) TF cooperativity and how it modulates enhancer activities and ultimately gene expression control and should thus be of interest to a broad readership. The strongest point of the study is the use of time-course experiments that show that enhancers with multiple pioneer motifs lead to a more rapid and higher magnitude change in overall accessibility as a function of decreasing TF concentration for a single TF (Oct4), which supports the cooperativity model.

We thank the reviewer for this positive assessment!

A weak point of the study is that the modeling on cooperativity is solely based on in-silico predictions and is thus entirely depended on the model's ability to learn cooperativity in the first place, which may or may not be the case. While there are some CRISPR experiments, the generated cell lines do not explicitly test for the presence of motif cooperativity, i.e. mutating one or the other or both pioneering motifs within proximity and comparing accessibility levels to confirm cooperativity. Another emphasized aspect is the importance of low affinity binding sites, however, after reading the study one is left with the impression that different affinity sites are essentially interchangeable, and low affinity sites are simply used because they are easier to generate. This contradicts previous work showing that turning low affinity sites into higher affinity ones can have detrimental consequences.

In summary, the study would benefit from providing hard experimental evidence for TF motif cooperativity and a clear delineation of how low-affinity sites contribute to an optimal enhancer response curve (i.e. exploring how combinations of different affinity motifs alter enhancer accessibility in the kinetic model).

We appreciate this feedback and have tried to carefully address each point. We felt that some of them are based on misunderstandings, and thus we hope that our revised manuscript is more clear about the data and what the message is. As explained below, we certainly did not want to convey that different motif affinities are interchangeable, and have revised the text to be more deliberate in this regard. Furthermore, we are showing one example of CRISPR validation of motif cooperativity, and while this is limited experimental evidence, the results agree with the model predictions and apply to more complex experimental data, which we have now strengthened with additional analyses: **Figure 2F,G** shows how such small cooperative effects grow across multiple motifs, and **Figure GH-J** shows that grouped motif arrangements also predict steeper accessibility changes during a time course of neuronal differentiation in agreement with the observed experimental data. Moreover, we have now included additional simulations to show how the response curves change in the two-TF kinetic model, which also shows the importance of motif affinity. Overall, we have rewritten the corresponding sections extensively and even changed the title of the manuscript to improve the clarity.

2.1. [Region selection for model training] In the text it says that BPNet models were trained independently of one another, however, from the methods it reads as if the regions for training the accessibility model were pre-selected based on information of the ChIP-nexus data, thus informing the accessibility model of true TF occupancy. How would the results change if this was not the case, could this influence what motif combinations can effectively be learned in the accessibility model?

This is an excellent question. Targeted region selection is indeed often used to "encourage" sequence-to-function models to learn specific motif combinations, which can improve learning of syntax and rarer motifs. Conversely, training on overly general regions (e.g. genome-wide) can cause the models to ignore or under-weight the importance of rare or more subtle motif syntax. In our case, we trained the binding BPNet model and the accessibility ChromBPNet model on the same regions so that the two independent models had the same opportunity to learn each motif. In this manner, motifs that are differentially learned by the models likely have meaning. For example, the Nanog motif was only learned by the binding model, but not by the accessibility model, consistent with Nanog not being a pioneer TF (Maresca et al 2023 EMBO J).

To give the reader a better idea of what motifs are learned under different training paradigms, we have now trained similarly performing cross-validated accessibility models under different region sets such as genome-wide (2.55M regions), accessible only (113K regions), and a split between accessible and inaccessible (226K regions) (**Supplementary Table 2**). We found that **all models learned the key pioneer motifs** contributing to accessibility, which is summarized in a new **Supplementary Table 3**. Although our original models learned the greatest diversity of motifs, all models learned **the importance of low-affinity motifs, the distance-dependent contextual role of pioneers, and similar rules of motif cooperativity between key pioneer motif pairs**. Thus, our conclusions hold independently of the selected region set.

These results are now added as additional columns in what were originally Supplementary Tables 3 and 4 (now **Supplementary Tables 4 and 5**) and in a new **Supplementary Table 6**.

For an expansion on how a set of regions determine which motifs are found, please refer to Section 2.7 below.

2.2. [Experimental cooperativity] Experiments in Figure 3 demonstrate that some motifs (perhaps because they are near other pioneer motifs) lead to larger accessibility changes, but it does not explicitly test whether TF-A and TF-B act cooperatively. There are other explanations, like positioning of the motif with respect to the preferred nucleosome location. What happens if the low-affinity motif is deleted independently? How does accessibility change when the proximal or the low-affinity Sox2 motif are mutated in isolation, and is the sum of those less than the total accessibility measured in the native enhancer? Demonstrating at least for one case that the in silico predictions of cooperativity (green bars in subsequent plots) are real, would make this study much stronger as in its current form it hinges on the model's ability to correctly learn cooperativity, which was never experimentally validated.

The reviewer is correct–we do not directly test cooperativity in Figure 3 just yet, instead showing that an identical motif sequence within an enhancer can confer very different effects on accessibility. We wanted to first show that a motif's position within an enhancer is important, before then dissecting the reason for this position dependency. While we agree that additional mutations in the *Akr1cl* enhancer would be interesting, obtaining additional clones from CRISPR mutant experiments takes several months and thus is out of scope for this study. **Instead, we show motif cooperativity experimentally within the *Btbd11* enhancer in Figure 4B.** Here, guided by ChromBPNet accessibility model predictions, we experimentally mutated (1) the low-affinity *Oct4-Sox2* motif, (2) the high-affinity *Sox2* motif, and (3) both motifs simultaneously, finding that there was a cooperative component which contributed to the wildtype accessibility levels.

We recognize that this was not as clearly expressed as it could be. As the reviewer mentions at the bottom (Section 2.9), we agree there is a need for more delineation between different kinds of analyses. We have now added to each figure icons representing whether the data represented is a deep learning prediction, a kinetics simulation, or experimental evidence. Depicted below:

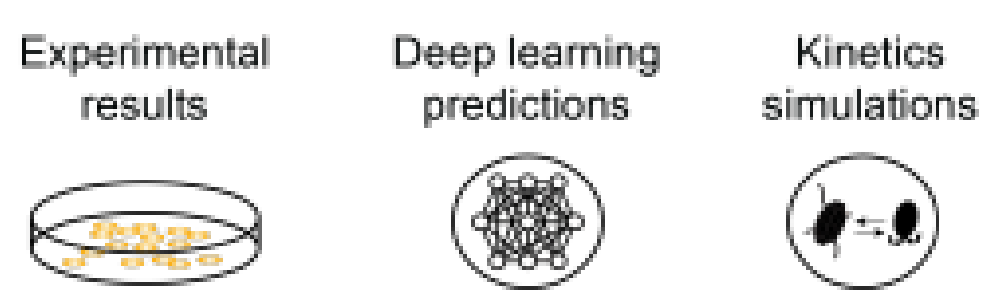

2.3. [Kinetic model parameter sweep] For the kinetic model it would help to illustrate the boundaries, e.g. what parameters are required to achieve 100% accessibility and how does this depend on the affinity of both TFs (varying both TF-A and TF-B). Can the authors provide an illustration of how different affinities alter the kinetic parameters and which parameter ranges (and motif affinities) are within physiologically relevant ranges? This gets to the point of how low and high affinity sites differ. They clearly are not interchangeable, as low affinity motifs operate at different TF concentrations and also lead to less cooperativity (SF5g).

We appreciate the opportunity to clarify the conclusions drawn from the kinetic model and perform additional simulations with the 2-TF model.

To be clear, the selected parameter ranges are conservative estimates of what would be physiologically plausible, described and justified in the **Methods** section based on available literature data. Within this regime, we have shown the effect of various parameters across plausible ranges in **Supplementary**

Figure 5C. It therefore appears to us that the expectation of 100% accessibility is based on a misunderstanding.

It is true that 100% accessibility in our simulations is usually not achieved under these parameter regimes. This is intentional, as the accessibility is measured across a cell population, and an enhancer is typically only simultaneously open in a fraction of cells. For example, our analysis of single molecule footprinting (SMF) data revealed that enhancers in mESCs (n=~48K) are on average ~34% accessible (Sonmezer et al 2021, Mol Cell). While this was known, our modeling uncovered that a given motif in a region can only achieve so much accessibility at saturated concentrations of this TF. This limit is what we define as "regulatory potential". What contributes to the "regulatory potential" is the affinity (1/g) of the motif, its cooperativity when paired (driven by opening effect (o) and closing effect (c)), and the intrinsic nucleosome affinity, proxied in our model by the ratio k_{close} / k_{open}. This ratio was selected to specify a higher affinity for nucleosomes, in line with data showing that pioneering motifs are arranged across nucleosomes with strong intrinsic affinities (Sun et al 2015, Genome Res).

To better define "regulatory potential" and how it limits the maximum accessibility state, we introduce the concept now earlier in the kinetic modeling section. Furthermore, we now explicitly compare the effect of motif affinity and motif cooperativity to avoid the impression that motif affinity is not important:

pg. 12: "Thus, putting a motif into a cooperative context confers distinct properties on chromatin opening than simply increasing the motif's affinity. While both motif affinity and motif cooperativity increase the chromatin accessibility at lower TF concentrations, only motif cooperativity boosts the accessibility beyond of what a single TF can achieve at saturating TF concentrations, when it fully occupies nucleosomal and naked DNA (Figures 5E, S5F and S5G). This suggests that pioneering cooperativity extends the regulatory potential of the motif."

Finally, we also appreciated the opportunity to show additional simulations with the 2-TF model, including changes in motif affinity. The reviewer made a good point that we did not explicitly show how the interplay of differing affinities or differing concentrations in a motif pair affects accessibility. This gets quite complex, which is why we initially omitted it, but we have now added two scenarios: (1) how the affinities of motif A and motif B contribute to the curve when the concentration of TF A increases and (2) how the same motif combinations respond when the concentration of both TF A and TF B increases.

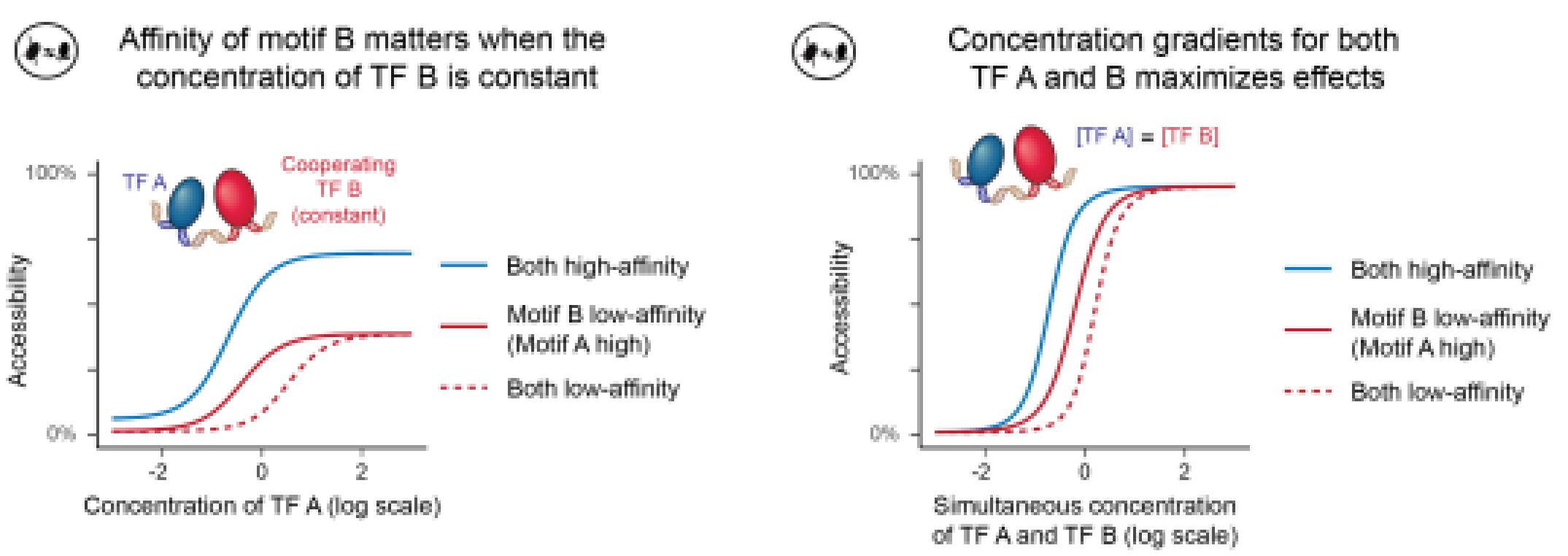

We find that the regulatory potential depends on the cooperativity between motif A and motif B. When motif B is of lower affinity (while keeping the concentration of TF B constant), then motif A receives less

cooperative input and achieves a lower accessibility maximum. This still depends on the affinity of motif A since a higher affinity motif will have larger effects earlier, when the concentration of TF A is still low.

In the second scenario, where TF B's concentration increases alongside TF A, the maximum accessibility is much higher (but still not quite 100%) since the effect of motif B can now also reach its maximum. In this case, the maximum potential is the same regardless of the motif affinities, but the motif affinities still matter when the TF concentrations are below saturation. The higher the motif affinities, the higher the accessibility.

These additions are intended to provide the reader a more comprehensive idea of how cooperativity depends on the complex interplay of many mechanistic properties (including TF affinities) at an enhancer. They have been added as **Supplementary Figure 5H** (with corresponding parameters reported in **Supplementary Table 9**). We have more explicitly referred to where the kinetic modeling can be found for interested readers in the **Data and Code Availability** statement.

Reading that high and low sites are essentially interchangeable and that low affinity sites are not there due to their affinity but rather of how easy they are to make is quite confusing. If it's so simple, wouldn't that put developmental enhancers at constant risk of overshooting their activity levels? Besides, other studies have shown that high affinity sites can overshoot the target range, so affinity clearly is relevant.

Again, we do not believe that high and low affinity sites are interchangeable. High-affinity motifs are able to achieve their maximum regulatory potential at far lower TF concentrations than low-affinity motifs. It is only at saturating TF concentrations that they could reach the same effect. TF concentrations are important during developmental transitions, and thus motif affinities obviously matter. What we learned from our kinetic modeling is that motif affinity alone does not determine the response to a specific TF concentration. **Rather, it is the motif affinity plus the added effect from cooperative interactions that determine its effect.** This cooperative effect is even more impactful when the TF concentration is saturated. While the response of motif A is at its maximum, the cooperative interaction with motif B can still increase the accessibility. We have now improved the text in both the **Results** and **Discussion** section to emphasize these points.

2.4. [Motif group controls] The data in Figure 6 is strong, however there is again no direct comparison of low and high affinity sites. It looks like high affinity has an impact on the magnitude of the change? Also, how comparable are the enhancers in terms of their affinity, i.e. are the grouped and single motif enhancers matched in their affinities (no statistically significant difference) ?

Indeed, Figure 6 shows that the magnitude of the change increases if an *Oct4-Sox2* motif is (1) high-affinity and/or (2) arranged in a grouped conformation with nearby *Sox2* and *Klf4* motifs. To arrive at this conclusion, we matched our region sets very carefully. We made sure that the motif affinities were sufficiently similar between grouped/isolated arrangements (**Supplementary Figure 6G**). We next examined the starting accessibility for all motif arrangements. We chose motif arrangements that were sufficiently open (**Supplementary Figure 6H**), though grouped motif arrangements had intrinsically higher initial accessibility levels than single motif arrangements, which is expected and unavoidable. By choosing relatively accessible regions for the low-affinity motifs in isolated arrangements, another unavoidable effect was that this group showed significantly higher sequence matches than the low-affinity motifs in the grouped arrangements, where low-affinity motifs can exist nearby other motifs. This small difference would have predicted stronger effects for the isolated low-affinity motifs in the time-course

accessibility changes, but we observed the exact opposite. The grouped arrangements had a bigger effect despite these motifs being of lower affinity. We have now expanded the **figure legend of Figures 6 and S6** and the main text to better lay out our rationale.

2.5. [Cooperativity and role of non-pioneer motifs] Could the authors comment on the fact that they do not observe cooperativity with non-pioneer motifs. Is this because of the constraints placed on the training regions (selecting against certain motif pairs), or could this have to do with what the model is capable of learning? Can the authors elaborate in their modelling how non-pioneers would behave? I assume they should contribute to preventing nucleosomes from returning? Why would this not lead to cooperativity as has previously postulated for nucleosome-mediated cooperativity?

We appreciate this question! As the reviewer mentions below in Section 2.6 by referencing Kribelbauer et al alongside our own observations (Brennan and Weilert et al 2023, Dev Cell), activator non-pioneer TFs have been linked to increased chromatin accessibility at enhancers *in vivo*. We do believe that non-pioneering TFs influence accessibility when they lead to strong enhancer activation, which our accessibility model is not designed to directly learn. Because enhancer activation occurs via indirect, higher-order recruitment of co-activators by multiple motifs, we suspect that this additional effect after pioneering is difficult to learn *de novo* from DNA sequence alone. Consistent with this, we previously found that non-pioneer motifs can sometimes be learned with ChromBPNet (Brennan and Weilert et al 2023, Dev Cell), but the learned motifs were weak and their roles appear to be highly contextual and concentration-dependent.

In our current work, our accessibility model did not return non-pioneering motifs, which is one reason Zic3 and Nanog binding were included in the binding model, so that we could compare cooperativity dynamics between motifs that wouldn't necessarily impact accessibility. We found no evidence that targeted region selection played a critical role in whether these motifs are interpreted to be pioneers since we found similar results when we trained additional models on different regions (Supplementary Tables 3-6, see Sections 1.0 and 2.1). It is however possible that the models learned very weak effects of non-pioneers. When we examined the effects of pioneer/non-pioneer motif pairs on accessibility, we saw scattered responses ranging from cooperative to non-cooperative (see the *Sox2*/*Nanog* pair and the non-responsive *Nanog*/*Nanog* pairs as reference). This result is however too speculative to include in the paper.

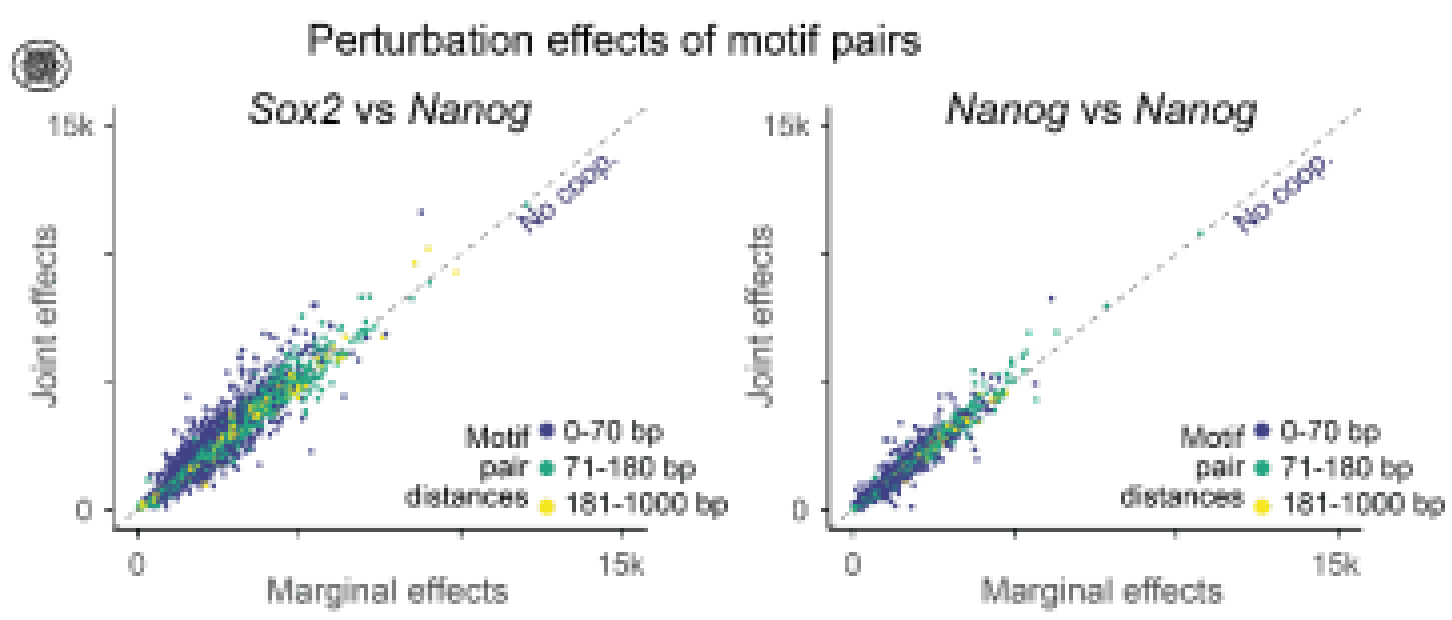

We did however consider the role of non-pioneers in enhancing chromatin accessibility in our kinetic model. Specifically, non-pioneers could bind to already accessible chromatin and prevent nucleosomes from returning (see **Section 1.2** and the new additions to **Figure 2**), a possibility which we have added in the **Discussion**.

2.6. [Kribelbauer et al] There was another recent study that looked at accessibility changes at SNPs that if I recall correctly found non-pioneer motifs enriched in the context when outsized accessibility changes occurred (PMID: 39363017). This seems somewhat contradicting, but might have to do with the differences in study design (SNPs as perturbations versus AI model), however it would be good to discuss how the study setup might influence the results.

This paper was of great interest to us when published. Indeed, the context-only motifs described by Kribelbauer et al could correspond to those of non-pioneer TFs, which we have also found to be highly contextual (Brennan and Weilert et al 2023, Dev Cell). This is an interesting question for the future and would require a side-by-side comparison of the two methods. Notably, the method by Kribelbauer et al used motifs from an existing HOCOMOCO database, while our deep learning models are not provided with motif information and are instead trained to learn *de novo* how combinations of different sequences predict accessibility levels. It is possible that this context is difficult to learn *de novo* but can be picked up by examining caQTL data.

As mentioned above, we now mention a role for non-pioneer TFs in the accessibility in the **Discussion**:

pg. 17: "For example, our kinetically modeled TFs were set to possess slightly more affinity for DNA in the open versus closed state. This parameter is the main determinant of pioneering strength in classically-employed thermodynamic equilibrium models of nucleosome-mediated cooperativity (Figure S5A). This contradicts the concept of a pioneer TF that exclusively opens chromatin through its ability to bind nucleosomal DNA**, but is consistent with the tendency of TFs to bind naked DNA more readily than nucleosomal DNA. By binding in the open state, a TF could reduce the rate of nucleosome reformation or promote the removal of neighboring nucleosomes. Beyond the influence of chromatin modifications on accessibility, this could partially explain the additional accessibility that is observed when enhancers are active or how non-pioneer TFs might contribute to chromatin accessibility (Kribelbauer-Swietek et al 2024). How exactly various nucleosome-mediated mechanisms directly or indirectly contribute to making chromatin accessible is an interesting topic for future mechanistic studies.**"

2.7. [Motif composition differences] This goes back to the first point. Was the accessibility model trained only on regions with ChIP-nexus confirmed binding of a select number of TFs? Do those regions differ in their motif composition from the accessible peaks not chosen? How would this affect the results? Does this lead to (as the author's state in their limitation) 'out-of-distribution' sampling?

The accessibility model was indeed trained on all regions with reproducible ChIP-nexus binding of our five TFs of interest. Again, we did this because we wanted the two model types (TF binding and chromatin accessibility) to have the opportunity to learn the same motifs, and because these regions happen to have a broad distribution of accessibility levels (interquartile ranges now reported in **Methods**). In our revised version, we now show that our conclusions hold even when we use different training paradigms (Section 2.1 and new **Supplementary Table 3**). We note that training models on different region sets returned in all three cases the key pioneer motifs of interest, but also that weaker, infrequent motifs such as *Yy1* and *Hic1* do not always pass the clustering thresholds of TF-MoDISco. Nevertheless, when queried, we found that all our accessibility models learned similar impacts of these weak motifs (figure below, showcasing key motifs for reference). This shows that ChromBPNet models learn robust motif grammar despite differences in region selection, and that our original training set is in this regard not "out-of-distribution" .

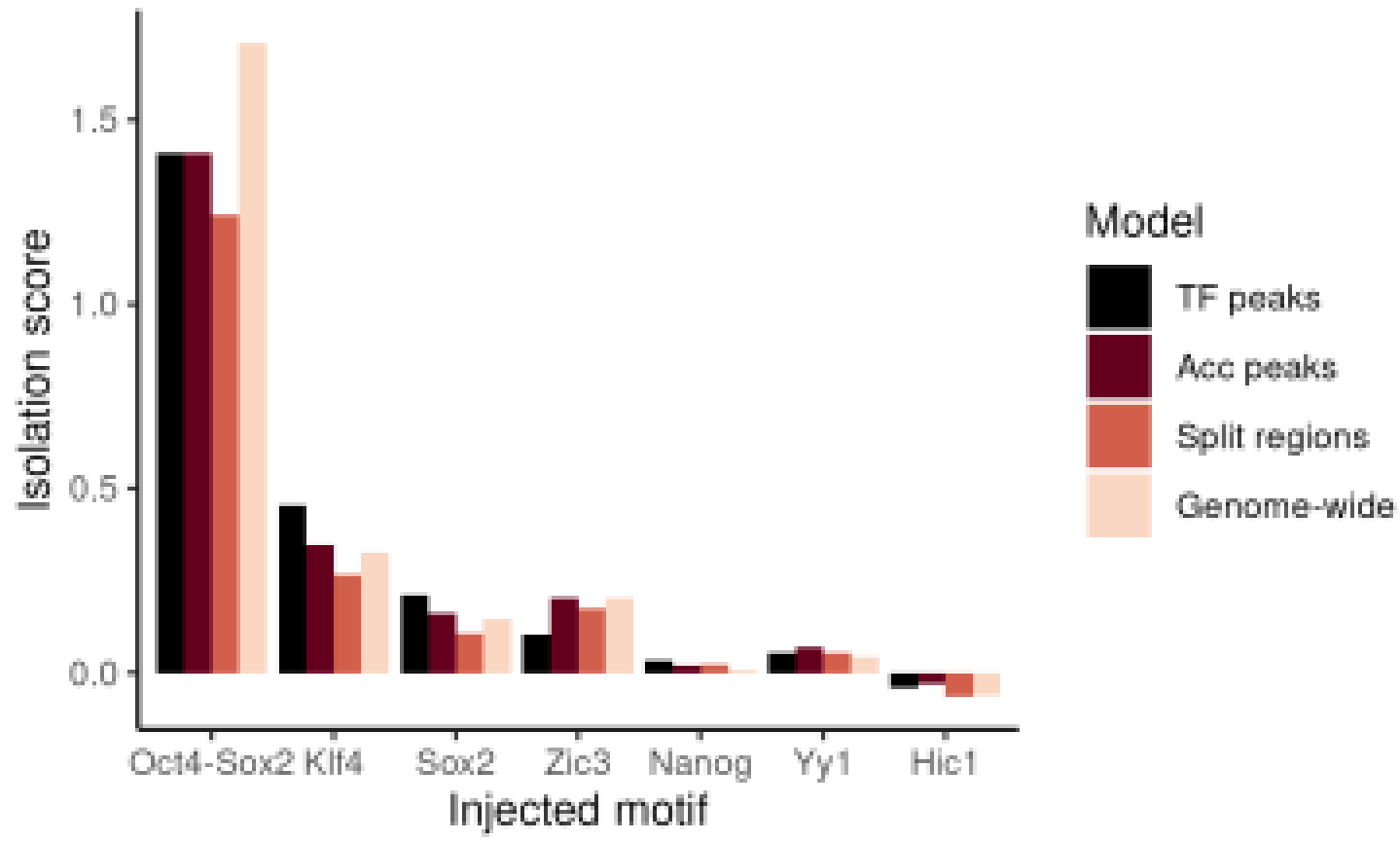

2.8. [Minor, title] Not the best choice of title. It suggests that low-affinity sites are responsible for altering regulatory potential when in fact the study finds that this is not specific to low-affinity sites but rather due to combinatorial motif presence, so why not say that motif cooperativity is the main driver of altered regulatory potential? The regulatory amplitude (Fig 6) is in fact larger for high-affinity sites, so I'm not sure why the title emphasizes low-affinity sites? Similarly, the CRISPR validation experiments target high-affinity sites.

We thank the reviewer for this thoughtful comment! Indeed, the title encompasses two findings, the comprehensive mapping and widespread cooperative role of low-affinity motifs in chromatin accessibility, and the influence of cooperativity on the regulatory potential, a finding that emerged unexpectedly when modeling and analyzing the data.

We agree that our previous title did not fully convey this, and we have now changed the title to "**Cooperativity explains the widespread role of low-affinity motifs in chromatin accessibility and increases regulatory potential**". Again, we thank the reviewer for inspiring us to rethink the title.

2.9. [Minor, clarifications on analysis type] At times it is confusing whether comparisons use in-silico predictions of accessibility and binding or the actual experimental data. A consistent use of 'predicted' or some other proxy would help.

Agreed. As mentioned in Section 2.2, we decided to modify the figures with markers of whether each one addresses deep learning predictions, kinetics modeling simulations, or experimental results.

Reviewer #3

The authors present a study on low-affinity binding sites, elegantly combining deep learning model predictions and motif interpretation with targeted experiments and thermodynamic modeling. They show the importance of low-affinity binding sites using mouse embryonic stem cells as a model (mESCs), and as such convey an important conceptual advance. The authors start very broadly, stating that they would study pioneer transcription factors, defining pioneer TFs as any TF that has a measurable effect on chromatin accessibility, this is a great approach. They also present the unsolved issue of understanding "mechanisms by which enhancers function in vivo", this is an important question to address. They state the issue of lack of interpretation of deep-learning sequence-to-function models, this is indeed true. They start off with a global analysis of BPNet predictions of chromatin accessibility in mESCs and get all the way to thermodynamic modeling of TF binding based on these models. This is impressive.

We thank the reviewer for this positive assessment!

Thus, overall they study asks all the right questions and make the right choice of global analyses. Unfortunately, they then don't fully deliver on their promise of unbiased global analysis and restricted their analysis a set of 5 very well-known TFs in mESCs.

We thank the reviewer for the constructive feedback, which we took as an opportunity to showcase some more results in our revised manuscript. Specifically, we have included pairwise interactions of all discovered pioneer motifs, expanded the single/grouped motif arrangements to gene expression data, and applied a similar analysis on data of neuronal differentiation, as suggested.

Thus, I have four major comments:

3.1. [Expansion of motif pairs] When the authors state "We then calculated the in silico cooperativity scores for all motif pairs across the genome[…] 4C" - are these all the motif pairs that were found? Or all the motif pairs of the 5 TFs that were analysed in more detail? Can they do this globally across all motifs?

Our accessibility model indeed found several additional motifs contributing to accessibility (Supplementary Figure 1C), but we purposely limited our analysis to the five pluripotency TFs where we have high-quality ChIP-nexus data with precise binding footprints for these TFs. ChIP-nexus data allowed us to to reliably assess the validity of low-affinity motifs as we did for example with the very clear footprints Oct4 and Sox2 (Figure 1E). We have clarified the text in the **Results** section to be more precise (see below).

We agree however with the reviewer that we can readily predict the effects of all other mapped accessibility motifs, which we have now done for the interested readers. As in Figure 4C, we have calculated the pairwise motif accessibility cooperativity scores for all

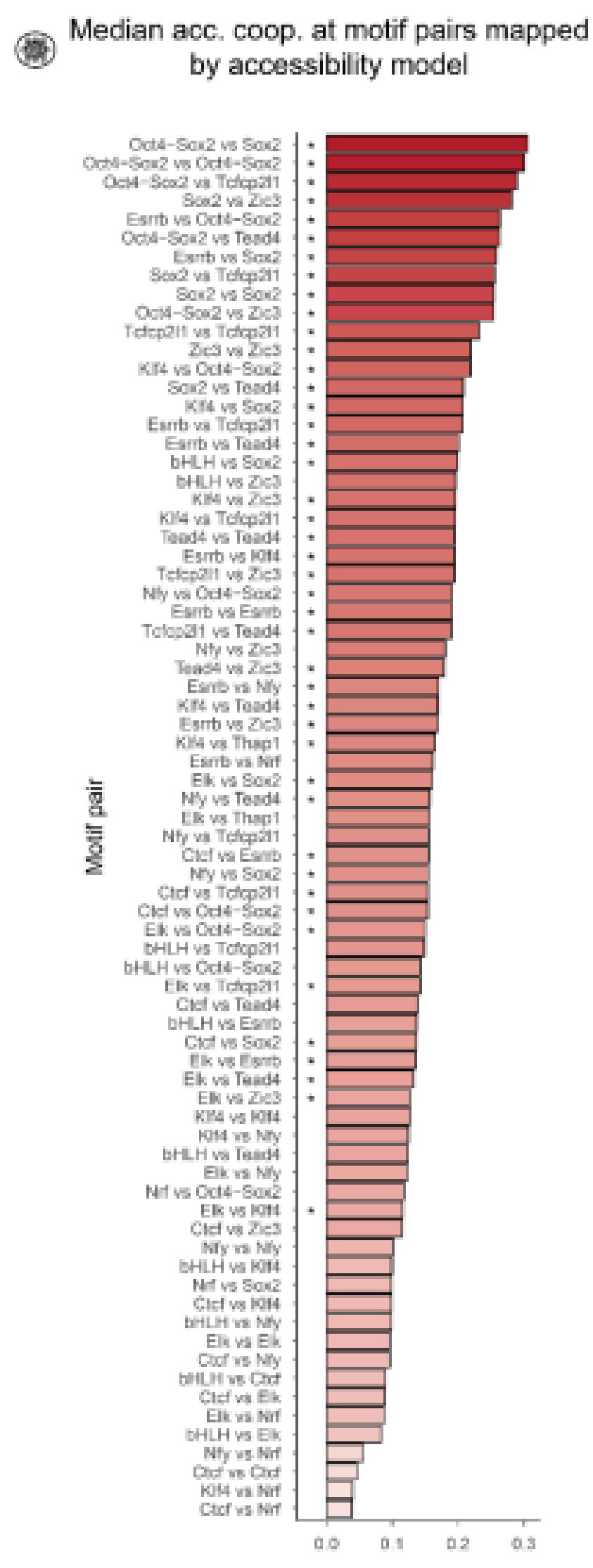

cross-validated motifs mapped by the accessibility model. The plot on the right is now added to Supplementary Figure 4 with the added **Results** section:

pg. 9-10: "**We then analyzed the cooperativity between our five pluripotency TFs. We found that all pairs of pioneer motifs showed cooperativity substantially above 1 (or above 0 in log-space) (Figures 4C, S4A-B, Table 6), while the cooperativity was weakened or non-existent when one motif was a non-pioneer motif, such as *Nanog* (Figure S4C). This was true across all accessibility models, thus was robust to the training region selection. Furthermore, similar syntax was found for the additional cross-validated motifs beyond those bound by the five pluripotency TFs (Figures S4D and S1C).**"

3.2. [Gene expression plots] Given the powerful pipeline that the authors present about BPNet predictions, deep understanding of motifs and cooperativity, I would have expected the authors to investigate some of the more functional effects on cooperative binding - e.g. on enhancer function in terms of expression. The authors specifically choose a very well-studied system (mESCs), so they could make use of these data that have been collected for the cell line (or produce their own).

We agree that the regulatory consequences of pioneer cooperativity on gene expression is an important question and an active area of investigation. In our system, we indeed find that pioneer cooperativity in *Oct4-Sox2* motif arrangements affects gene expression, albeit only at proximal enhancers. As with accessibility (Figure 6E) and local enhancer expression (Figure 6F), we find that target gene expression is affected more strongly by *Oct4-Sox2* motifs in grouped arrangements rather than isolated arrangements when the *Oct4-Sox2* motif is found within 5 kb of the transcription start site. Considering the reviewer's comments, we have now put these results into the manuscript alongside pertinent controls (**Figure 6G** and **Supplemental Figure 6I**).

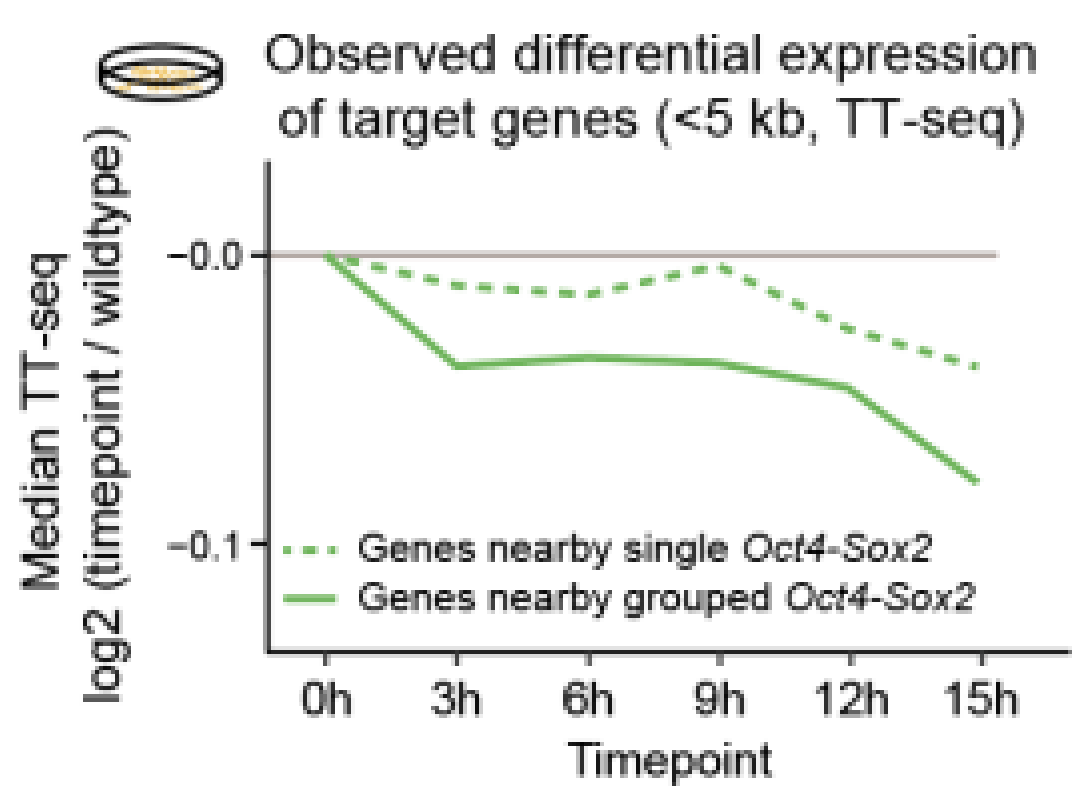

3.3. [Neuronal differentiation] , the study is lacking any prediction of how all these CRE-grammar and cooperativity actually matter. For example, can the authors now predict how cells would adapt to changes and how cooperative binding or low-affinity motifs may be important for cellular function or differentiation? mESC are a great model for looking at differentiation. Along this line, one suggestion is to make use of data from a recent study by Bunina et al (https://doi.org/10.1016/j.cels.2020.05.003) - that has investigated Sox2 cooperative binding in mESCs differentiating to neurons and collected ATAC (as well as RNA and proteomic) data along a differentiation time course. This study suggests that Sox2 cooperatively binds with Oct4 as long as Oct4 is expressed and starts partnering with other TFs (such as Atrx) upon differentiation. The model the authors present would be a perfect tool to mechanistically investigate some of these aspects, and more generally, how cooperative TF binding may mechanistically underpin cellular differentiation.

We thank the reviewer for this excellent suggestion. We have now performed a similar analysis using the data and findings from Bunina et al (shown in **Figure 6H-J** and described in the **Results**, **Supplementary Tables** and **Methods).** We trained ChromBPNet models over this neuronal differentiation time course and performed similar analyses to the original Figure 6. We found that during neuronal differentiation, Oct4 expression decreases alongside the *Oct4-Sox2* motifs, while motifs such as the *Rfx4* motif associated with neuronal differentiation (Choi et al 2024 Cell Mol Life Sci) become more important.

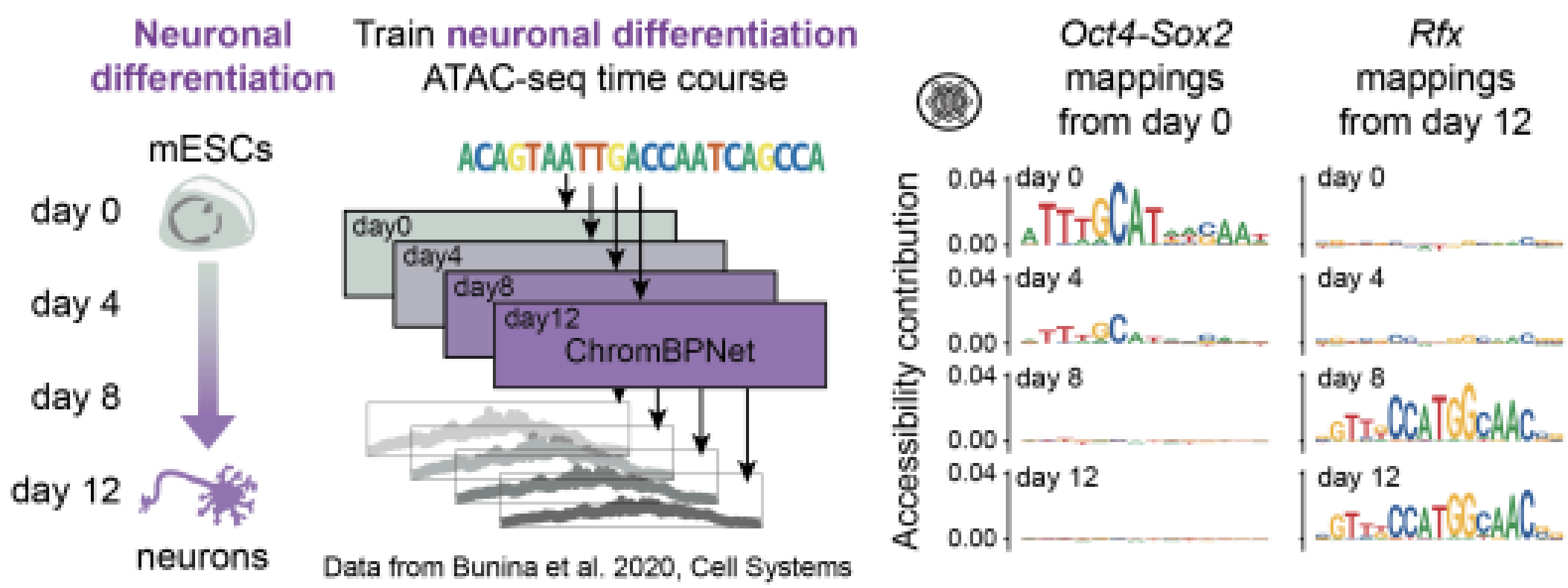

We next examined whether grouped/isolated high/low-affinity *Oct4-Sox2* motif arrangements follow the same properties as our Oct4 depletion time course from Xiong et al in Figure 6. As shown below, grouped arrangements had a more rapid loss of accessibility over time.

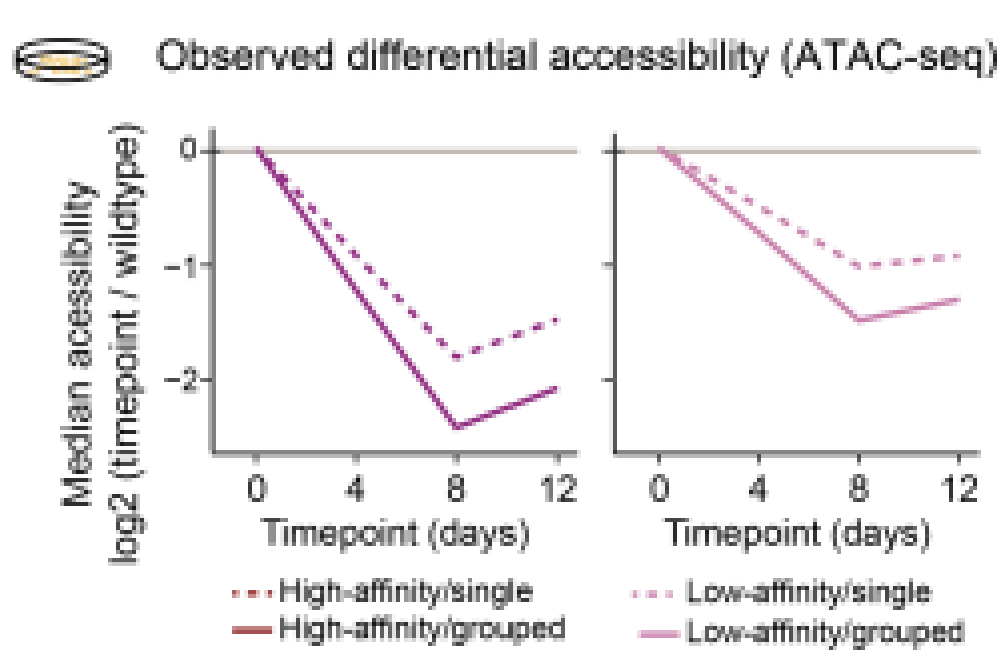

We again thank the reviewer for their suggestion to incorporate additional contexts by which pioneering cooperativity manifests, since we believe it plays integral roles in directing cell fate, disease, and evolution.

3.4. [CTCF's role in low-affinity cooperativity] Finally, the authors view the genome as one-dimensional while 3D confirmation is important for enhancer function as well. In that context, one TF that is very obviously missing is CTCF, as it does affect chromatin accessibility. Investigating the relationship with CTCF would also alleviate the current issue that the authors treat the (3D) genome as a one-dimensional. Getting back to CTCF - do the low-affinity binding sites interact with CTCF? Is there reason to believe they would be involved in - or not involved in - shaping 3D chromatin architecture?

Excellent question, which prompted us to add Figure S4 to the manuscript. We do believe that pioneering cooperativity at an individual locus (1D) could influence the capacity of CTCF for higher-order chromatin conformation (3D). Evidence suggests that cell-specific pioneer TFs can facilitate CTCF binding occupancy (Liu et al 2023, Cell Reports; Lorzadeh et al 2025, Nat. Comm), which in turn appears to stabilize its corresponding regulatory loops and hubs (Liu et al, 2023 Cell Reports). We have now incorporated this idea into **Supplementary Figure 4**, described below.

As shown above in Section 3.1, the additional accessibility cooperativity predictions that are part of the revised manuscript also show that the *CTCF* motif cooperates with other pioneering motifs to increase local chromatin accessibility. To expand on this idea, we examined how other motifs of various affinities were predicted to cooperate with CTCF (n=7,363) and found that *CTCF* motifs were predicted to readily cooperate with both high- and low-affinity pioneering motif partners. Thus, low-affinity pioneering motifs could be involved in shaping 3D chromatin architecture. The plot was added to **Supplementary Figure 4**:

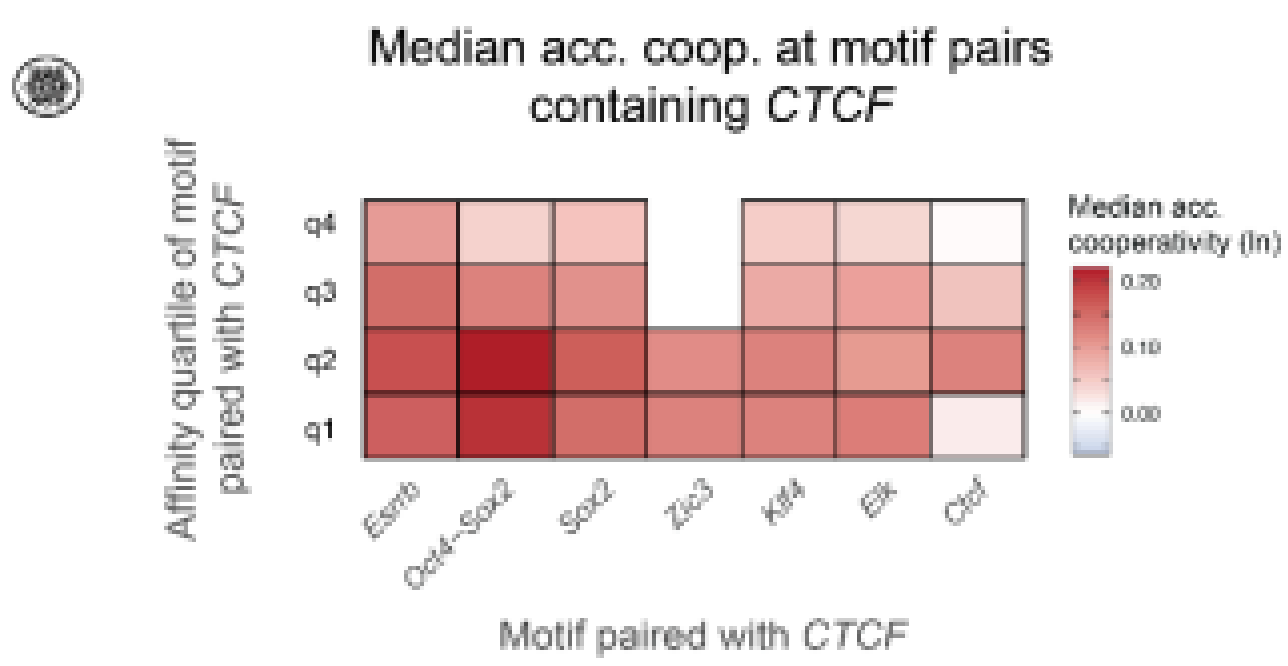

In the revised version, we now discuss the potential role of low-affinity motifs in initializing chromatin accessibility to enable chromatin organizers such CTCF to bind. An excerpt of the expansion is below:

pg 9-10: "**Furthermore, similar syntax was found for the additional cross-validated motifs beyond those bound by the five pluripotency TFs (Figures S4D and S1C). Even CTCF, a key regulator of 3D chromatin organization, was subject to cooperative boosts from pioneer motifs (Figure S4E), consistent with previous evidence (Liu et al, 2023 Cell Reports). This suggests that cooperativity is very common and perhaps the default among pioneer TFs.**"

While our ability to interpret CTCF's role is limited to predictions in this work, we hope that future research addresses the role of low-affinity motifs in chromatin organization more directly.

3.5. [Minor, F3E,F bumpy accessibility profile] Figure 3E,F, what are the pattern in accessibility? Seems to small for nucleosomes

The reviewer is correct, this is not nucleosome-mediated phasing, which is clear when shown side-by-side with unpublished mESC MNase-seq nucleosome data from our lab:

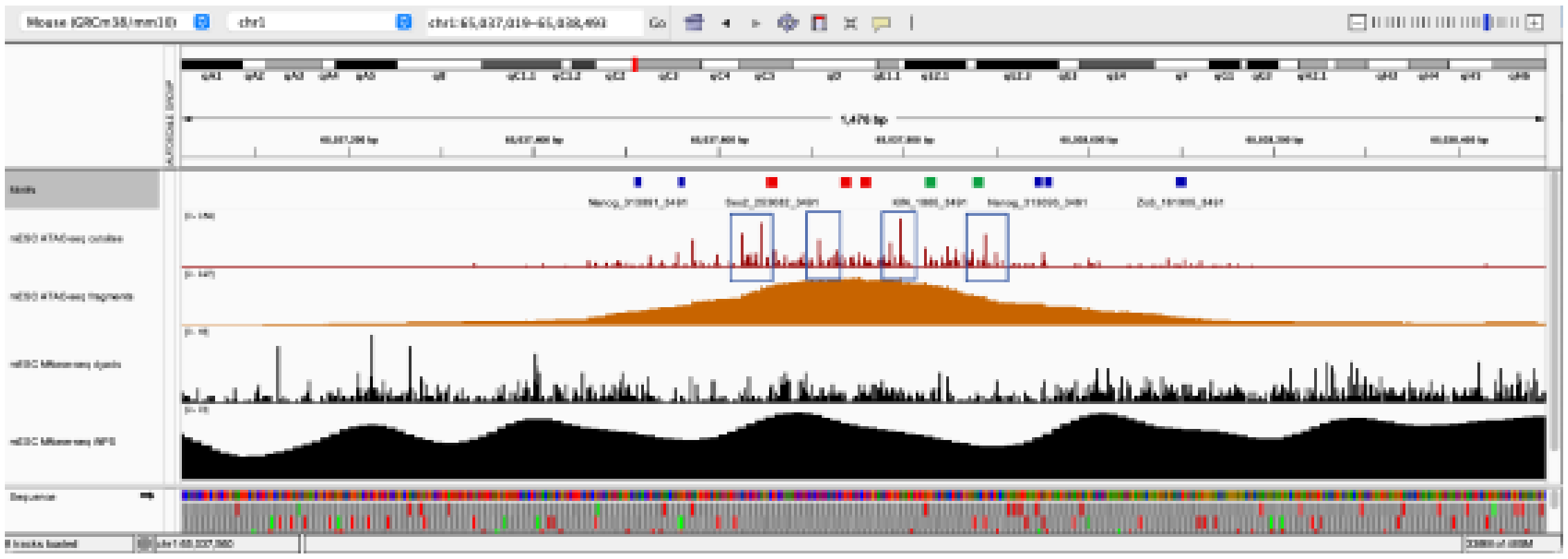

We depict ATAC-seq accessibility at individual loci as ATAC-seq cut site coverage (red track) since this is the data that ChromBPNet is trained on (Pampari et al 2025, biorXiv) and best reveals TF footprints (though also subject to Tn5 sequence bias before bias correction). Thus, the "bumpy" pattern (blue boxes) across the ATAC-seq cut sites (red track) represents the boundaries of TF binding to the *Sox2* motifs (red motifs) and *Klf4* motifs (green motifs). When ATAC-seq is displayed as lower-resolution fragment coverage (orange track), these differences in footprinting are missed. For reference, mESC MNase-seq is depicted as fragment midpoints to proxy dyads (black upper track) and iNPS-smoothed fragments (black lower track).

3.6. [Minor, model prediction limitations] Effects for validations often don't fully recapitulate the predictions but go in the same directions : e.g. p8: "These results differed slightly from the predicted effects (Figure S3F), but confirmed […]" Or p9: "While the observed effects differed slightly from the predicted effects, […] (Figures 4B and S1K)." Do these discrepancies with the predictions point at specific / systematic weaknesses of the model that can be addressed in the futures?

These discrepancies do indeed point to systematic weaknesses of sequence-to-function models, including BPNet and ChromBPNet. The first limitation is addressed in the Study Limitations, namely the idea that mutating motifs, especially combinatorially (Figure 4B) is possibly pushing these models to make out-of-distribution estimates of effects. In order to reliably predict higher-order effects of TF binding motifs, deep learning models require a sufficiently diverse set of motif affinities and arrangements to infer precise behavior. Sequence-to-function models are typically trained on genomic data, thus are limited by the motif complexity that the genome has to offer. Overcoming this obstacle is an ongoing investigation for many computational labs (Yu et al 2024, Bioinformatics).

A second possible limitation is that our ChromBPNet and BPNet models were trained to predict motif effects on local accessibility (1 kb), thus they could have missed longer-range regulatory mechanisms. We have now expanded on this second point in the **Study Limitations**:

pg.17: "Models have been shown to robustly predict motif affinity effects and cooperativity effects, but there is a risk that predictions of multiple motif perturbations are out-of-distribution for stable model predictions. **Further, ChromBPNet and BPNet sequence-to-function models are designed to predict local regulatory effects of sequences (<3 kb) and are unlikely to learn longer range impacts of chromatin organization.** To confirm the inferred syntactic rules, experimental validations were conducted *in vivo* but were limited to a few individual genomic loci."

3.7. [Minor, Discussion] Another study to integrate into the interpretation is Baderna et al (https://www.biorxiv.org/content/10.1101/2025.03.26.645413v1), which uses not only DNA-sequence context but also chromatin context (modifications) to interpret cooperative and non-cooperative TFs.]

We appreciated this paper when it was released, as it is consistent with our own observations (Brennan and Weilert et al 2023, Dev Cell) linking markers of enhancer activation to increased chromatin accessibility at enhancers. We particularly enjoyed that this work was conducted through the lens of single-molecule footprinting and have since **cited Baderna et al** in this work.

Referees' report, second round of review

Reviewer 1: The authors performed a significant number of new analyses. All concerns have been fully addressed. I want to congratulate the authors on an important study.

Reviewer 2:

The authors did a great job rewriting the paper. The concept of motif cooperativity and how it elevates the relevance of low-affinity sites is stated more explicitly now, with clear distintions of both concepts and how they feed into each other. I like the schematics to differentiate predictions and experiments. They are important, so readers can easily assess which model predictions are supported by experimental evidence and which ones are not.

While I'd still prefer direct experimental evidence for TF cooperativity and how it elevates lower affinity binding sites, I understand that such efforts may be beyond the scope of this study. Overall, the additional clarifications, analyses and experimental datasets (e.g. Bunina et al) strengthen the study and show that the prediction-based classifications translate to measurable differences in accessibility that is reproducible across different studies and model systems.

The authors sufficiently addressed the model training aspect (also raised by reviewer 1) and I am also content with the new presentation of the kinetics model.

While limited in experimental scope, this study should be of great interest to the regulatory genomics field and definitions of key findings as well as their limitations are now presented throughout the paper.

Reviewer 3:

The authors have addressed all my comments and significantly strengthened the manuscript by 1) providing predictions on cooperativity of all pioneering factors and 2) by providing more functional interpretations of the models in terms of how low-affinity cooperativity may affect gene expression also during differentiation. I congratulate the authors on an excellent study.

Authors' response to the second round of review
